# Transmission of apple stem grooving virus (*Capillovirus mali*) to apple from the soil-borne fungus *Fusarium solani*

**DOI:** 10.1186/s12870-025-07188-0

**Published:** 2025-09-29

**Authors:** Yan Xia, Bo Li, Xueli Wu, Zhonghai Han, Kunxi Zhang, Pengbo Hao, Miaomiao Wang, Yujie Zhao, Yu Liu, Jiangli Shi, Ran Wan, Wanyu Xu, Liu Cong, Yawen Shen, Jian Jiao, Xianbo Zheng

**Affiliations:** 1 College of Horticulture, Henan Agricultural University, Zhengzhou, 450046 China; 2 Henan Apple Germplasm Innovation and Utilization Engineering Research Center, Henan Agricultural University, Zhengzhou, 450046 China; 3 International Joint Laboratory of Henan Horticultural Crop Biology, Henan Agricultural University, Zhengzhou, 450046 China; 4 Henan Provincial Forestry Ecological Construction and Development Center, Zhengzhou, 450003 China

**Keywords:** Apple, Virus, *Fusarium solani*, ASGV, Transmission, Cross-kingdom

## Abstract

**Background:**

Viruses can affect the growth, yield and fruit quality of apple trees. Apple viruses are primarily spread through grafting and pruning, with limited evidence of natural transmission. Notably, wild apple trees have also been reported to carry viruses found in orchards, but the source of infection remains unknown. Plants are frequently co-infected with fungi and viruses, and recent studies have shown cross-kingdom bidirectional transmission of viruses and viroids between plants and fungi, suggesting that fungi may serve as reservoirs for certain phytopathogenic viruses. These facts raise the question whether fungi can also acquire apple viruses and serve as their hosts or vectors.

**Results:**

Tree roots and rhizospheric soil were collected from an old apple orchard to isolate fungi. Twelve fungal species were identified and subjected to apple virus detection. *Fusarium solani* was shown to be a natural host of apple stem grooving virus (ASGV) which was transmitted vertically to its asexually produced spores and horizontally to ASGV-free *F. solani* culture. The presence of ASGV in the *F. solani* root infection negatively affected apple plant growth and ASGV was systemically transmitted to the apple seedlings from the *F. solani* root infection.

**Conclusions:**

This study identifies *F. solani* as a novel vector for the transmission of ASGV. This previously unreported mode of transmission for an apple virus provides new insights for the development of effective disease management practices in apple cultivation.

**Supplementary Information:**

The online version contains supplementary material available at 10.1186/s12870-025-07188-0.

## Introduction

China is currently the largest producer of cultivated apple (*Malus* × *domestica* Borkh.) in the world [1] and is also an important centre of origin with a long history of cultivation [2, 3]. Apple orchards are affected by several viral diseases [4], with apple stem pitting virus (ASPV, *Foveavirus mali*), apple stem grooving virus (ASGV, *Capillovirus mali*) and apple chlorotic leaf spot virus (ACLSV, *Trichovirus mali*) as the main latent viruses infecting apple [5, 6]. These viruses do not cause apparent symptoms when infecting most commercial apple cultivars [7–9]. However, the infections can reduce vigor, slow nutrient absorption in roots, induce graft incompatibility [10] and increase susceptibility to other phytopathogenic agents, thus leading to significant losses in fruit yield and quality [11–13], particularly with co-infection [14]. Apple cultivars are clonally propagated by grafting to maintain fruit traits, so viral diseases are usually transmitted by propagation and mechanical pruning in the field [15, 16]. The maintenance of virus-free planting material is pivotal in preventing spread of apple viral diseases worldwide [17–19].

To date, no insect or other natural vector has been identified for the three aforementioned apple viruses, although all of them have a broad host range. ASGV and ASPV have been reported in wild apple trees growing in forests, but limited information is available on the natural spread of apple viruses [20]. Research has shown that apple viruses can be transmitted through soil, particularly when healthy plants are cultivated in soil containing virus-infected residues. Viruses can survive in the soil for weeks to months, but it remains unclear how these viruses persist [21]. One probable explanation is an unidentified viral transmission pathway involving soil-borne microorganisms associated with apple roots. Such mechanisms of virus spread have already been documented in cereal crops [22], tobacco [23] and potato [24].

Plants are in continuous contact with soil-inhabiting microorganisms, including bacteria and fungi. These interactions can be endophytic or epiphytic associations [25], which can have positive or negative effects on host health and productivity [26, 27]. Fungi are natural hosts of mycoviruses [28], and also serve as vectors for some plant viruses [23]. Fungal species in the genera *Olpidium*, *Polymyxa* and *Spongospora* are not only root pathogens but also vectors of more than a dozen soil-borne plant viruses that pose serious threats to many crops [29]. A recent study revealed that *Rhyzoctonia solani* can acquire cucumber mosaic virus (CMV) from host plants harboring the virus and can subsequently transmit CMV to virus-free plants [24]. Additionally, plant viroids have been shown to infect and replicate in several phytopathogenic fungi [30]. Metatranscriptome-based surveys of viromes have shown that many fungal-restricted lineages of RNA viruses are genetically related to viruses of plants [31], vertebrates, and invertebrates [32]. A relevant study showed that ASGV can be transmitted to pear (20% infectivity) and beans (35–90% infectivity) via inoculation of ASGV-positive *Talaromyces flavus* onto leaves [33]. Pear and bean plants inoculated with ASGV-infected *T. flavus* developed symptoms similar to those observed in plants mechanically inoculated with crude saps of ASGV-infected quinoa.

Numerous studies show that some plant viruses are bidirectionally transmitted between plants and fungi [34, 35]. It seems reasonable that fungi in the rhizosphere may be putative carriers of apple viruses. Therefore, this study began with a screen of soil-inhabiting fungi that may harbor apple viruses. RNA extracted from the fungal isolates were tested for viral genetic material. Four *F. solani* isolates were identified that were infected with ASGV. The ASGV-carrying *F. solani* can transmit the virus to healthy plants. This study is an important addition to our knowledge that may impact orchard practices such as replanting old orchards.

## Materials and methods

### Sample collection in the field

Roots, leaves, and rhizospheric soil were collected from a 25-year-old ‘Royal Gala’ apple tree maintained at the National Fruit Tree Germplasm Repository, Zhengzhou Fruit Research Institute, Chinese Academy of Agricultural Sciences (CAAS) in China (35º 36′ N, 113° 42′ E). All samples were transferred to the laboratory at room temperature for subsequent experiments. Part of the apple leaves and roots were frozen using liquid nitrogen and then stored at −80 °C. Fine roots (0.1–0.2 cm in diameter) were sampled from the top 20–40 cm of the soil layer at the beginning of fruit ripening. Soil was simultaneously collected. The loosely attached soil particles were shaken off before the thin soil layer, tightly adhered to the roots, was obtained using a sterilized brush. This rhizospheric soil sample was sieved through a 2-mm mesh.

### Isolation, purification and identification of fungi

The endophytic fungi were isolated from apple roots according to the previously described method [36]. Potato dextrose agar (PDA) medium containing chloramphenicol (30 mg/L) was used for culturing. Individual colonies were transferred to new PDA plates and incubated for 3‒5 days at 28 °C and subcultured twice to avoid plant tissue contamination. Morphologically distinct colonies were selected, and mycelial plugs were transferred onto cellophane-overlaid PDA plates. After 5 days of culture at 28 °C, the mycelia were collected for total RNA or genomic DNA extraction.

Rhizospheric soil samples (2 g) were placed into a sterile 50-mL tube containing 20 mL of 1 × PBS buffer. The tube was vortexed at maximum speed for 15 s and then shaken at 180 rpm for 5 min. The resulting turbid suspension was filtered through a sterile 100-µm nylon mesh to remove plant debris and soil particles. The sample was divided, with 1 ml of filtrate serially diluted and used for fungal isolation using culture plates as described above.

Fungal DNA was extracted from the isolates using the E.Z.N.A. Fungal DNA Mini Kit (Omega Bio–Tek, USA) according to the manufacturer’s instructions. The entire fungal ITS region and large-subunit (LSU) D1/D2 rDNA sequences were amplified using the universal fungal primers ITS1F (CTTGGTCATTTAGAGGAAGTAA) [37] and LR3 (CCGTGTTTCAAGACGGG) [38]. The amplified DNA was purified then submitted for Sanger sequencing at Sangon Biotech (Shanghai, China). The sequencing results were compared using BLAST search against the NCBI/GenBank database.

### Virus detection

Total RNA was extracted from plant materials and fungal cultures using TRIzol Universal reagent (Tiangen, China) according to the manufacturer’s instructions. For the soil sample, each sample was homogenized, dried at ambient temperature, and sifted through a 2-mm mesh to remove gravel and plant debris. Aliquots of soil (1 g) were placed into sterile 10-mL tubes containing 5 mL of 1 × PBS buffer and mixed by vortex at maximum speed for 30 s. The resulting turbid suspensions were filtered through a sterile 100-µm nylon mesh to further remove plant debris. After centrifugation at 13,500 × g for 10 min, the pellets were used for RNA extraction with an RNA Power Soil Isolation Kit (MoBio, USA) according to the manufacturer’s instructions. To increase the RNA concentration for downstream applications, three independent replicates per soil sample were used for total RNA extraction, and the extracted RNA was pooled together, purified and concentrated using a Monarch RNA Cleanup Kit (NEB Biolabs, USA). cDNA synthesis was performed using a SuperScript III First-Strand Synthesis System Kit with a mixture of random and oligo(dT)_20_ primers (Vazyme, China). Tests for 5 apple viruses were carried out using reverse transcription‒polymerase chain reaction (RT‒PCR) as previously reported [39]. The virus-specific primer sequences and expected amplified fragment sizes are given in Supplemental Table S1. The PCR products were subsequently sequenced and compared with sequences available in the GenBank database.

### Construction of full-length infectious clones of ASGV

cDNA clones of ASGV were produced on the basis of a previous report [40]: The complete viral genome was amplified from the cDNA of the ASGV**-**harboring fungi or apple leaves, using the primers ASGV-F (TAACAGGCTTAATTTCCGCGCTTTACGTCAA) and ASGV-R (CAAACTCTAGACTCTAGAAAAACCACAC). Amplicons were gel-purified, inserted into the pGEM-T easy vector (Promega, USA), and transformed into *Escherichia coli* JM109 to obtain recombinant plasmids, then used as templates to produce PCR fragments containing the T7 promoter sequence with the primers T7-GV-F (TAATACGACTCACTATAGGGCGAATTAACAGGCTTAATTTCCGCGC) and PvuII-GV-R (CCAGCTGAAACTCTAGACTCTAGAAATTT-(T)_18_). The fragments were subsequently used as substrates for synthesizing ASGV infectious transcripts with an *in-vitro* transcription kit (RiboMAX™ large-scale RNA production system, Promega, USA) according to the manufacturer’s instructions. These plasmids were also submitted for Sanger sequencing at Sangon Biotech (Shanghai, China). The sequences were aligned and subjected to a phylogenetic analysis by MEGA12 using the neighbour-joining method with 1000 bootstrap replicates and bootstrap values < 60% were omitted [41].

### Acquisition of virus-free *F. solani* and inoculation of virus

ASGV-free *F. solani* (referred to as ASGV^free^) was purchased from Mingzhou Biological Company (Zhejiang, China). The lyophilized fungus was resuspended in sterilized water and then cultured on PDA medium containing chloramphenicol. A fungal mass growing at the edge of the culture medium with a diameter of 1 cm was picked and placed into the center of a new PDA medium with the mycelium side down, incubating for 7 days in an incubator at 28 °C. The in vitro-transcribed RNA transcripts of ASGV were thawed on ice for several minutes and then diluted with sterile water. After mixing by pipetting up and down, 1 mL of the RNA solution was added to a 2-mL sterilized tube containing the mycelium plug. They were gently shaken at 28 °C for 1 day. An appropriate amount of spore suspension was extracted from the tube and observed under the microscope, then pipetted the suspension onto a new PDA plate and cultured to form a complete colony at 28 °C. The RNA was extracted from the fungi employing the previously described methodology followed by the detection of ASGV to acquire *F. solani* that was carrying ASGV (referred to as ASGV^carrying^).

### Colonization of tobacco with the ASGV-carrying *F. solani*

To verify whether the *F. solani*-derived ASGV can infect a plant, inoculation using a crude extract of the ASGV-carrying *F. solani* strain (ASGV^carrying^) was carried out on tobacco leaves. ASGV is a latent virus on apples and cannot cause apparent symptoms, so *Nicotiana glutinosa* was used as an indicator plant. ASGV^carrying^ strain with medium was picked, ground and re-suspended in 1 × PBS buffer (containing 0.1% Tween 20), then mechanically inoculated on the leaves of 4-week-old *Nicotiana glutinosa* using silica sand. The ASGV-infected apple leaves were also ground, re-suspended and inoculated as positive control while the 1 × PBS buffer were inoculated as mock. Ten tobacco plants were inoculated two leaves at the bottom of per plant for each treatment. The inoculated plants were grown in growth chambers maintained at 26 °C with a 16/8 h day/night cycle, and symptoms were observed. Extracted RNA from leaves with symptom and performed virus detection by RT‒PCR using specific primers listed in Supplemental Table S1. The substrate, vermiculite and silica sand used in this part were subjected to sterilization by autoclave to exclude the influence of other microbes.

### Colonization of apple with the ASGV-carrying* F. solani*

To test whether *F. solani* can transmit ASGV to apple via root infection, virus-free apple seedlings were inoculated with the ASGV^carrying^ *F. solani* strain generated above. The ASGV^free^ *F. solani* strain was also used as inoculum as control. Mycelium plugs of ASGV^carrying^ were inoculated to potato dextrose Liquid medium without antibiotics and shaken at 28 °C for 2‒3 days. The fungal culture was subsequently filtered with sterile gauze to obtain a large quantity of mycelium. These mycelia were rinsed with sterile water to release spores. The spores were collected in 1 × PBS buffer (containing 0.1% Tween 20) and observed under microscope. The 4-week-old apple seedings (*Malus hupehensis var. mengshanensis*) were grown in autoclaved substrate, and those showing similar health and growth were selected for inoculation. The roots of 10 plants per treatment were placed in the spore suspension and soaked for 10–15 min. The inoculated apple plants were then planted in the sterilized substrate, randomly distributed in a growth chambers with a 16/8 h day/night cycle at 26 °C. The seedlings were irrigated with distilled water every 2 days.

After 4 weeks of treatment, plants from both the ASGV^carrying^ and ASGV^free^ groups were measured and photographed. The roots of each plant were scanned with an LA2400 Scanner (Regent, Canada), and several root indices were measured using the root image analysis software WinRHIZO [42]. The chlorophyll content of the plant leaves was measured according to previously reported methods [43]. Total RNA was extracted from the leaves for ASGV detection by RT‒PCR.

### Test of horizontal and vertical transmission of ASGV in fungi

ASGV^free^ (referred to as recipient) and ASGV^carrying^ (referred to as donor) *F. solani* strains were simultaneously inoculated into PDA medium and cultured for 3–5 days at 28 °C. Mycelium pieces were removed from the edge of the plate with a diameter of approximately 1 cm by a hole punch, placed into new PDA medium, cultured at 28 °C. Growth of the strains was observed every day. When the mycelia of the two clumps had fused, three random locations from the side of the intersection near the recipient strain were selected and transferred to new PDA medium and cultured for 3‒5 days at 28 °C. RNA from the subcultured recipient strains were extracted to identify the horizontal transmission of ASGV by RT‒PCR.

*F. solani* produces asexual spores during growth, and the virus spread together with the spores in the process of subculture, that is vertical transmission. Spores of ASGV^carrying^ were collected in sterile water and observed under microscope, then spotted onto a new PDA medium. After cultured for 10‒12 days at 28 °C, the spores were again collected and subcultured. This subculturing was repeated 8 times. The virus-carrying status of each generation were detected by RT‒PCR to identify the vertical transmission ability of ASGV in fungi.

### Statistical analysis

The experimental data were assessed using analysis of variance (ANOVA). The differences between means were determined by the least significant difference (Duncan) test at *p* < 0.05. SPSS version 22.0 and GraphPad Prism 10 software were used for all the statistical analyses and pictorial representations, respectively.

## Results

### Detection of apple viruses/viroids in apple plants and fungi

**Fig. 1:**
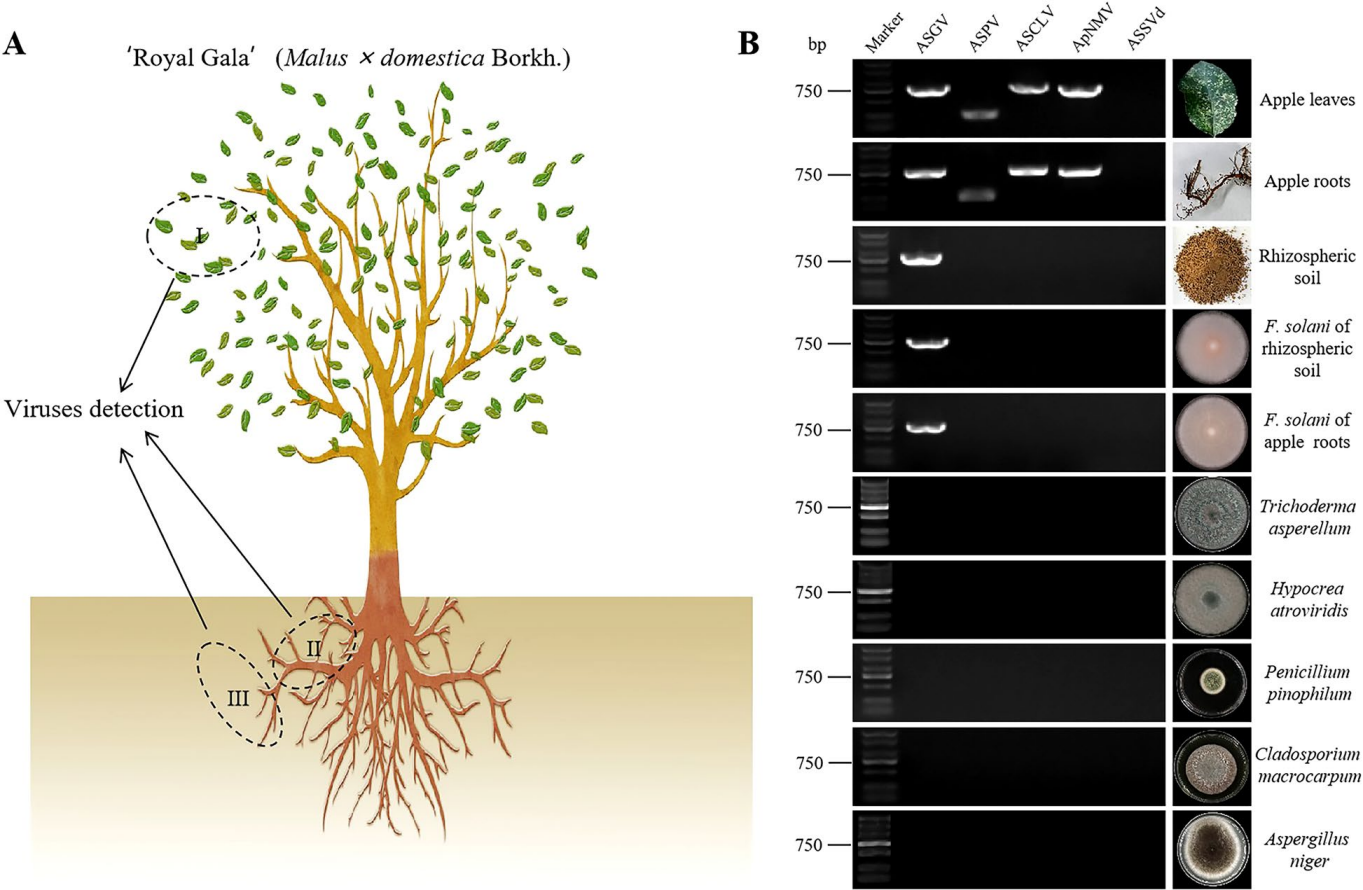
Detection of 5 apple viruses from different samples and fungal isolates. **A** Leaves (I), roots (II) and rhizospheric soil (III) samples were collected from ‘Royal Gala’ and used to detect the apple viruses ASPV, ASGV, ACLSV, ApNMV, and ASSVd. **B** RT‒PCR detection of 5 viruses in apple leaves, roots, and rhizospheric soil as well as different fungal isolates from the plant roots or soils

To assess the apple viruses-carrying status of the collected samples at the preliminary stage, 5 major apple viruses, ASGV, ASPV, ASCLV, apple necrotic mosaic virus (ApNMV, *Ilarvirus*), and apple scar skin viroid (ASSVd, *Apscaviroid cicatricimali*) were tested in RNA extracted from the leaves, roots and rhizospheric soil of a ‘Royal Gala’ apple tree (Fig. 1A). Detection results showed that the apple tree was infected by ASGV, ASPV, ApNMV, and ASCLV, but only ASGV was detected in the rhizospheric soil samples (Fig. 1B). In addition, cultures were made to isolate fungi from the roots and rhizospheric soil. In total, 12 fungal species were isolated, including *F. solani*, *Aspergillus niger*, *Rhizoctonia solani*, *Dothideomycetes sp.*, *Cladosporium macrocarpum*, *Penicillium pinophilum*, *Trichoderma asperellum*, *Hypocrea atroviridis*, *Talaromyces verruculosus*, *Fusarium oxysporum*, *Blumeria graminis*, and *Talaromyces sp.* (Supplemental Fig. 1).

To determine whether the fungi living in these surroundings also carried these viruses, RT‒PCR was conducted on RNA extracted from all isolate fungi using primer pairs targeting 5 apple viruses above. In the end, ASGV-specific amplicons were obtained from 4 isolates that were identified as *F. solani* on the basis of ITS1-5.8S-ITS2 sequences and morphological trait analysis, while none of the apple viruses were detected in the other fungi. These ASGV-positive fungal isolates were from both roots and rhizospheric soil (Fig. 1B, Supplemental Fig. 2). Subsequently, the full-length sequences of ASGV were amplified from both *F. solani* carrying ASGV and from ASGV infected apple leaves (Additional file 3), the nucleotide sequence identity of the two reached 80.95% and amino acid sequence reached 80.77%. They were both displayed as ASGV after being blasted with the NCBI database, a result consistent with findings obtained from phylogenetic analysis (Supplemental Fig. 3, red box marked).

### Verification of infectivity and pathogenicity of *F. solani*-derived ASGV

**Fig. 2:**
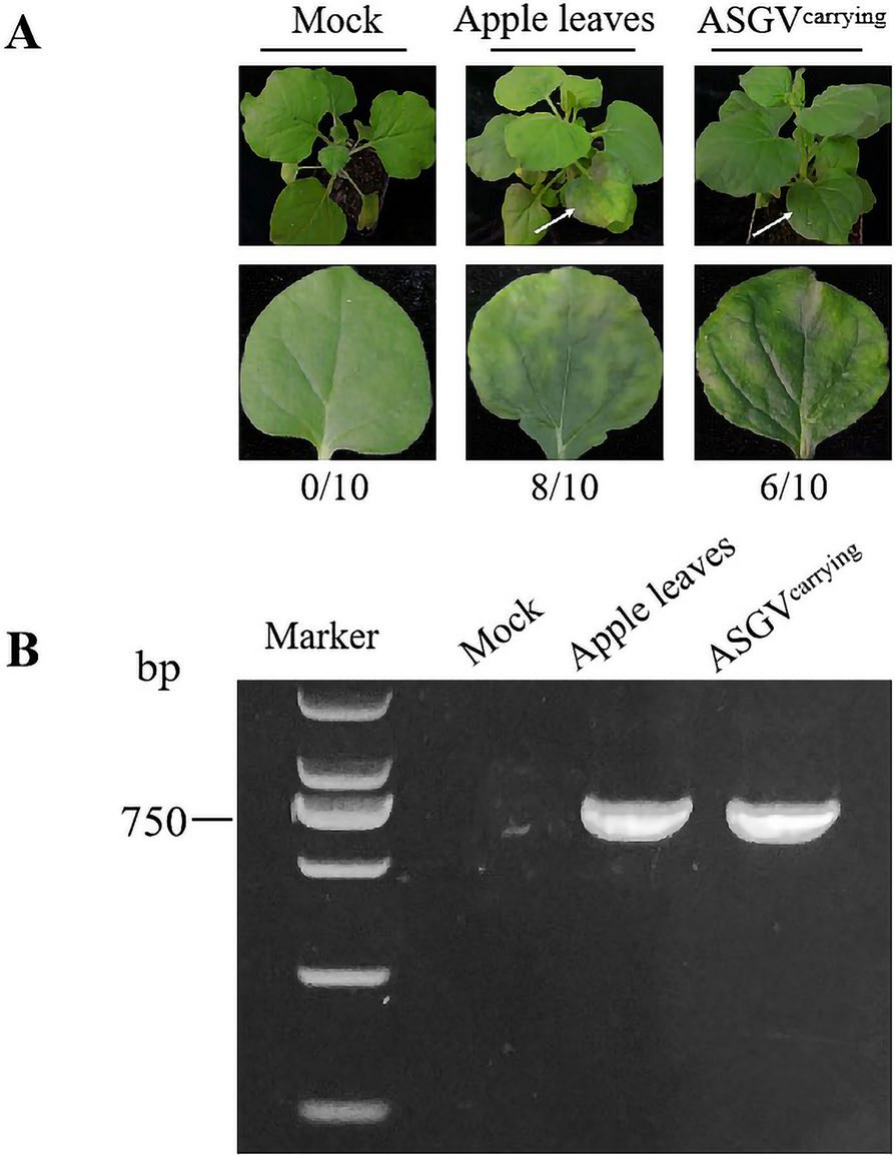
Infection of tobacco by ASGV derived from extract of the ASGV^carrying^ or ASGV-infected apple leaves. **A** Symptoms of tobacco infected by ASGV from *F. solani* harboring ASGV and from ASGV-infected apple leaves, compared to mock inoculation/1 × PBS buffer. Numbers indicate the number of tobacco plants with observed symptoms from a total of 10 inoculated seedlings. **B** Detection of ASGV DNA in tobacco leaves infected by ASGV from different sources. Mocks in A and B are leaves without abrasion inoculation

To verify the infectivity and pathogenicity of *F. solani*-derived ASGV, crude extracts of the ASGV-carrying *F. solani* strains (ASGV^carrying^) and ASGV-infected apple leaves were used to inoculate the leaves of the tobacco *N. glutinosa* with abrasion. Of the 10 tobacco plants infected with the ASGV^carrying^ fungal extract, 6 plants developed severe chlorotic yellow–green splotches after 1 week (Fig. 2A). Another 10 tobacco plants were treated with coarsely ground apple leaves infected with ASGV. The crude extract of ASGV-infected apple leaves caused the same symptoms in 8 plants (Fig. 2A). ASGV was detected in the tobacco plants infected with both the ASGV^carrying^ fungus and the ASGV-infected apple leaf extract (Fig. 2B). These findings indicated that *F. solani*-derived ASGV has biologically active infectivity and pathogenicity as transmission to tobacco was demonstrated and typical symptoms for ASGV were induced on the herbaceous host.

**Fig. 3:**
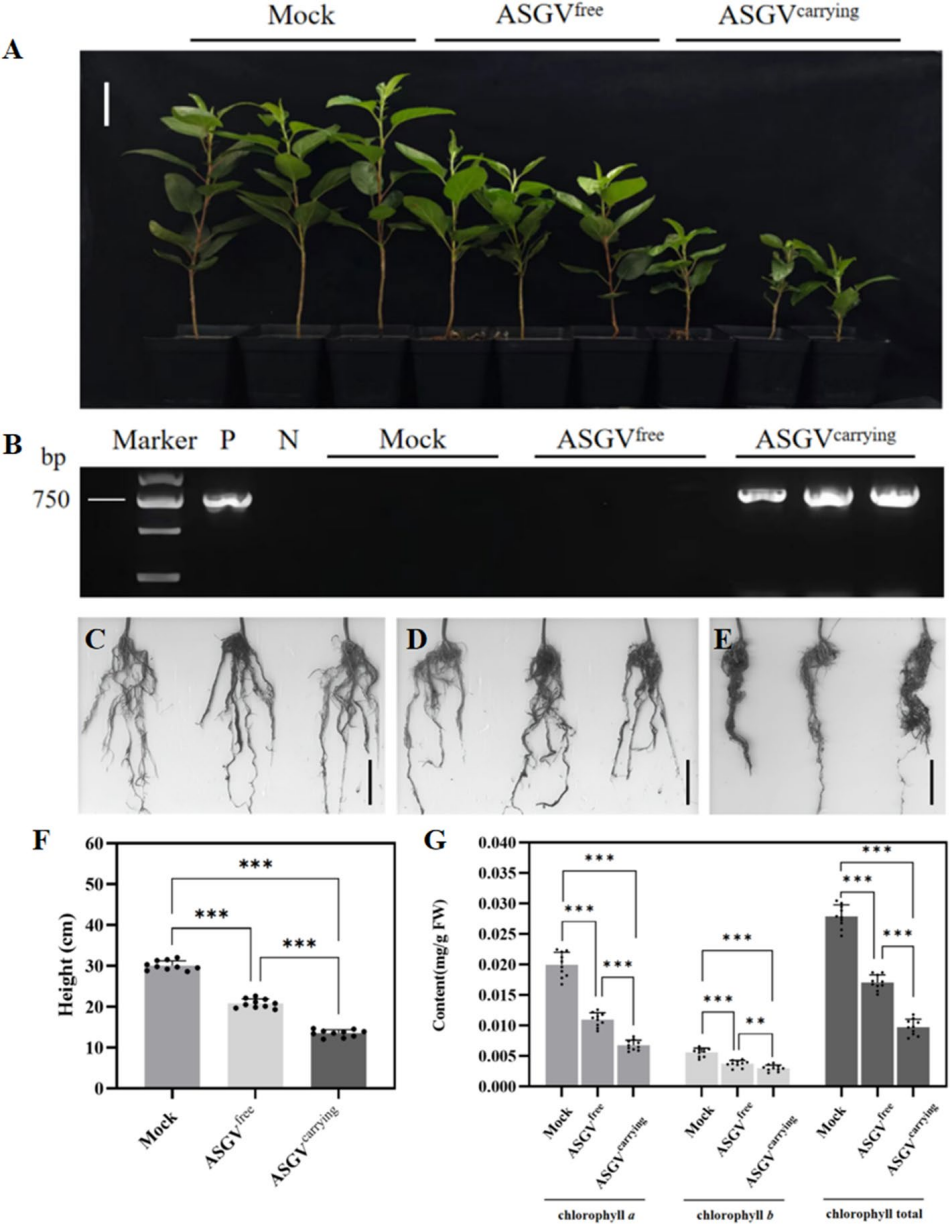
Growth of apple plants inoculated with *F. solani* spores carrying or free of ASGV. **A** Phenotypes of apple plants infected by the spores of ASGV^free^ or ASGV^carrying^ strain after 4 weeks. Bars = 5.0 cm. *n* = 10, with 3 representative plants shown for each treatment. **B** Detection of ASGV in apple leaves of plants inoculated by soaking roots in spores of different fungal strains. **C-E** Phenotypes of the root systems 4 weeks after soaking in a suspension of the PBS-Tween buffer (**C**) or buffer containing ASGV^free^ spores (**D**) or ASGV^carrying^ spores (**E**). Bars = 5.0 cm. *n* = 10 for each treatment, with 3 representative plants shown. **F** Height statistics of different treatment groups. **G** Contents of chlorophyll *a*, *b* and the total chlorophyll of the different inoculation groups. Two-tailed unpaired two-sample *t* tests were used to compare at *P < 0.05, **P < 0.01, and ***P < 0.001

Subsequently, we wanted to determine the effect of ASGV-carrying *F. solani* on apple seedlings through root infection. The roots of 10 apple seedings were soaked in either a suspension of spores from ASGV^free^ or ASGV^carrying^ *F. solani* as well as PBS-Tween buffer with no fungal spores. Compared with the mock treatment, both the ASGV^free^ and ASGV^carrying^ spores reduced the growth and vigor of apple seedlings, but the reduction was more pronounced for the ASGV^carrying^ strain (Fig. 3A and F). The RT‒PCR results revealed that ASGV was presented in leaves of apple plants inoculated at the roots with the ASGV^carrying^ spores, but not in those inoculated with the ASGV^free^ spores (Fig. 3B), suggesting that ASGV-carrying *F. solani* could transmit ASGV to apple seedlings via their roots. To exclude any effects of infection by the mycelia of *F. solani*, the leaves of apple plants were picked and cultured in PDA medium. After a period of time we observed that mycelia did not grow on the edge of the leaves of both ASGV^free^ and ASGV^carrying^ infected groups, indicated that *F. solani* did not spread systemically to the leaves of the plants (Supplemental Fig. 4).

The root systems of each infected plant were scanned and analyzed using a root scanner. Compared with the plants inoculated with ASGV^free^ spores (Fig. 3D) and the mock inoculation (Fig. 3C), the roots of the plants infected by ASGV^carrying^ spores appeared darker and smaller (Fig. 3E), indicating that the combined infection of *F. solani* and ASGV accelerated root rot. Compared with the mock inoculation, the total root surface area, root length, total root volume, root tip number, root bifurcation number, and maximum root diameter under ASGV^free^ spores infection decreased by 21.4%, 33.1%, 17.1%, 15.1%, 34.9%, and 11.9%, respectively. Under ASGV^carrying^ spores infection, these parameters decreased by 30.4%, 49.9%, 30.6%, 42.3%, 51.9%, and 28.8%, respectively (Supplemental Table S2). Additionally, the leaves of apple plants infected by ASGV^carrying^ spores appeared dry and yellow and began to fall off 20 days post inoculation. Chlorophyll content measurements showed significant differences between inoculated plants and mock-inoculated plants. The contents of chlorophyll *a*, chlorophyll *b*, and total chlorophyll in leaves inoculated with ASGV^carrying^ spores decreased by 66.0%, 46.3%, and 65.1%, respectively. While the pigments in the ASGV^free^ group decreased by 45.1%, 33.6% and 38.8%, respectively (Fig. 3G).

### Horizontal and vertical transmission of ASGV

**Fig. 4:**
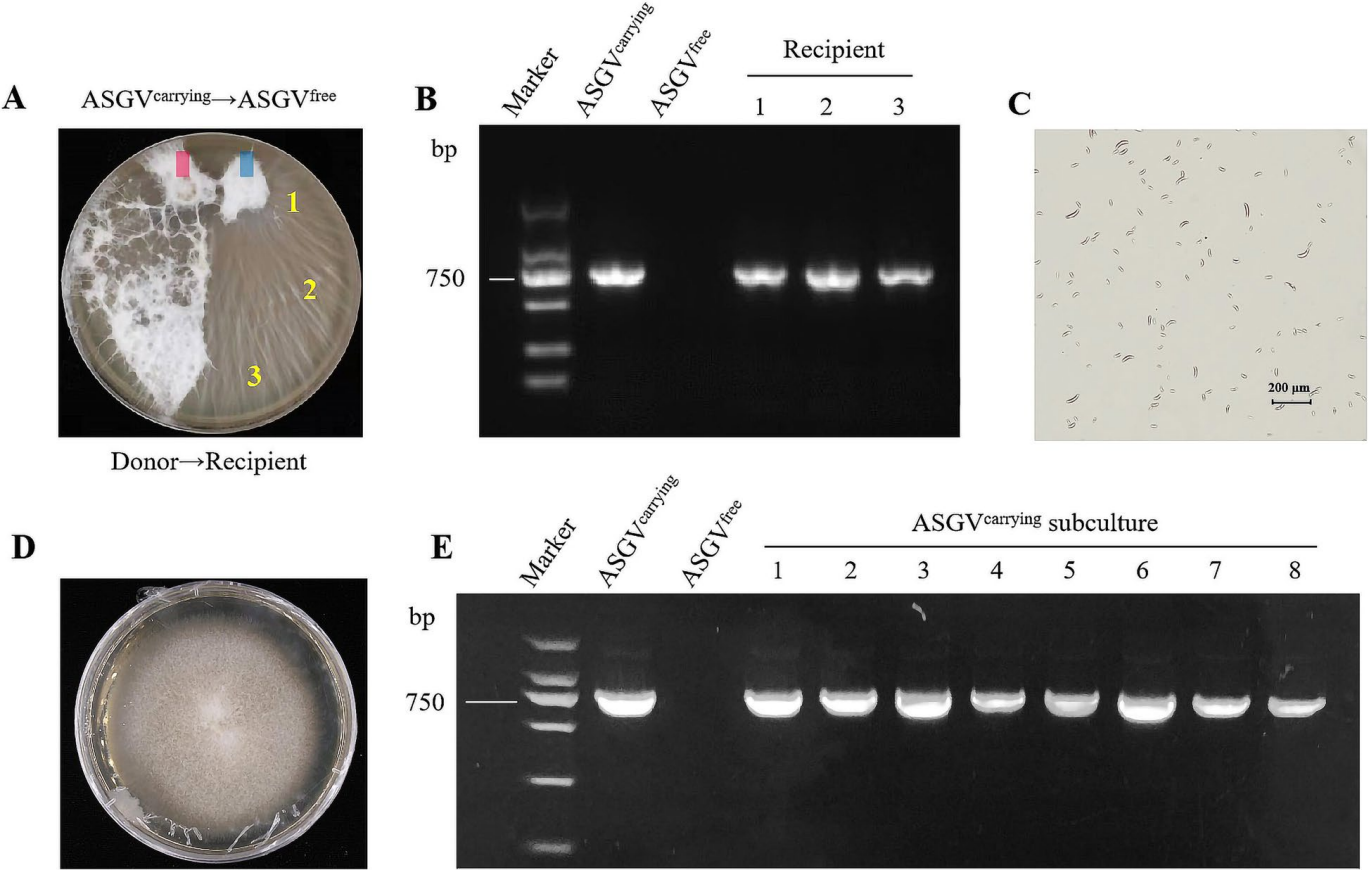
ASGV spreads horizontally and vertically among fungi. **A** Co-culture of ASGV^carrying^ and ASGV^free^ *F. solani* on the same PDA medium. Plugs were placed at the top of the plate but samples were taken from the receipeint side. **B** RT‒PCR detection of ASGV in fungal recipients. **C** A collection of ASGV^carrying^ spores as seen under the microscope. **D** Colonies formed from the collected spores of ASGV^carrying^ after subcultured for 8 times. **E** RT‒PCR detection of the virus in the successor strains arisen from asexual spore culture. ASGV^carrying^ *F. solani* was used as the positive control, and ASGV^free^ was used as the negative control

Most fungal viruses are unable to transmit or replicate outside their host under natural conditions but can move through mycelial fusion to achieve horizontal transmission in the host and vertical transmission by the production of sexual or asexual spores [28, 44]. Horizontal and vertical transmission of a virus in a fungus is likely to accelerate the spread of the virus. To verify whether ASGV can be transmitted between *F. solani*, we used ASGV^carrying^ strain as a donor and the ASGV^free^ strain as a recipient, then cultured them in the same medium. Several days later, we observed that mycelial fusion occurred between the ASGV^free^ and ASGV^carrying^ colonies (Fig. 4A). In distal samples, ASGV was detected in the mycelia selected from the recipient side (numbered locations) by RT‒PCR (Fig. 4B), indicating that the *F. solani*-derived ASGV could be transmitted to another *F. solani* strain through mycelial fusion, namely horizontal transmission. As for vertical transmission, the spores of an ASGV^carrying^ strain were collected and observed under microscope (Fig. 4C) and added to a new PDA medium to be subcultured (Fig. 4D). After repeating 8 consecutive subcultures, ASGV could still be detected in the fungi (Fig. 4E), indicating that ASGV could transmit vertically in *F. solani*.

## Discussion

### Fungal-derived ASGV sequences are similar to plant-derived ASGV sequences

In the early stage of this study, the full-length sequence of ASGV was cloned from *F. solani* and was also cloned from apple leaves. The fungal-derived ASGV (PV413210.1) was highly homologous to the ASGV isolate 13TF138 (MZ126527.1) and ASGV isolate Fuji-BJ (OP535345.1), while the apple leaves-derived ASGV (PV413211.1) was more similar to the ASGV complete sequence (NC_001749.2). The two cloned sequences exhibited a pairwise identity of 80.95%. The sequence differences between the two sources may be attributed to several factors. Firstly, there is a high mutation rate during viral replication, particularly in RNA viruses [45]. ASGV is a positive-sense single-stranded RNA virus, which tend to be prone to replication errors due to the lack of proofreading activity in its RNA-dependent RNA polymerase (RdRp). Secondly, the intracellular environment of the fungal host likely differs significantly from that of plant cells, necessitating genomic adaptations for viral survival and replication. These adaptations may include mutations or genomic rearrangements that enhance viral fitness in the new host, thereby leading to sequence divergence [46]. Despite these sequence variations, we found that the symptoms in tobacco leaves associated with the ASGV-carrying fungal isolate were similar to those associated with the ASGV-infected apple leaf extract. Studies have reported that plant viruses and fungal viruses share the same ancestor in terms of evolutionary relationship, fungi are the natural host of fungal viruses [32]. While most wild plants carry many fungi, including endophytic fungi, parasitic fungi and mycorrhizal fungi, during their growth. These fungi reproduce in the host plants during their growth [26], which can facilitate the horizontal transmission of viruses between plants and fungi. These findings indicate that the fungal-derived ASGV and the plant-derived ASGV share similar biological characteristics. We speculate that the ASGV in *F. solani* might be derived from the plant host.

### Plant viruses can infect fungi and be transmitted by fungi

*Fusarium* species are the most destructive phytopathogenic and toxin-producing fungi, causing serious diseases in almost all economically important plants [47]. *F. solani*, in particular, is a major cause of root rot in a variety of plants, causing the roots to turn black and the aerial parts to wither, eventually leading to plant death [48]. It is highly abundant in the soil of old orchards and is strongly correlated with apple root rot and replanting disease [49]. In our study, we generated an *F. solani* isolate harboring ASGV by culturing virus-free *F. solani* with the RNA transcripts of ASGV, and then verified its infectivity on tobacco and apple. Studies on fungi infected by plant viruses are common, but a few have been conducted on viruses that can infect plant pathogenic fungi [44, 50]. The first experimental demonstration of a fungus as a compatible host of plant viruses was brome mosaic virus (BMV) replication in *Saccharomyces cerevisiae* [51]. Later, a similar yeast system was successfully used to replicate other plant viruses [52]. Tobacco mosaic virus (TMV) can replicate in *Colletotrichum acutatum* [53]. There are also many plant pathogenic fungi that can support the replication of plant viruses, especially members of the alpha (+) ssRNA virus superfamily, such as CMV, TMV and BMV [54]. We observed that apple plants infected by *F. solani* harboring ASGV presented more severe symptoms, such as slower growth, poorer roots and less chlorophyll. This partnership between the fungus and virus may aggravate the severity of disease. However, the reason for this phenomenon had not been solved in our study, and it will be our next research direction.

### Cross-kingdom transmission between plant viruses and fungi

The earliest report on cross-kingdom transmission of plant viruses by fungi revealed that *Olpidium brassicae* could transmit TMV and cucumber necrosis virus (CNV) [55, 56]*.* Since then, an increasing number of studies have been conducted in this area. A recent study reported that the plant virus CMV can infect *Rhizoctonia cumulus* and can replicate and shuttle between the two different hosts. In addition, hop stunt viroid (HSVd) can also be transmitted between *Fusarium graminea* and plants [55, 56]. Interestingly, plant viruses are also found in invertebrates that are not insect carriers of plant viruses [57], so some plant viruses can be transmitted outside their known hosts. In our study, there was indeed cross-kingdom transmission of ASGV between fungus and plant, as *F. solani* could transmit ASGV to healthy plants, and *F. solani* could also obtain ASGV. Moreover, our study also demonstrated that ASGV living in *F. solani* could be transmitted horizontally in fungi through mycelium fusion and transmitted vertically through asexual spore production. This horizontal and vertical transmission of ASGV in a plant pathogenic fungi may accelerate the spread of the virus, increasing the difficulty of preventing and controlling it. However, these results were obtained under laboratory conditions, and this complex should be further studied under field conditions. Another remaining question is which molecular form the ASGV is transmitted between the plants and fungus. Viruses typically spread as complete virions, composed of a nucleic acid genome encapsulated within a protein capsid. Transmission between plants and fungus occurs through specific mechanisms, such as an insect vector or hyphal fusion [58]. However, the mechanism of virus transmission between plant and pathogenic fungus was not revealed in our study. Therefore, it is necessary to conduct further research to understand the interaction between viruses and pathogenic fungi and to elucidate the molecular mechanism of ASGV movement between apple and *F. solani*.

## Conclusion

This study identifies *F. solani* as a novel vector for the cross-kingdom transmission of ASGV in apple orchards. ASGV carried by *F. solani* retains infectious, leading to reduced plant growth and impaired root development in apple seedlings. The virus spreads horizontally through mycelial fusion and vertically via spores, demonstrating its persistence within fungal populations. Furthermore, inoculation with *F. solani* harboring ASGV exacerbates plant damage, complicating disease management efforts. These findings underscore the role of soil-borne fungi in the epidemiology of apple viruses and call for further research into cross-kingdom transmission mechanisms to improve disease control strategies.

## Supplementary Information

Additional file 1: Supplemental Fig. 1. Morphology of different fungi isolated from apple roots or rhizospheric soil from an old, establish orchard. Supplemental Fig. 2. Virus detection results for the other two ASGV-positive *F. solani* strains and other isolated fungi. Supplemental Fig. 3. Phylogenetic analysis of the complete sequence of ASGV amplified from ASGV^carrying^ *F. solani* isolate and ASGV-infected apple leaves. Supplemental Fig. 4. Status of the leaves of apples cultured in PDA medium.

Additional file 2: Supplemental Table 1. Specific primers for apple viruses/viroids detection by RT‒PCR. Supplemental Table 2. Root indices of plants infected by ASGV^free^ or ASGV^carrying^ spores of *F. solani *[59, 60, 61]

Additional file 3: Supplemental Data 1. Complete sequences of ASGV acquired from ASGV-carrying *F. solani* and ASGV-infected apple leaves in this study, as well as other ASGV and capilloviruses reference sequences acquired from Genbank.

## Data Availability

The viral genome sequence data generated in this study have been deposited in the NCBI GenBank database under the accession numbers PV413210.1 and PV413211.1. All relevant data are provided within the manuscript and its additional files.

## References

[1] FAOSTAT: Crops and livestock products. https://www.fao.org/faostat/en/#data/TCL. Accessed 10 Mar 2025.

[2] Brite EB. The origins of the apple in central Asia. J World Prehist. 2021;34:159–93.

[3] Cornille A, Gladieux P, Smulders MJM, Roldán-Ruiz I, Laurens F, Cam BL, Nersesyan A, Clavel J, Olonova M, FeugeyIvan L, Gabrielyan I, Zhang X, Tenaillon MI, Giraud T. New insight into the history of domesticated apple: secondary contribution of the European wild apple to the genome of cultivated varieties. PLoS Genet. 2012;8: e11002703. 10.1371/journal.pgen.1002703 PMC3349737 22589740

[4] Ji Z, Zhao X, Duan H, Hu T, Wang S, Wang Y, Cao K. Multiplex RT-PCR detection and distribution of four apple viruses in China. Acta Virol. 2013;57:435–41. 24294957 10.4149/av_2013_04_435

[5] Chen S, Ye T, Hao L, Chen H, Wang S, Fan Z, Guo L, Zhou T. Infection of apple by apple stem grooving virus leads to extensive alterations in gene expression patterns but no disease symptoms. PLoS One. 2014;9: e95239. 24736405 10.1371/journal.pone.0095239 PMC3988175

[6] Malandraki I, Beris D, Isaioglou I, Olmos A, Varveri C, Vassilakos N. Simultaneous detection of three pome fruit tree viruses by one-step multiplex quantitative RT-PCR. PLoS One. 2017;12: e0180877. 28749955 10.1371/journal.pone.0180877 PMC5547701

[7] Shokri S, Shujaei K, Gibbs AJ, Hajizadeh M. Evolution and biogeography of apple stem grooving virus. Virol J. 2023;20: 105. 37237285 10.1186/s12985-023-02075-2 PMC10223889

[8] Yoon JY, Joa JH, Choi KS, Do KS, Lim HC, Chung BN. Genetic diversity of a natural population of apple stem pitting virus isolated from apple in Korea. Plant Pathol J. 2014;30:195–9. 25289003 10.5423/PPJ.NT.02.2014.0015 PMC4174845

[9] Li Y, Deng C, Bian Y, Zhao X, Zhou Q. Characterization of apple stem grooving virus and apple chlorotic leaf spot virus identified in a crab apple tree. Arch Virol. 2016;162:1093–7. 27990565 10.1007/s00705-016-3183-2

[10] Pina A, Errea P. A review of new advances in mechanism of graft compatibility–incompatibility. Sci Hortic. 2005;106:1–11.

[11] Brakta A, Thakur PD, Handa A. First report of apple top working disease caused by viruses (apple stem grooving virus, apple chlorotic leaf spot virus, and apple stem pitting virus) in apple in India. Plant Dis. 2013;97:1001. 30722528 10.1094/PDIS-11-12-1082-PDN

[12] Massart S, Olmos A, Jijakli H, Candresse T. Current impact and future directions of high throughput sequencing in plant virus diagnostics. Virus Res. 2014;188:90–6. 24717426 10.1016/j.virusres.2014.03.029

[13] Chen J, Tang H, Li L, Qin S, Wang G, Hong N. Effects of virus infection on plant growth, root development and phytohormone levels in in vitro-cultured pear plants. Plant Cell. 2017;131:359–68.

[14] Cembali T, Folwell RJ, Wandschneider P, Eastwell KC, Howell WE. Economic implications of a virus prevention program in deciduous tree fruits in the US. Crop Prot. 2003;22:1149–56.

[15] Kishigami R, Yamagishi N, Ito T, Yoshikawa N. Detection of apple latent spherical virus in seeds and seedlings from infected apple trees by reverse transcription quantitative PCR and deep sequencing: evidence for lack of transmission of the virus to most progeny seedlings. J Gen Plant Pathol. 2014;80:490–8.

[16] Pethybridge SJ, Wilson CR, Hay FS, Leggett GW, Sherriff LJ. Mechanical transmission of apple mosaic virus in Australian hop (*Humulus lupulus*) gardens. Ann Appl Biol. 2002;141:77–85.

[17] Jiao J, Kong K, Han J, Song S, Bai T, Song C, Wang M, Yan Z, Zhang H, Zhang R, Feng J, Zheng X. Field detection of multiple RNA viruses/viroids in apple using a CRISPR/Cas12a-based visual assay. Plant Biotechnol J. 2021;19:394–405. 32886837 10.1111/pbi.13474 PMC7868969

[18] Zhao L, Wang M, Cui Z, Chen L, Volk GM, Wang Q. Combining thermotherapy with cryotherapy for efficient eradication of apple stem grooving virus from infected in-vitro-cultured apple shoots. Plant Dis. 2018;102:1574–80. 30673422 10.1094/PDIS-11-17-1753-RE

[19] Heitefuss R. Virus and virus-like diseases of pome and stone fruits. J Phytopathol. 2012;160:508–508.

[20] Grimová L, Winkowska L, Zíka L, Rysanek P. Distribution of viruses in old commercial and abandoned orchards and wild apple trees. J Plant Pathol. 2016;98:549–54.

[21] Schröder M. Soil transmission studies with four pome fruit viruses. J Kulturpfl. 2021;73:72–82.

[22] Yang J, Liu P, Zhong K, Ge T, Chen L, Hu H, Zhang T, Zhang H, Guo J, Sun B, Chen J. Advances in understanding the soil-borne viruses of wheat: from the laboratory bench to strategies for disease control in the field. Phytopathol Res. 2022;4: 27.

[23] Singh S, Awasthi LP, Jangre A. Chapter 24 - Transmission of plant viruses in fields through various vectors. Appl Plant Virol*.* 2020;313–334.

[24] Andika IB, Wei S, Cao C, Salaipeth L, Sun L. Phytopathogenic fungus hosts a plant virus: a naturally occurring cross-kingdom viral infection. Proc Natl Acad Sci U S A. 2017. 10.1073/pnas.1714916114. 29087346 10.1073/pnas.1714916114 PMC5699089

[25] Peiffer JA, Spor A, Koren O, Jin Z, Tringe SG, Dangl JL, Buckler ES, Ley RE. Diversity and heritability of the maize rhizosphere microbiome under field conditions. Proc Natl Acad Sci U S A. 2013;110:6548–53. 23576752 10.1073/pnas.1302837110 PMC3631645

[26] Rodriguez RJ, White JF Jr, Arnold AE, Redman RS. Fungal endophytes: diversity and functional roles. New Phytol. 2009;182(2):314–30. 19236579 10.1111/j.1469-8137.2009.02773.x

[27] Guo Z, Qin Y, Lv J, Wang X, Ye T, Dong X, Du N, Zhang T, Piao F, Dong H, Shen S. High red/far-red ratio promotes root colonization of *Serratia plymuthica* A21–4 in tomato by root exudates-stimulated chemotaxis and biofilm formation. Plant Physiol Biochem. 2024;206: 108245. 38064903 10.1016/j.plaphy.2023.108245

[28] Ghabrial SA, Castón JR, Jiang D, Nibert ML, Suzuki N. 50-plus years of fungal viruses. Virology. 2015;479–480:356–68. 25771805 10.1016/j.virol.2015.02.034

[29] Kanyuka K, Ward E, Adams MJ. *Polymyxa graminis* and the cereal viruses it transmits: a research challenge. Mol Plant Pathol. 2003;4:393–406. 20569399 10.1046/j.1364-3703.2003.00177.x

[30] Wei S, Bian R, Andika IB, Niu E, Sun L. Symptomatic plant viroid infections in phytopathogenic fungi. PNAS. 2019;116:13042–50. 31182602 10.1073/pnas.1900762116 PMC6600922

[31] Lee Marzano SY, Nelson BD, Ajayi-Oyetunde O, Bradley CA, Hughes TJ, Hartman GL, Eastburn DM, Domier LL. Identification of diverse mycoviruses through metatranscriptomics characterization of the viromes of five major fungal plant pathogens. J Virol. 2016;90:6846–63. 27194764 10.1128/JVI.00357-16 PMC4944287

[32] Dolja VV, Koonin EV. Metagenomics reshapes the concepts of RNA virus evolution by revealing extensive horizontal virus transfer. Virus Res. 2018;244:36–52. 29103997 10.1016/j.virusres.2017.10.020 PMC5801114

[33] Shim HK, Hwang KH, Shim CK, Son SW, Kim D, Choi YM, Chung Y, Kim DH, Jee HJ, Lee SC. The pear black necrotic leaf spot disease virus transmitted by *Talaromyces flavus* displays pathogenicity similar to apple stem grooving virus strains. Plant Pathol J. 2006;22:255–9.

[34] Guo J, Yin J, Hu H, Zhang T, Ye Z, Yang J, Liu H, Chen J, Liu J. Molecular characterization of a novel *benyvirus* infecting wheat in China. Arch Virol. 2023;168: 284. 37930401 10.1007/s00705-023-05912-5

[35] Dai R, Yang S, Pang T, Tian M, Wang H, Zhang D, Wu Y, Kondo H, Andika IB, Kang Z, Sun L. Identification of a negative-strand RNA virus with natural plant and fungal hosts. Proc Natl Acad Sci U S A. 2024. 10.1073/pnas.2319582121. 38483998 10.1073/pnas.2319582121 PMC10962957

[36] Cheng C, Zhang M, Niu Y, Zhang M, Geng Y, Deng H. Comparison of fungal genera isolated from cucumber plants and rhizosphere soil by using carious cultural media. J Fungi. 2023;9(9): 934. 10.3390/jof9090934 PMC10532442 37755042

[37] Gardes M, Bruns TD. Its primers with enhanced specificity for basidiomycetes-application to the identification of *mycorrhizae* and rusts. Mol Ecol. 2008;2:113–8. 10.1111/j.1365-294x.1993.tb00005.x 8180733

[38] Vilgalys R, Hester M. Rapid genetic identification and mapping of enzymatically amplified ribosomal DNA from several *Cryptococcus* species. J Bacteriol. 1990;172:4238–46. 2376561 10.1128/jb.172.8.4238-4246.1990 PMC213247

[39] Hu G, Dong Y, Zhang Z, Fan X, Ren F. Elimination of apple necrosis mosaic virus from potted apple plants by thermotherapy combined with shoot-tip grafting. Sci Hortic. 2019;252:310–5.

[40] Dhir S, Walia Y, Zaidi AA, Hallan V. A simplified strategy for studying the etiology of viral diseases: Apple stem grooving virus as a case study. J Virol Methods. 2015;213:106–10. 25486082 10.1016/j.jviromet.2014.11.017

[41] Kumar S, Stecher G, Suleski M, Sanderford M, Sharma S, Tamura K, Battistuzzi FU. MEGA12: molecular evolutionary genetic analysis version 12 for adaptive and green computing. Mol Biol Evol. 2024;41:1–9. 10.1093/molbev/msae263 PMC11683415 39708372

[42] Bouma TJ, Nielsen KL, Koutstaal B. Sample preparation and scanning protocol for computerised analysis of root length and diameter. Plant Soil. 2000;218:185–96.

[43] Sun J, Yang L, Yang X, Wei J, Li L, Guo E, Kong Y. Using spectral reflectance to estimate the leaf chlorophyll content of maize inoculated with arbuscular mycorrhizal fungi under water stress. Front Plant Sci. 2021;12: 646173. 34122471 10.3389/fpls.2021.646173 PMC8193845

[44] Ghabrial SA, Suzuki N. Viruses of plant pathogenic fungi. Annu Rev Phytopathol. 2009;47:353–84. 19400634 10.1146/annurev-phyto-080508-081932

[45] Domingo E, Sheldon J, Perales C. Viral quasispecies evolution. Microbiol Mol Biol Rev. 2012;76:159–216. 22688811 10.1128/MMBR.05023-11 PMC3372249

[46] Parrish CR, Holmes EC, Morens DM, Park E, Burke DS, Calisher CH, Laughlin CA, Saif LJ, Daszak P. Cross-species virus transmission and the emergence of new epidemic diseases. Microbiol Mol Biol. 2008;72:457–70. 10.1128/MMBR.00004-08 PMC2546865 18772285

[47] Ajmal M, Hussain A, Ali A, Chen H, Lin H. Strategies for controlling the sporulation in *Fusarium* spp. J Fungi. 2022;9: 10. 10.3390/jof9010010 PMC9861637 36675831

[48] Luo X, Li J, Dong J, Sui A, Sheng M, Yang X. First report of *Fusarium solani* causing root rot on *Coptis chinensis* in southwestern China. Plant Dis. 2014;98:1273. 30699657 10.1094/PDIS-02-14-0164-PDN

[49] Yang M, Jiao J, Liu Y, Li M, Xia Y, Hou F, Huang C, Zhang H, Wang M, Shi J, Wan R, Zhang K, Hao P, Bai T, Song C, Feng J, Zheng X. Genome-wide investigation of defensin genes in apple (*Malus×domestica* Borkh.) and in vivo analyses show that MdDEF25 confers resistance to *Fusarium solani*. J Integr Agric. 2025;24:161–75.

[50] Xie J, Jiang D. New insights into *mycoviruses* and exploration for the biological control of crop fungal diseases. Annu Rev Phytopathol. 2014;52:45–68. 25001452 10.1146/annurev-phyto-102313-050222

[51] Janda M, Ahlquist P. RNA-dependent replication, transcription, and persistence of brome mosaic virus RNA replicons in *S. cerevisiae*. Cell. 1993;72:961–70. 8458084 10.1016/0092-8674(93)90584-d

[52] Nagy PD. Yeast as a model host to explore plant virus-host interactions. Annu Rev Phytopathol. 2008;46:217–42. 18422427 10.1146/annurev.phyto.121407.093958

[53] Mascia T, Nigro F, Abdallah A, Ferrara M, De Stradis A, Faedda R, Palukaitis P, Gallitelli D. Gene silencing and gene expression in phytopathogenic fungi using a plant virus vector. Proc Natl Acad Sci U S A. 2014;111:4291–6. 24594602 10.1073/pnas.1315668111 PMC3964105

[54] Koonin EV, Dolja VV, Krupovic M. Origins and evolution of viruses of eukaryotes: the ultimate modularity. Virology. 2015;479–480:2–25. 25771806 10.1016/j.virol.2015.02.039 PMC5898234

[55] Dias F, Dias H. Transmission of cucumber necrosis virus by *Olpidium cucurbitacearum* Barr & Dias. Virology. 1970;40:828–39. 5450390 10.1016/0042-6822(70)90128-5

[56] Temmink JHM, Campbell RN, Smith PR. Specificity and site of *in vitro* acquisition of tobacco necrosis virus by zoospoores of *Olpidium brassicae*. J Gen Virol. 1970;9:201–3.

[57] Shi M, Lin X, Tian J, Chen L, Chen X, Li C, Qin X, Li J, Cao J, Eden JS, Buchmann J, Wang W, Xu J, Holmes EC, Zhang Y. Redefining the invertebrate RNA virosphere. Nature. 2016;540:539–43. 27880757 10.1038/nature20167

[58] Nowara D, Gay A, Lacomme C, Shaw J, Ridout C, Douchkov D, Hensel G, Kumlehn J, Schweizer P. HIGS: host-induced gene silencing in the obligate biotrophic fungal pathogen *Blumeria graminis*. Plant Cell. 2010;22:3130–41. 20884801 10.1105/tpc.110.077040 PMC2965548

[59] Noda H, Yamagishi N, Yaegashi H, Xing F, Xie J, Li S, Zhou T, Ito T, Yoshikawa N. Apple necrotic mosaic virus, a novel *ilarvirus* from mosaic-diseased apple trees in Japan and China. J Gen Plant Pathol. 2017;83.

[60] Komorowska B, Malinowski T, Michalczuk L. Evaluation of several RT-PCR primer pairs for the detection of apple stem pitting virus. J Virol Methods. 2010;168:242–7. 20447421 10.1016/j.jviromet.2010.04.024

[61] Sipahioglu HM, Usta M, Ocak M. Use of dried high-phenolic laden host leaves for virus and viroid preservation and detection by PCR methods. J Virol Methods. 2006;137:120–4. 16879877 10.1016/j.jviromet.2006.06.009

