# Supplementary material for: Transmission of apple stem grooving virus (Capillovirus mali) to apple from the soil-borne fungus Fusarium solani
Source: BMC Plant Biol. 2025 Sep 29;25:1226. doi: 10.1186/s12870-025-07188-0 (PMC12477809; doi:10.1186/s12870-025-07188-0)
Supplement: Supplementary file 1 — Additional file 1: Supplemental Fig. 1. Morphology of different fungi isolated from apple roots or rhizospheric soil from an old, establish orchard. Supplemental Fig. 2. Virus detection results for the other two ASGV-positive F. solani strains and other isolated fungi. Supplemental Fig. 3. Phylogenetic analysis of the complete sequence of ASGV amplified from ASGVcarrying F. solani isolate and ASGV-infected apple leaves. Supplemental Fig. 4. Status of the leaves of apples cultured in PDA medium. [file 12870_2025_7188_MOESM1_ESM.docx]

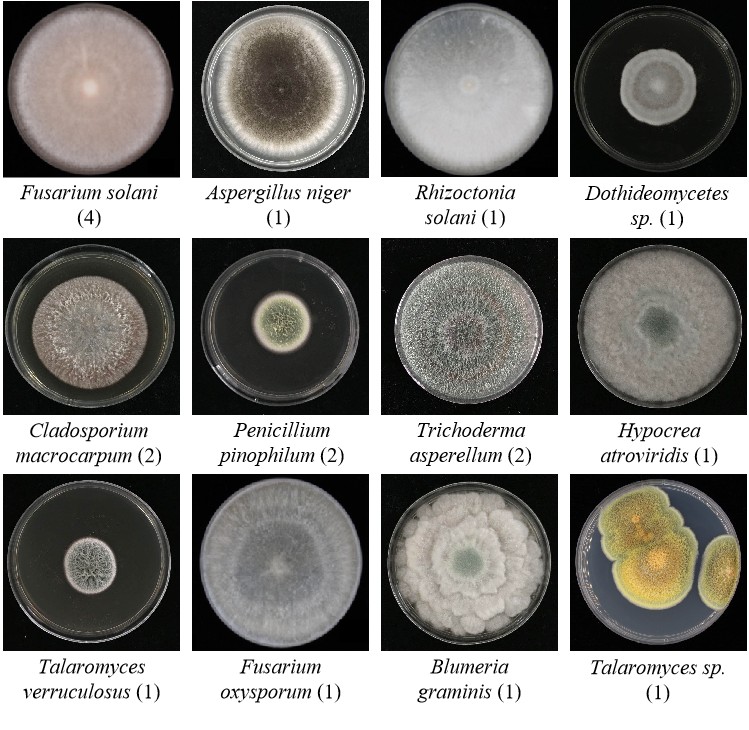


**Supplemental Figure 1.** Morphology of different fungi isolated from apple roots or rhizospheric soil from an old, establish orchard. The identification of each strain was determined based on the sequencing results of the ITS region in conjunction with the morphological analysis. Numbers in parentheses means the number of strains isolated from each fungus species.


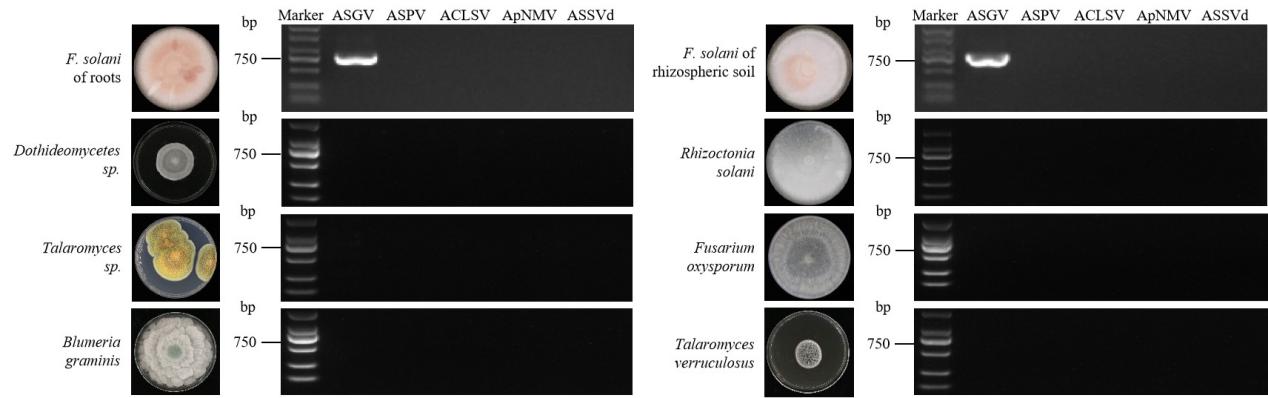


**Supplemental Figure 2.** Virus detection results for the other two ASGV-positive *F. solani* strains and other isolated fungi. Only ASGV was detected among the five tested viruses from the two *F. solani strains* isolated from apple roots or rhizospheric soil, while no apple viruses were detected in any of the other fungi.


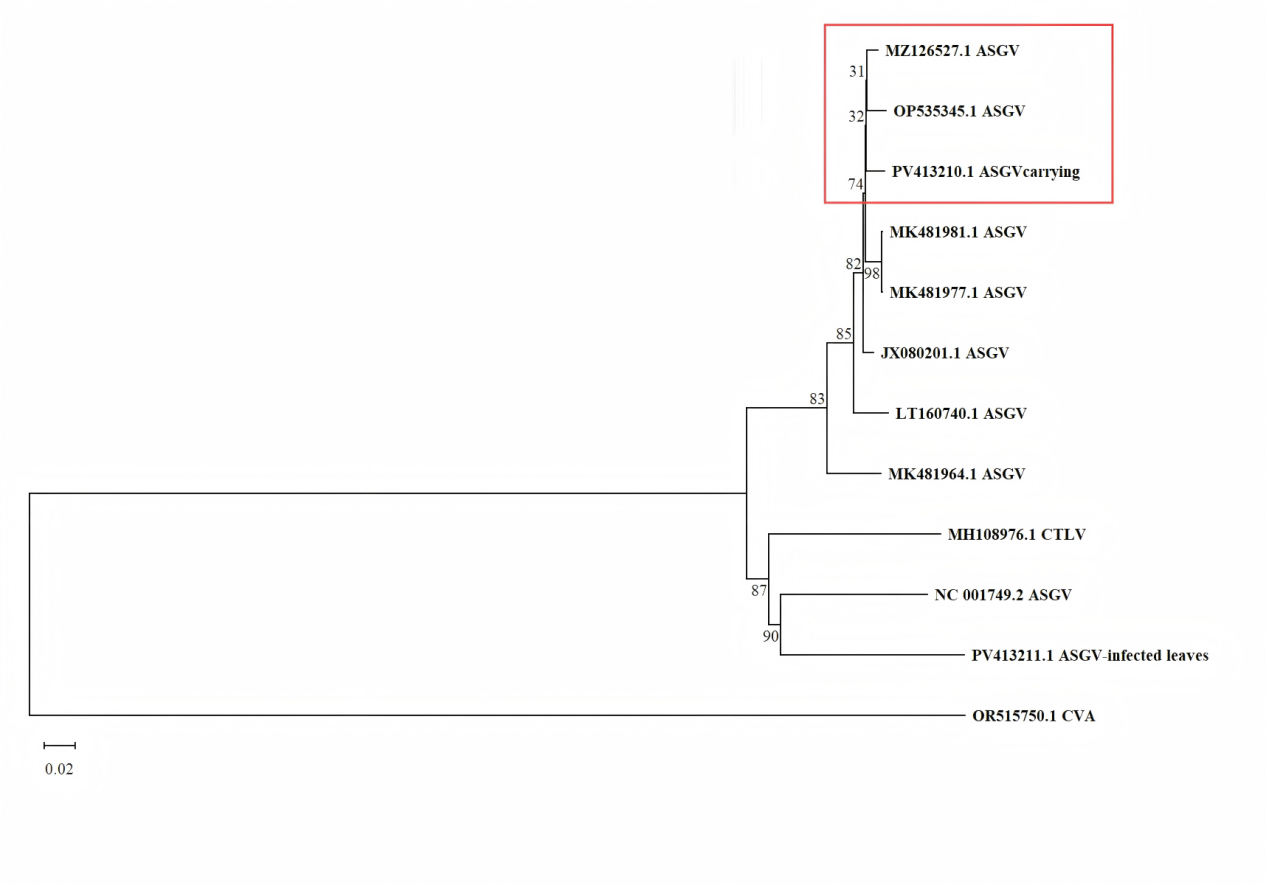


**Supplemental Figure 3.** Neighbour-joining phylogenetic tree. Phylogenetic analysis of the complete sequences of ASGV amplified from ASGV^carrying^ *F. solani* isolate and ASGV-infected apple leaves. Complete sequences of ASGV, citrus tatter leaf virus (CTLV), and cherry virus A (CVA) were obtained from the NCBI database. The numbers above the branches are support values obtained from 1,000 bootstrap replicates.


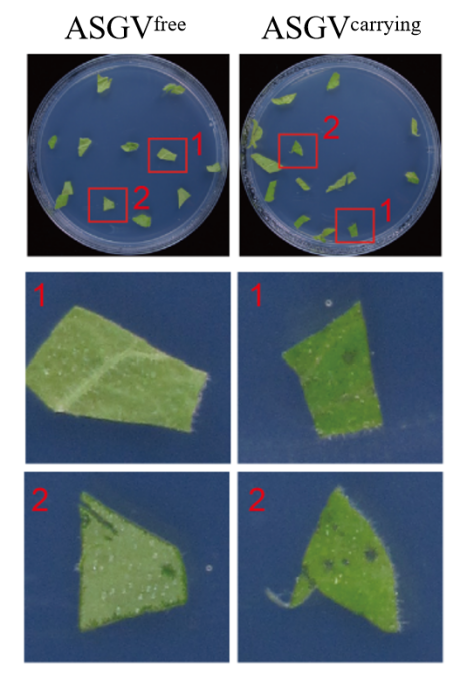


**Supplemental Figure 4.** Status of the leaves of apples cultured in PDA medium. Apple plants were inoculated with ASGV^free^ and ASGV^carrying^ isolates of *F. solani* by soaking roots in a spore suspension, after 4 weeks, leaves of the inoculated plants were cultured on PDA medium. No fungus grew around the leaves, indicating that the upper leaves of the apple plants were not contaminated by the mycelia of *F. solani*.
