# Supplementary material for: Transmission of apple stem grooving virus (Capillovirus mali) to apple from the soil-borne fungus Fusarium solani
Source: BMC Plant Biol. 2025 Sep 29;25:1226. doi: 10.1186/s12870-025-07188-0 (PMC12477809; doi:10.1186/s12870-025-07188-0)
Supplement: Supplementary file 2 — Additional file 2: Supplemental Table 1. Specific primers for apple viruses/viroids detection by RT‒PCR. Supplemental Table 2. Root indices of plants infected by ASGVfree or ASGVcarrying spores of F. solani [59, 60, 61] [file 12870_2025_7188_MOESM2_ESM.docx]

| **Supplemental Table 1. Specific primers for apple viruses/viroids detection by RT‒PCR** | | | | |
| --- | --- | --- | --- | --- |
| **Primer name** | **Viruses** | **Sequence (5'-3')** | **Size (bp)** | **References** |
| ApNMV-CP+1 | ApNMV | cttgcgtgcaatcgatatgg | 685 | Noda et al  [59] |
| ApNMV-CP-1 |  | tcatctcaacctagacatcc |  |  |
| ASGV-F | ASGV | ATGAGTTTGGAAGACGTGCTTC | 733 | Redesigned according to Shim et al [33] |
| ASGV-R |  | CTAACCCTCCAGTTCCAAGTTA |  |  |
| ASPF1CP | ASPV | GGGTGTACTTTGAGGCAGTATT | 257 | Komorowska et al [60] |
| ASPR3CP |  | AGCGGATGCGGTACATCTGTAT |  |  |
| C-F1 | ACLSV | CAGACCYCTTCATGGAAAGACAG | 725 | Hu et al  [39] |
| C-R1 |  | GTAGTAAAATATTTAAAAGTCTACAGG |  |  |
| AS1 | ASSVd | CCGGCCTTCGTCGACGACGA | 330 | Sipahioglu et al [61] |
| AS3 |  | TGAGAAAGGAGCTGCCAGCAC |  |  |

| **Supplemental Table 2. Root indices of plants infected by ASGV^free^ or ASGV^carrying^ spores of *F. solani*** | | | | | | |
| --- | --- | --- | --- | --- | --- | --- |
| **Treatment** | **Root architecture parameters** | | | | | |
|  | **Surface Area**  **(cm^2^)** | **Root Length**  **(cm)** | **Root volume**  **(cm^3^)** | **Number of root tips** | **Forks** | **Root diameter**  **(mm)** |
| Mock | 110.97±6.27 a | 299.89±6.56a | 3.83±0.082 a | 813±19 a | 2969±225 a | 1.85±0.055 a |
| ASGV^free^ | 84.82±3.63 b | 202.13±9.91b | 3.22±0.122 b | 682±22 b | 1951±100 b | 1.66±0.029 b |
| ASGV^carrying^ | 77.86±3.99 c | 151.30±6.68 c | 2.73±0.067 c | 472±11 c | 1421±41 c | 1.36±0.016 c |

Note: The data are presented as the means ± SEs. Different letters (a, b, c) within the same comparison group indicate significant differences assessed by three-way ANOVA at *P* < 0.05. n = 10 for each treatment.
