# Supplementary material for: Transmission of apple stem grooving virus (Capillovirus mali) to apple from the soil-borne fungus Fusarium solani
Source: BMC Plant Biol. 2025 Sep 29;25:1226. doi: 10.1186/s12870-025-07188-0 (PMC12477809; doi:10.1186/s12870-025-07188-0)
Supplement: Supplementary file 3 — Additional file 3: Supplemental Data 1. Complete sequences of ASGV acquired from ASGV-carrying F. solani and ASGV-infected apple leaves in this study, as well as other ASGV and capilloviruses reference sequences acquired from Genbank. [file 12870_2025_7188_MOESM3_ESM.docx]

**Supplemental Data 1.** Complete sequences of ASGV acquired from ASGV-carrying *F. solani* and ASGV-infected apple leaves in this study, as well as other ASGV and capilloviruses reference sequences acquired from Genbank.

1. **Complete sequence of ASGV acquired from *F. solani* carrying ASGV. Accession number: PV413210.1 (6447 bp).**

CTTTACGTTAATGGCTTTCACTTACAGAAATCCCCTCGAAATTGCAATCAACAAGCTTCCAAGTAAGCAGTCTGATCAACTGCTTTCTTTAACCACAGACGAGATTGAAAAGACCTTAGAAGTGACTAATCGCTTCTTCTCTTTTTCTATCACTCCAGAAGATCAAGAATTGTTGACCAAGCATGGTCTAACACTTGCACCCATTGGATTCAAGTCACATTCCCATCCTATATCCAAAATGATAGAAAATCACCTTCTGTACATATGCATTCCAAGTCTTTTATCTTCTTTTAGATCCGTAGCTTTTTTTTCGCTTAGAGAAAATAAAGTCAATAATTTTCTTAAGATGCACTCAGTTTTTTCACATGGAAAAATTAAGTCTCTAGGCATGTACAACGCCATCATTGATGGAAAAGATAAATACAGGTATGGCGAAGTTTCTTTTTCTTCCTTTAGAGATAGGGTGATTGGTCTCAGGGATCAGTGCCTCATGCGTAATAAATTTCCAAAAGTTCTCTTTCTCCACGATGAACTTCATTTCCTAAGCCCTTTCGACGTTGCTTTTCTATTTGAGACAATCCCAGAAATTGATAGAGTCATAGCAACTACTGTCTTTCCGATTGAACTGCTTTTTGGGGACAAGGTTTCAAAGGAGCCCAGAGTTTACACCTACAAGGTCCATGGTTCTTCCTTTTCATTCTATCCAGATGGTGTAGCCTCTGAGTGCTATGAGCAAAATCTTGCAAACTCCAAATGGCCGTTCACTTGTAGTGGAATTCAGTGGGCTAACAGGAAAATAAGGGTTACTAAGTTGCAGAGCCTCTTTGCTCATCACGTTTTTTCATTTGATAGAGGTAGGGCTTGCAACGAGTTCAACCACTTTGACAAGCCGAGTTGTCTCCTTGCAGCCGAAATGCGCCTTTTGACCAAAAGGTTTGATAAGGCAGTTATTAATAGGAGTACGGTTTCTTCTTTGAGCACTTATATGGCTTGTCTCAAGACCGCCAATGCAGCTTCTGCTGTCGCCAAGCTAAGGCAACTAGAGAAAAGGGACCTCTATCCGGATGAACTGAACTTCGTTTATTCCTTTGGGGAGCATTTCAAAAATTTTGGAATGAGGGATGATTTTGATGTGTCAGTCCTGCAATGGGTGAAGGACAAATTCTGTCAAGTTATGCCTCACTTCATTGCTGCCAGTTTCTTTGAGCCAACTGAGTTCCATTTGAATATGCGAAAATTGTTGAATGACCTGGCCACTAAAGGGATAGAGGTTCCACTTTCAGTAGTGGTTTTAGATAAGGTAAATTTTATAGAGACCAGATTTCATGCCAGAATGTTCGAAATTGCCGAGGCTATTGGAGTCAGTTTAAGTCTACTTGGAAAAAGGTTTGACTATGAAGCTGAAAGTGAAGAATATTTTTCAGAAAATGGCTATCTTTTCATGCCTTCAAGGACAAATCCAAGTAGAAACTGGATCTTAAACTCTGGGTCTGTCAAAATTGATTTCCAGAAGTTGCAGAGGGCAAGGAGGTTCAGATTGAGAAGAGATTTCGTTGATTTGATTGCTAGAAGGGAAACCCCTCGAAAGCAACTATTCCTCGACTCAGCTCAAACTCTCCAATCAACGTCTAAAAGTGTGGAGGAGAACATGGACAGTGGGGTCAAATCCGATGAAAGCAGTAAAAAAATACAGTCACTCGAGGTGCCCTGTGTCCCGATGTCCACTGAAGATGGGCAAGGCTTTGAAGGTTCAATTCCAATTGACTTGATAAATTCCTTCGAGCCAGAAGTAATCAGACTTCCGAAAAGGAAGAGGAAGAATGATTGTGTTTTTAAAGCCATCTCTGCATACCTTGGCATTGATGTTCAGGACCTCTTGAACTTTTTGGTGAATGAAGACATATCCGATGAGTTGCTTGAATGCATAGATGAAGATAAAGGCCTGTCGCACGAGATGATAGAGGAAGTTCTGGTGACCAAAGGGATTTCTATGGTCTACACTTCAGATTTCAAAGAGATGGCAGTCCTCAACAGGAAATATGGAGTTAATGGAAAAATGTACTGCACAATCAGGGGGAATCATTGTGAGCTGAGTTCAAAGGAATGCTTCATCAGATTGCTGAAGGAGGGTGGTGAAGCACAAATGTCCAATGAGAATTTGAATGCTGACTCCTTGTTTGATCTTGGAAAGTTCGTGCACAACAGAGAAAGGGCCACAAAATTGGCAAAATCGATGGCTAGGGGCACAACCGGTCTTTTGACTGAATTTGATCCAGAATTTTGTAAAGGTATGGTCACACTTTCGGAAATGTTTCCGGAGAACTTCTCCTCTGTTGTCGGACTGAGGTTGGGCTTCGCTGGTTCCGGAAAAACCCATAAAGTTCTTCAATGGATCAACTACACACCAAGCGTCAAAAGAATGTTTATTAGTCCAAGGAGAATGCTGGCCGACGAGGTTGAAATTCAATTAAGAGGGACATCTTGTCAAGTTCATACTTGGGAAACAGCTCTTAAAAAGATTGATGGAACTTTCATGGAGGTCTTTGTGGATGAAATTGGCCTTTACCCACCTGGATATCTCACTCTTCTACAAATGTGTGCTTTCCGAAGGATCGTTAAGGGGCAGAGTGAAAAGTTTTTAAGAGGAAAGCTTGCTGAACTTTCGAAGACCTGTTTGAATGTAAGGTGTTTTGGTGACCCATTGCAAATGAGGTATTATTCTGCAGAGGATACAAACCTTCTAGACAAGACACATGAGCTTGATCTAATGGTCAGAACCATCAAACACAAGTACCTTCTTCAAGGGTACAGGTTCGGCCACTGGTTCCAAGAGCTGATAAACATGCCAACTAGAGTGGACGATTCAAAATTTTCAAGGAAATTTTTTGCGGACATTTCCAGTGTCAAAACCGAAGATTATGGACTCATACTTGTCGCCAAGAGGGAGGACAAGGGGACGTTTGCCGGGAGAATACCTGTGGCTACGGTCAGCGAATCACAGGGAATGACCATAAGCAAGAGGGTGCTAATATGCTTAGACCAAAATCTGTTTGCCGGGGGAGCCAATGCCGCCATAGTTGCGATCACTAGATCGAAAGTTGGGTTTGATTTTATTCTCAAAGGGAATTCACTAAAAGAGGTGCAGAGGATGGCCGAAAAAACTTTGTGGCAATTCGTACTTGAAGGGAAGACCATACCGATGGAAAGAATTGTCAATATGAACCCCGGAGCCAGCTTTTATGAGAGCCCCTTAGACGTTGGTAACACGTCCATTCAGGACAAAGCCTCACATGACTTGTTCATAATGCCTTTTATCAATTTAGCTGAAGAAGAAGTTGACCCTGAGGAAATCGTCGGAGATGTTATTGAACCAGTTGAGTGGTTCAAATGCCATATACCAGTTTTTGATACAGACCCAATGCTTGCAGCGATATTTGACAAAGTGGCAGCAAAGGAAAAGAGAGAATTCCAGTCTATACTTGGGCTTTCAAATCAATTTCTTGACATGGAAAAAAATGGATGCAAAATCGATATTTTGCCTTTTGCTAGACACAATGTTTTCCCTCACCATCAAGCTTCAGATGACGTCACCTTTTGGGCAGGTGTTCAGAAGAGGATAAGAAAGTCAAATTGGAGACGGGAAAAGTCGAAATTTGAGGAGTTTGAAGCTCAGGGGAAGGAATTGCTCAGTGAATTCATATCAATGTTGCCCTTTGAATTCAAGGTGAACATCAAGGACATTGAAGAAGGTGAAAAAAGCTTTCTTGAAAAGAGAAAGTTAAAGTCTGAAAAGATGTGGGCCAACCACTCAGAAAGATCTGACATTGACTGGAAACTTGACCATGCCTTTCTGTTTATGAAATCTCAATATTGCACAAAGGAAGGCAAAATGTTCACTGAGGCAAAAGCTGGTCAAACCTTAGCATGTTTCCAACACATTGTTCTGTTCAGGTTTGGTCCCATGCTTCGAGCAATTGAAAGGGCTTTTCTTAGAAGTTGTGGAGAGTCATACTACATACATTCAGGAAAAAACTTTTTTTGCTTGGACAGCTTTGTGACAAAAAACGCAGAAGTCTTCGATGGGTTTTCTATTGAATCTGACTACACGGCTTTTGACTCATCTCAGGACCACGTGATTTTGGCCTTTGAAATGGCACTTTTGCAGTACCTAGGGGTGTCCAAAGAGTTTCAGTTGGATTATTTGAGATTGAAATTGACCCTTGGCTGCAGAGTTGGATCTCTAGCCATTATGAGGTTCACTGGGGAATTCTGCACCTTTTTGTTCAATACATTTGCAAACATGCTTTTCACCCAGTTGAAATACAAAATTGACCCGAGGAAGCATAGGATTCTATTTGCTGGAGATGACATGTGCTCTCTAAGTTCTCTCAAAAGAAGAAAAGGGGAAAAGGCAACAAGACTGTTGAAGAGCTTTTCTTTAACTGCCGTGGAAGAGGTACGGAAGTTTCCAATGTTCTGCGGGTGGTACTTGAGCCCCTATGGGATTATCAAGTCTCCAAAGTTGTTGTGGGCTAGGATCAAAATGATGAGTGAAAGACAACTTTTAAATAAATGTGTGGACAACTATCTGTTTGAAGCTATATTTGCCTACAGATTAGGTGAGAGGCTTTACACAATTTTGAAAGAAGAGGACTTTGAGTATCATTACCTTGTTATAAGGTTTTTTGTCAAAAATTCTAAGTTGCTGACAGGGTTGAGCAAAAGCCTCATTTTCGAAATTGGTGAAGGGATTGGGTCAGAATGGCAATCGTCAATGTCAACCATTTCCTCAAGGAAGTCGAGTCAACACATCTCAAAATTGATGCCATCTCTTCCTCGGAGTTGTACAAAGATGCCACTTTTTTTAAACCAGATGTGCTTAACTGCATCAAGCGATTTGAATCAAATGTCAAGGTGTCTTCAAGGTCAGGTGATGGGCTCGTGTTGTCTGATTTCAAACTACTTGATGACACAGAGATCGACTCCATTAGAAAGAAGAGCAACAAATACAAGTATCTGCATTATGGAGTCATTCTGGTCGGGATTAAGGCTATGCTACCGAACTTTAGGGGAATGGAGGGAAGGGTCATTGTGTATGACGGTGCATGCTTGGACCCAGAAAGAGGTCACATTTGTTCCTACTTATTCAAATTTGAGTCCGACTGTTGTTACTTTGGGCTTAGACCGGAACATTGCCTATCCACAACCGATGCAAATTTGGCTAAAAGGTTCAGATTTAGAGTGGATTTTGATTGTCCACAATACGAGCAGGACACAGAGCTGTTTGCTCTCGACATTGGGGTTGCATACAGGTGTGTCAATTCAGCCAGATTCTTGGAAACTAAGACTGGTGATTCAGGATGGGCTTCACAGGCAATCAGCGGCTGTGAGGCACTTAAATTCAATGAAGAAATCAAGATGGCAATCCTGGATCACAAATCACCGCTGTTTCTGGAAGAAGGTGCACCAAACGTGCACATTGAAAAGAGAATGTTTAGGGGTGACAAAGTCAGAAGGTCACGCTCTATCTCTGCAAAAAGGGGGCCAAACTCAAGGCTACAGGAAAAGAGAGGATTTAGGTCCCTCTCAGCTAGAATCGAAAGATTTGGAGAAAATGAGTTTGGAAGACGTGCTTCAACAAGCGAGACGCCACCGGGTAGGAGTGTATCTGTGGAAGACACACATAGACCCGGCAAAGGAACTTCTGACGGTTCCTCCCCCTGAAGCATTCAAGGAAGGTGAAAGCTTTGAAGGCAGAGAGCTTTACATTCTTCTCTGCAACCATTATTGTAAATATTTATTTGGTAATATTGCTGTTTTCGGGTCATCTGACAAGACCCAGTTTCCCGCTGTTGGATTTGATACCCCTCCGGTTCATTACAATCTAACAACGACCCCGAAAGAAGGGGAGACTGAAGAGCAAAGGAAGGCCAGAGAAGGTTCGTCTGGCGAAAAAACAAAAATTTGGAGAATCGACCTGTCAAATGTTGTACCCGAATTGAAAACTTTTGCTGCCACTTCCAGGCAGAACTCTTTGAACGAATGTACGTTCAGAAAGCTTTGTGAGCCATTTGCTGATTTGGCTGGGGAATTTCTTCATGAAAGATGGTCAAGGGGACTTGCCACCAACATTTATAAGAAATGGCCCAAAGCTTTTGAAAAGAGCCCTTGGGTGGCTTTTGATTTTGCCACCGGTCTTAAAATGAACCGTCTAACGCCTGATGAAAAACAGGTAATAGACAGAATGACTAAGAGGCTTTTTCGTACTGAAGGACAAAAAGGGGTTTTCGAGGCGGGTTCGGAGAGTAATCTGGAACTGGAGGGTTAGAAGTCGTGTGAAATTCCGCAAACTTGGTCGCGGTCTTGCAGGTTGACATGCCTGCCTTTATACTTAAATAAAGGGTTCCCCCGGTTTTCTGCGCATTCCGGGTTAGAGCCCATGTATAG

1. **Complete sequence of ASGV acquired from ASGV-infected apple leaves. Accession number: PV413210.1 (6508 bp).**

CGCAGAATTTTAACAGCGCCTAATTTCCGCGCTTTACACACATGGCTTTCACTTACAGAAACCCCCTCGAAATTGCAATCAACAAACTTCCTAGTAAGCAGTCTGATCAACTGCTTTCCCTGACCACCGACGAGATTGAAAAGACCTTAGAAGTGACCAACCGCTTCTTCTCTTTTTCGATCACACCAGAAGATCAAGAATTGTTGACCAAACATGGTCTAACACTTGCACCCATTGGGTTTAAATCCCACTCCCATCCAATATCCAAAATGATAGAGAATCACCTTCTTTATATTTGCGTGCCTAGTTTGCTTTCTAGCTTTAGGTCAGTTGCTTTTTTTTCTCTTAGAGAGTCCAAAGTGAATTCCTTCCTTAAGATGCATTCTGTCTTTTCCCATGGAAAAATAAAATCTTTAGGGATGTACAATGCCATAATTGATGGGAAAGATAAATATAGGTATGGTGATGTGGAATTTTCATCCTTTAGGGATAGAGTGATTGGTCTTAGAGACCAATGCCTTACACGTAATAAATTTCCAAAAGTCCTTTTCCTTCATGATGAATTGCACTTTTTGAGCCCCTTCGATATGGCTTTCCTATTTGAGACGATCCCAGAAATTGATAGGGTTGTTGCAACCACAGTTTTTCCAATTGAACTTCTGTTTGGGGACAAGGTCTCTAAGGAACCCAGGGTTTACACATACAAAGTGCATGGGTCCTCCTTTTCCTATTACCCAGACGGGGTGGCTTCAGAATGCTATGAGCAGAACCTAGCCAATTCAAAGTGGCCCTTTACTTGCAGTGGAATTCAATGGGCGAACCGGAAAATAAGAGTCACTAAGCTTCAAAGCCTTTTTGCACATCATGTCTTTTCATTTGACAGAGGGAGGGCTTGTAATGAATTTAATCATTTCGACAAACCTAGCTGTCTACTTGCGGAAGAAATGCGCCTTTTGACCAAAAGGTTTGACAAGGCAGTTATCAACAGAAGCACAGTTTCTTCCCTCAGTACATACATGGCTTGTCTTAAAACTGCAAATGCGGCTTCAGCAGTTGCAAAATTACGACAGCTGGAGAAGAGAGATTTGTACCCTGATGAGTTAAATTTTGTCTACTCTTTCGGAGAGCATTTCAAAAGTTTCGGGATGAGAGATGACTTTGATGTGTCAATTTTACAATGGGTTAAAGACAAATTTTGTCAGGTCATGCCTCACTTCATCGCCGCTAGTTTTTTTGAGCCGACAGAATTTCATTTAAATATGCGCAAACTGTTGAATGATCTGGCTACTAAAGGAATAGAGGTCCCCCTTTCTGTGATCATCCTGGATAAAGTAAATTTTATAGAAACGAGGTTTCATGCTAGCCTGTTTGAAATAGCAGATGCCATTGGTGTTAACCTGTCAACTCTAGGCAAGAGATTTGACTTTGAAGCTGAATCAGAGCCTTACTTTTCCGAAAATGGATTCATTCACATGCCTTCCAAAGCAAATCCAGATAGGAATTGGATCTTAAATTCTGGCGCACTTAAAATTGATTATTCAAGATTGGCAAGAGCAAGAAGGTTTAGGCTTAAAAGAGATTTTATTGACCTTATCCTCAAAAGGGAAACTCCTAGGAGTCAGCTCTTTTTGGAGTTCAATTCCAGCTCCAAATCTCAAAGCAAAAGTGAAAGTGGAGAAGAAAGGAAATGTTTGGAACGTAAAGGGGACCCCGAAAGCAGTTCAACACTCGAATTGCCAAGTGCCCCAATGTGCACTGATGATGGACAAGGTTTTGAAGGTTCACTTCCGATTGACTTAATCAATTCCTTTGAATCAGAGGAAGTGAAACTCCCCAAGAGAAAGAGGAAAAATGACTGCGTTTTCAAGGCTATTTCAGCTCATCTGGGGATTGACGTCCAGGACCTTCTGAACTTTTTAGTTAATGAGGACATTTCCGATGAGTTGCTTGACTGCATTGAGGAAGATAAAGGCCTGTCACATGAAATGATTGAAGAGGTTTTGGTGACCAAAGGCCTTTCGATGGTTTACACGTCTGATTTCAAGGAGATGGCGGTTCTCAACAGAAAATACGGAGTTAATGGAAAAATGTACTGCACAATTAAGGGTAACCACTGTGAGCTAAGTTCAAAGGAGTGTTTCATCAGGCTGCTAAAAGAAGGTGGTGAGGCTCAAATGTCAAATGAAAACCTGAATGCTGATTCTTTATTTGACCTTGGAAAATTTGTGCATTCTAAGGAACGAGCTGTCAAGCTGGCAAAATCTATGGCCAGAGGCACGACAGGTCTCTTGAGTTCATTTGACCCAGCGTTCTGCAGGGATATGGTCACTCTCTCGGAGCTATTCCCCGAAAACTTTTGTTCAGTGGTCGGATTGAGGTTGGGCTTTGCCGGCTCGGGGAAAACTCACAAGGTTATTCAATGGATCAATTACACTCCAAGTGTCAAAAGGATGTTCATCAGTCCAAGGAGAATGCTGGCCGACGATGTTGAGCCCCAACTTAAAGGGACAGCTTGCCAAGTGCATACATGGGAAACTGCTTTAAAGAAAATTGATGGAACTTTCATGGAAGTGTTTGTTGATGAAATAGGCCTGTACCCACCTGGATACCTCACTTTGTTGCAAATGTGTTCGTTTAGAAGAATCGTCAAAGGACAAAGTGAAAGAATTTTGAAGGGCAAGCTGGCTGAATTATCAAAGACATGTCTAAACATAAGGTGTTTTGGGGATCCACTGCAACTAAGGTACTATTCAGCTGAAGACACAAATCTGTTAGATAAAACCCATGAGATCGACATGATGATCAAGACCATTAAGCACAAGTACTTATTGCAGGGTTACAGATTTGGGTCATGGTTCTCAGATCTGATTGAAATGCCAACAAGGGTCGATGATTCTAAATTTGACAGAAAATTCCATGCAGATATTTCTACAGTCAAAACTGAGGAATATGGGTTGATTCTTGTTGCGAGGAGAGAGGATATGGGGGTTTTTGCGGGAAGCATACCAGTAGCAACGGTGAGTGAATCTCAAGGCATGACCATTGAAAAGAGAGTTCTTATATGCCTTGACCAATATCTTTTTGCTGGCGGTGCCAACTCAGCGATTGTAGCCATAACAAGATCAAAAGTTGGGTTTGACTTCGTCCTAAAAGGAAACAGTCTAAGGGAGGTCCAAAAGATGGCACAAAAGACATTATGGCAGTTGATTCTTGAAGGGAAAAAGATACCAATGGAGAGAATCGTGAACATGAATCCTGGAGCAAGTTTCTATGAAAGTCCGCTTGATGTGGGGAATTCATCAAGACAAGACAAAGCATCAAATGACTTGTTTATAATGCCTTTCATAAATTTAGCTGAGGAAGAAGTTGACCCGGAAGAAATGGTCGGAGATGTGATTCAGCCGGTTGAGTGGTTTAAGTGCCACATCCCGGTTTTCGACACAGACCCCATGTTGGCTGAAATTTTCGAAAAAGTCGCATCCAAAGAAAAAAGAGAGTTCCAGTCAATTCTGGGGATGTCCAACCAATTTCTGGACATGGAGAAAAATGGATGCAAAATTGACATTCTCCCATTTGCTAGACAGAATGTGTTTCCACACCACCAAGCGTCTGATGATGTAACTTTTTGGGCAGGGGTCCAAAAAAGGATTAGAAAATCAAACTGGAGAAGAGATAAGACGAAGTTCGAAGAATTTGAGTCCCAAGGGAAAGAGCTATTGAGCGAGTTCCTCCAAATGCTGCCCTTTGAGTTCAAAGTGAACATCAAGGACATAGAGGAGGGTGAAAAAAGTTTTCTGGAGAAGCGCAAGCTGAAGTCAGAAAAAATGTGGGCCAATCATGCGGAAAGATCGGACATTGACTGGAAACTGGACCATGCCTTCCTGTTTATGAAGTCTCAGTATTGCACGAAAGAAGGAAAGATGTTCACTGAGGCTAAGGCTGGTCAAACCTTAGCATGCTTCCAACACATTGTTCTCTTTAGATTTGGGCCAATGCTCAGAGCCATTGAAAGTGCCTTCTTGAGGAGCTGTGGAGAGTCCTACTACATTCACTCTGGAAAAAATTTTTTTTGCCTTGATAGCTTTGTAACAAGAAACGCAGAGGTGTTTGATGGGTTCTCCATAGAATCGGACTACACTGCTTTTGATTCTTCACAGGATCATGTAATTCTCGCATTTGAGATGGCCTTACTGCAGTACCTGGGGGTTTCAAAAGAGTTTCAAATGGACTATCTTAGATTGAAACTCACGCTGGGATGTAGATTGGGTTCTTTGGCCATTATGCGATTCACTGGTGAGTTCTGCACTTTCCTTTTCAATACTTTTGCAAACATGCTATTCACTCAGTTGAAGTACAAGATTGATCCCAGAAAACATAGGATATTGTTTGCCGGGGATGACATGTGTTCACTAAGCTCTTTGAGGAGAAGAAGAGGGGAGAAAGCCACAAAACTTTTAAAAAGTTTCTCGCTCACCGCAGTTGAAGAGGTAAGGAAGTTCCCAATGTTCTGTGGATGGTACCTGAGCCCATATGGGATAATCAAGTCACCAAAGCTTTTGTGGGCCAGAATCAAAATGATGAGTGAAAGACAACTTTTGAAGGAGTGTGTAGACAACTACCTATTTGAGGCAATATTTGCCTACAGGTTAGGTGAGAGGCTTTATACAATCTTGAAAGAGGAAGACTTTGAGTATCATTACTTAGTCATAAGATTTTTCGTGAGAAATTCAAAGCTCCTGACAGGTTTGAGTAAGAGCCTAATCTTTGAAATAGGTGAAGGAATTGGGTCCAAATGGCAATTGTCAACGTCAACCGTTTCCTCAAAGAAGTTGAGTCAACAGATCTTAAAATTGACGCCATATCCTCCTCTGAATTGTATAAAGATGCAACCTTCTTCAAACCAGACGTGCTCAACTGTATCAAAAGATTTGAATCGAATGTCAAAGTGTCATCACGATCTGGAGACGGACTTGTGCTCTCAGATTTCAAACTGCTGGACGACACTGAGATCGACGCAATCAGAAAGAAAAGTAACAGGTACAAGTATTTGCACTATGGAGTTATTCTGGTCGGGATCAAAGCTATGCTTCCAAACTTTAGAGGCATGGAGGGGAGGGTCATTGTGTATGACGGTGCATGCTTGGACCCAGAAAGAGGGCACATCTGTTCATATCTTTTCAAATTTGAGTCGGACTCCTGCTACTTTGGGCTCAGACCAGAGCATTGTCTTTCCACCACTGATGCAAATTTGGCAAAAAGGTTTAGGTTCAGGGTGGACTTTGATTGTCCACAATATGAGCAGGACACAGAACTCTTTGCTCTTGACATTGGAGTTGCATACAGGTGCGTCAACTCAGCAAGGTTCTTGGAAACCAAAACAGGGGATTCAGGTTGGGCCTCCCAGGCGATTAGTGGATGTGAGGCACTCAAATTCAATGAAGAAATTAAAATGGCAATACTTGACCACAAATCGCCACTGTTTCTGGAAGAAGGTGCACCAAACGTGCACATTGAAAAGAGAATGTTTAGAGGTGACAAGGTTAGAAGGTCGCGCTCTATTTCCGCAAAAAGGGGGCCAAACTCAGAACAGCAGGAAAAGAGAGGATTTAGGTCCCTCTCAGCTAGAATCGAAAGATTTGGAGAAAATGAGTTTGGAAGACGTGCTTCAACAAGCGAGGCGCCACCGGGTAGGAGTGTATCTGTGGAAGACCCATATAGACCCGGGAAAGGAACTTCTGACGGTTCCTCCCCCTGAAGGATTTAGAGAAGGTGAAAGCTTTGAAGGCAGAGAGCTTTACCTTCTTCTCTGCAACCATTATTGTAAATATTTATTTGGTAATGTTGCTGTTTTTGGGTCTTCTGACAAGACCCAGTTGGCCGCTGTTGGATTTGATACCCCTCCGGTTCATTACAATCTGACAACGATCCCGAAACAAGGGGAAACTGAAGATGAAAAGAAGGCCAGAGAAGGGACGTCTGGCGAGAAAACAAAAATTTGGAGGATCGACTTGTCAAGTGTTGTACCTGAATTGAAAACTTTTGCTGCCACTTCTAGGCAGAACTCTTTGAACGAATGTACGTTCAGAAAGCTTTGTGAGCCATTCGCTGATTTGGCTCGTGAATTTCTTCACGAAAGGTGGTCAAAAGGACTGGCCACTAATATTTACAAGAAATGGCCCAAAGCTTTTGAAAAAAGCCCATGGGTGGCATTTGATTTTGCCACCGGTCTAAAAATGAATCGTCTGACACCTGATGAAAAGCAGGTGATAGATAGAATGACTAAGAGGCTTTTTCGTACAGAAGGACAAAAGGGGGTTTTCGAGGCAGGTTCGGAGAGTAATCTGGAACTGGAGGGTTAGGAGTCGTATAAAATTCCGCAAATTTGGTCGCGGTCTTGCAGGTTGACATGCCTGCCTTTATACTTAAATAAAGGGTTCCCCCGGTTTTCTGTGTATTTCCGGGTTAGTGTGGTTTTTCTAGAGTCTAGAGTTTGTCCTGTCCTGGATAA

1. **[Apple stem grooving virus, complete genome](https://www.ncbi.nlm.nih.gov/nuccore/NC_001749.2/). Accession number: NC_001749.2 (6495 bp).**

AAATTTAACAGGCTTAATTTCCGCGCTTTACGTCAATGGCTTTCACTTACAGAAACCCCCTCGAAATTGCAATCAACAAACTTCCTAGTAAGCAGTCTGATCAACTGCTTTCCTTGACCACCGACGAGATTGAAAAGACCTTAGAAGTGACCAACCGCTTCTTCTCTTTTTCAATCACACCAGAAGATCAAGAATTGTTGACTAAGCATGGTCTAACACTTGCACCTATAGGGTTTAAGTCACACTCCCATCCAATATCCAAAATGATAGAAAATCATCTCCTGTATATATGTGTTCCGAGTCTTTTATCCTCCTTTAAGTCAGTTGCCTTTTTTTCACTTAGGGAAAATAAAGTAGACAGTTTTCTTAAGATGCATTCAGTCTTTTCCCATGGAAAAATTAAATCTTTGGGGATGTACAATGCTATAATTGATGGGAAAGATAAATATAGGTATGGTGATGTAGAGTTTTCATCTTTTAGGGATAGAGTGATTGGTCTTAGAGATCAATGCCTTACACGTAACAAATTTCCAAAAGTTCTGTTTCTTCACGACGAGTTGCACTTTCTAAGTCCATTTGACATGGCTTTCCTATTTGAGACAATCCCAGAAATTGATAGAGTTGTTGCAACCACAGTTTTTCCAATAGAACTTTTATTCGGGGACAAGGTCTCTAAGGAACCCAGGGTTTATACCTACAAGGTCCATGGCTCTTCATTTTCATTTTATCCGGATGGTGTTGCCTCTGAGTGTTACGAACAGAATTTGGCAAATTCTAAATGGCCCTTCACCTGCAGCGGCATACAATGGGCTAACAGGAAAATTAGGGTAACCAAGCTACAGAGTCTCTTCGCCCATCATGTTTTCTCATTTGACAGGGGGAGGGCTTGTAATGAATTTAATCATTTCGACAAACCTAGCTGTCTACTTGCGGAAGAAATGCGCCTTTTGACCAAAAGGTTTGATAAAGCAGTTATTAACAGAAGCACAGTCTCTTCCCTCAGTACATACATGGCTTGTCTTAAAACTGCAAATGCGGCTTCAGCTGTTGCCAAGCTGAGGCAGTTGGAGAAGAGGGATCTTTACCCAGATGAGTTGAACTTCGTCTATTCCTTTGGAGAGCATTTCAAAAATTTTGGGATGAGAGATGACTTTGATGTGTCAGTTCTACAATGGGTCAAAGACAAATTTTGCCAGGTCATGCCTCACTTCATCGCCGCCAGTTTCTTTGAACCAACAGAATTTCATTTAAACATGCGCAAATTGTTGAATGATCTGGCTACTAAAGGAATAGAGGTTCCCCTTTCTGTGATCATCCTGGACAAAGTCAACTTCATAGAGACCAGATTTCATGCCAGGATGTTCGACATAGCACAGGCAATCGGGGTGAACCTAGATTTACTGGGGAAAAGATTTGATTATGAGGCTGAGAGTGAAGAGTACTTTTCAGAGAACGGTTACATCTTTATGCCCTCTAAATCAAATCCAGAGAGAAATTGGATTCTAAATTCCGGTTCGCTGAAAATTGACTATTCAAGATTGGTAAGAGCCAGGAGATTTAGATTGAGAAGAGATTTCCTAGATCCCATATCTAAAGGAAAATCCCCTAGAAAACAACTCTTCTTGGAGTCAACGGGAAACATTAAATCAAATCCCAATGCTGAAAAAAATAGCGAGAGCGGCGAAATAAAGATTGAAGGCAGTGCCGAAAATGATCAGCCACATGAGGTATCACATACTTCAATGGAAACCGAGGATGGACAGGGTTTTGAAGGTTCAATACCAGTTGATTTAATCAATTGCTTTGAACCAGAAGAAATCAAGCTTCCAAAGAGAAGAAGGAAAAATGATTGCGTCTTCAAGGCCATCTCTGCACACTTGGGGATTGACTCTCAAGATTTGTTGAATTTTTTGGTAAATGAAGACATATCAGATGAATTACTTGATTGCATTGAAGAGGACAAAGGACTGTCACATGAAATGATTGAAGAAGTTTTGATCACAAAGGGTCTTTCAATGGTTTATACTTCTGACTTCAAAGAAATGGCAGTTCTTAATAGAAAGTATGGAGTGAATGGCAAGATGTACTGCACAATTAAAGGCAATCACTGCGAGCTGAGTTCCAAAGAGTGCTTCATCAGATTATTGAAAGAAGGTGGTGAAGCGCAGATGTCAAATGAAAATCTAAATGCTGATTCCTTGTTCGACCTTGGAAGATTTGTGCATAATAGAGACAGGGCTGTCAAGCTAGCAAAATCAATGGCAAGAGGCACAACAGGCCTCCTGAATGAATTCGACCTAGAATTCTGCAAGAACATGGTGACCCTTTCGGAGTTGTTTCCTGAAAACTTTTCTTCTGTTGTCGGGCTAAGGCTTGGGTTTGCGGGTTCTGGTAAAACGCATAAGGTGCTTCAATGGATTAATTACACTCCAAGTGTCAAAAGAATGTTTATAAGTCCAAGGAGAATGCTGGCGGATGAAGTTGAACCTCAACTCAAGGGAACGGCCTGTCAGGTGCATACATGGGAGACTGCACTTAAAAAAATCGACGGAACTTTTATGGAAGTTTTTGTTGATGAAATAGGTTTGTACCCACCTGGATACCTTACACTGCTACAGATGTGTGCTTTCAGAAAGATTGTTAAGGGACAAAGTGAAAATTTCTTGAAAGGCAAACTGTTGGAATTGTCAAAGACTTGCTTAAACATAAGATGTTTTGGTGATCCATTGCAATTAAGGTATTACTCAGCTGAAGACACCAATCTATTGGACAAAACACATGATATTGACCTCATGATCAAGACGATCAAGCACAAATATCTTTTCCAAGGGTACAGGTTCGGTCAGTGGTTTCAAGAACTGGTGAACATGCCCACTAGAGTGGATGAGTCGAAATTCTCAAGGAAGTTCTTTGCAGACATTTCAAGTGTAAAAACTGAAGATTACGGACTCATCCTAGTTGCCAAGAGAGAAGATAAAGGTGTCTTCGCTGGAAGAGTTCCTGTAGCAACAGTGAGTGAATCTCAGGGAATGACCATTAGCAAAAGGGTGTTGATATGTTTGGACCAAAATCTTTTTGCCGGGGGAGCCAATGCAGCCATTGTTGCAATAACAAGATCAAAGGTCGGCTTTGACTTTATCCTTAAAGGGAATTCATTGAAAGAGGTACAGAGGATGGCACAAAAGACAATTTGGCAGTTCATCATTGAAGGGAAGTCTATTCCGATGGAGAGGATAGTGAACATGAATCCTGGAGCCAGCTTTTATGAGAGTCCTTTGGATGTTGGAAATTCATCAATTCAAGACAAAGCTTCTAATGACCTGTTCATAATGCCTTTTATAAATTTGGCTGAGGAAGAAGTTGACCCAGAGGAAGTTGTTGGGGACGTAATTCAACCTGTTGAGTGGTTCAAATGTCATGTGCCTGTCTTCGACACAGATCCGACGCTTGCGGAGATTTTTGATAAGGTTGCAGCAAAAGAAAAAAGGGAATTCCAGTCTGTGCTGGGTCTTTCAAATCAATTTCTTGACATGGAAAAGAATGGATGCAAAATAGACATCTTGCCCTTTGCGCGACAAAATGTTTTTCCACATCATCAAGCGTCTGATGATGTTACTTTCTGGGCAGGTGTTCAAAAAAGAATTAGAAAGTCGAACTGGAGAAGGGAGAAATCGAAGTTTGAGGAATTTGAAAGCCAAGGGAAAGAACTTCTTCAAGAATTCATCTCAATGCTACCGTTTGAATTCAAAGTGAATATCAAGGAGATTGAAGATGGAGAGAAGAGCTTTTTAGAAAAAAGAAAGCTAAAATCTGAGAAAATGTGGGCAAATCATTCGGAGAGATCAGACATTGACTGGAAACTTGACCACGCCTTTCTCTTCATGAAATCACAATATTGCACGAAGGAAGGGAAGATGTTCACCGAAGCTAAAGCTGGCCAAACTTTGGCCTGTTTCCAACATATAGTCCTATTTAGATTTGGACCCATGTTGAGAGCAATTGAAAGTGCCTTTTTGAGAAGCTGTGGAGACTCATACTACATACACTCCGGGAAAAACTTCTTCTGCCTGGATAGCTTTGTGACAAAGAATGCAAGTGTCTTTGATGGATTTTCAATTGAGTCAGACTACACGGCCTTTGACTCATCTCAGGACCACGTCATATTGGCCTTTGAAATGGCACTGTTACAATACCTGGGCGTGTCAAAGGAGTTTCAGCTAGATTACCTTAGACTGAAATTAACTCTCGGATGCCGTCTCGGATCACTAGCAATAATGAGGTTCACAGGAGAATTTTGCACTTTCTTATTCAACACATTTGCCAATATGCTGTTTACTCAATTGAAGTACAAGATAGACCCAAGGAGGCATAGGATTTTATTTGCTGGGGACGATATGTGTTCCTTGAGCTCTCTCAAAAGAAGGAGAGGGGAGAGAGCGACAAGATTGATGAAGAGCTTTTCCCTAACTGCAGTAGAAGAGGTGAGAAAATTCCCAATGTTTTGTGGATGGTACTTAAGTCCATATGGTATCATTAAATCTCCAAAATTGCTGTGGGCCAGGATCAAGATGATGAGTGAGAGACAGCTTTTGAAGGAATGTGTTGATAATTACCTATTTGAGGCGATATTTGCCTACAGATTAGGTGAGAGGCTTTACACAATTTTGAAAGAAGAGGATTTTGAATACCATTATCTTGTCATAAGATTTTTTGTTAGAAATTCAAAATTGTTAACAGGGTTGAGCAAAAGCTTGATATTTGAAATTGGGGAGGGCATCGGGTCCAAATGGCTATCGTCAACGTCAACCGCTTCCTCAAGGAGGTCGAATCTACAGACCTCAAAATTGATGCTATCTCGTCCTCAGAGCTTTACAAGGATGCAACCTTTTTCAAACCAGACGTGCTTAATTGCATCAAAAGGTTTGAATCAAACGTCAAGGTTTCCTCTCGATCTGGTGACGGCCTCGTCCTGTCTGATTTCAAACTGCTTGATGACACCGAAATTGATTCAATCAGGAAGAAAAGCAACAAGTACAAATACTTACACTATGGAGTCATCCTGGTTGGGATCAAAGCAATGTTGCCAAACTTTAGAGGCATGGAAGGGAGAGTCATTGTATATGATGGAGCCTGCCTGGATCCGAAAAGAGGCCACATTTGCTCGTATCTTTTCAAGTTTGAGTCTGACTGTTGCTACTTTGGTCTCAGGCCAGAGCACTGTTTGTCTACCACAGACGCAAATTTGGCCAAAAGGTTTAGATTTCGTGTGGACTTTGATTGTCCACAATATGAACAGGACACTGAGTTGTTTGCTCTTGACATTGGAGTTGCATACAGATGCGTCAACTCTGCAAGGTTTTTGGAAACCAAAACTGGCGATTCAGGATGGGCTTCACAGGCAATCAGCGGCTGTGAAGCACTTAAATTCAATGAGGAAATCAAGATGGCCATCCTGGATCGCAGATCCCCGCTGTTTCTGGAAGAAGGTGCACCAAACGTGCACATTGAAAAGAGATTGTTCAGAGGTGACAAGGTTAGAAGGTCACGCTCAATTTCCGCTAAAAGGGGGCCAAACTCAAGGGTGCAAGAAAAGAGAGGATTTAGGTCCCTCTCGGCTAGAATTGAAAGATTTGGAAAAAATGAGTTTGGAAGACGTGCTTCAGCAAGCGAGGCGCCACCGGGTAGGAGTATATCTATGGAAGACTCACATAGACCCGGCAAAGGAACTTCTGACGGTTCCTCCCCCTGAAGGATTTAAGGAAGGTGAAAGCTTTGAGGGCAAAGAGCTTTACCTTCTTCTTTGCAACCATTACTGTAAATACTTGTTCGGTAATATTGCTGTCTTTGGGTCATCTGATAAGACCCAGTTTCCCGCTGTTGGATTTGATACACCTCCGGTTCATTATAATTTGACAACGACCCCAAAGGAAGGGGAGACTGACGAAGGAAGGAAGGCCAGAGCGGGTTCGTCTGGCGAAAAAACAAAAATTTGGAGGATCGATTTGTCAAATGTTGTTCCTGAATTGAAAACCTTTGCTGCCACTTCCAGGCAGAACTCTTTGAACGAATGTACGTTCAGAAAGCTTTGCGAGCCATTTGCCGATTTGGCTCGAGAATTTCTACATGAAAGGTGGTCTAAGGGATTGGCCACCAATATTTACAAGAAATGGCCCAAAGCTTTCGAAAAAAGTCCATGGGTGGCCTTTGATTTTGCCACTGGTCTGAAAATGAATCGTCTAACACCTGATGAGAAACAGGTGATTGATAGAATGACCAAAAGACTTTTTCGTACTGAAGGACAAAAAGGGGTTTTCGAGGCAGGTTCGGAAAGTAACCTGGAACTGGAGGGTTAGGAGTCGTGTGAAATTCCGCAAACTTGGTCGCGGTCTTGCAGGTTGACATGCCTGCCTTTATACTTAATTAAAGGGTTCCCCCGGTTTTCTGAGCATTTCCGGGTTAGTGTGGTTTTTCTAGAGTCTAGAGTTTGTCCACTCT

1. **Apple stem grooving virus isolate 13TF138, complete genome. Accession number: MZ126527.1 (6486 bp).**

CAGCGCTTAATTTCCGCGCTTTACGTCAATGGCTTTCACTTACAGAAATCCCCTCGAAATTGCAATCAACAAGCTTCCAAGTAAGCAGTCTGATCAACTGCTTTCTTTAACCACAGACGAGATTGAAAAGACCTTAGAAGTGACTAATCGCTTCTTCTCTTTTTCTATCACTCCAGAAGATCAAGAATTGTTGACCAAGCATGGTCTAACACTTGCACCCATTGGATTCAAGTCACATTCCCATCCTATATCCAAAATGATAGAAAATCACCTTCTGTACATATGCATTCCAAGTCTTTTATCTTCTTTTAGATCCGTAGCCTTTTTCTCGCTTAGAGAAAATAAAGTTAATAATTTTCTTAAGATGCACTCAGTTTTTTCACATGGAAAAATTAAGTCTCTAGGCATGTACAACGCCATCATTGATGGAAAAGATAAATACAGGTATGGCGAAGTTTCTTTTTCTTCCTTTAGAGATAGGGTGATTGGTCTCAGGGATCAGTGCCTCATGCGTAATAAATTTCCAAAAGTTCTCTTTCTCCACGATGAACTTCATTTCCTAAGTCCTTTCGACGTTGCTTTTCTATTTGAGACAATCCCAGAAATTGATAGAGTCATAGCAACTACTGTTTTTCCGATTGAACTGCTTTTTGGGGACAAGGTTTCAAAGGAGCCCAGAGTTTACACCTACAAGGTCCATGGTTCCTCCTTTTCATTCTATCCAGATGGTGTAGCCTCTGAGTGCTATGAGCAAAATCTTGCAAACTCCAAATGGCCGTTCACTTGTAGTGGAATTCAGTGGGCTAACAGGAAAATAAGGGTTACTAAGTTGCAGAGCCTCTTTGCTCATCACGTATTTTCATTTGATAGAGGCAGGGCTTGCAACGAATTTAACCACTTTGACAAGCCGAGTTGTCTCCTTGCAGAAGAAATGCGCCTTTTGACCAAAAGGTTTGATAAGGCAGTCATTAATAGGAGTACGGTTTCTTCTTTGAGCACTTATATGGCTTGTCTCAAGACCGCCAATGCAGCTTCTGCTGTCGCCAAGCTGAGGCAACTAGAGAAAAGGGACCTCTATCCGGATGAACTAAACTTCGTTTATTCCTTTGGGGAGCATTTCAAAAATTTTGGAATGAGGGACGATTTTGATGTGTCAGTTCTGCAATGGGTAAAGGACAAATTCTGTCAAGTTATGCCTCACTTCATTGCTGCCAGTTTCTTTGAGCCAACTGAGTTCCATTTGAATATGCGAAAATTATTGAATGACCTGGCTACTAAAGGGATAGAGGTTCCACTTTCAGTAGTGGTTTTAGATAAGGTAAATTTTATAGAGACCAGATTTCATGCCAGAATGTTCGAAATTGCCGAAGCTATTGGAGTCAGTTTAAGTCTACTTGGAAAAAGGTTTGACTATGAAGCTGAAAGTGAAGAATATTTTTCAGAAAATGGCTATCTTTTCATGCCTTCAAGGACAAATCCAAGTAGAAACTGGATCTTAAACTCTGGGTCTGTCAAAATTGATTTCCATAAGTTGCAGAGGGCAAGGAGGTTCAGATTGAGAAGAGATTTCGTTGATTTGATTGCTAGAAGGGAAACCCCTCGTAAACAACTATTCCTCGAGTCAGCTCAAACTCTCCAATCAACGTCTAAAAGTGTGGGGGAGAACATGGACAGTGGAGTCAAATCCGACGAAAGCAGTAAAAAAATACAGTCACTCGAGGTGCCCTGTGTCCCGATGTCCACTGAAGATGGGCAAGGCTTTGAAGGTTCAATTCCAATTGACTTGATAAATTCCTTCGAGCCAGAAGTAATCAGACTTCCGAAAAAGAAGAGAAAGAATGATTGTGTTTTTAAAGCCATCTCTGCATACCTCGGCATTGACGTTCAGGACCTCTTGAACTTTTTGGTGAATGAAGACATATCCGATGAGTTGCTTGAATGCATAGATGAAGATAAAGGCCTGTCGCACGAGATGATAGAGGAAGTTCTGGTGACGAAAGGGATTTCTATGGTCTACACCTCAGATTTCAAAGAGATGGCAGTTCTCAACAGGAAGTATGGGGTTAATGGAAAAATGTACTGCACAATCAGGGGGAATCATTGTGAGCTGAGTTCAAAGGAATGCTTCATTAGATTGCTGAAGGAGGGTGGTGAAGCACAAATGTCCAATGAGAATTTGAATGCTGATTCCTTGTTTGATCTTGGAAAGTTCGTGCACAACAGAGAAAGAGCCACAAAATTGGCAAAATCGATGGCTAGGGGCACAACCGGTCTTTTGACTGAATTTGATCCAGAATTTTGTAAAAGTATGGTCACACTTTCGGAAATGTTTCCGGAGAACTTCTCCTCTGTTGTCGGACTGAGGTTGGGCTTCGCTGGTTCCGGAAAAACCCATAAAGTTCTTCAATGGATCAACTATACACCAAGCGTCAAAAGAATGTTTATTAGTCCAAGGAGAATGCTGGCCGACGAGGTTGAAATTCAATTAAGAGGGACATCTTGTCAAGTTCACACTTGGGAGACAGCTCTTAAAAAAATTGATGGAACCTTCATGGAGGTCTTTGTGGATGAAATTGGCCTTTACCCACCTGGATATCTCACTCTTCTACAAATGTGTGCTTTCCGAAGGATCGTTAAGGGGCAAAGTGAAAAGTTTTTAAGAGGAAAGCTTGCTGAACTTTCGAAGACCTGTTTGAATGTAAGGTGTTTTGGTGACCCCTTGCAAATGAGGTATTATTCTGCAGAGGATACAAACCTTCTAGACAAGACACATGAGCTTGATCTAATGGTCAAAACCATCAAACACAAGTACCTTCTTCAAGGGTACAGGTTCGGCCAGTGGTTCCAAGAGCTGATAAACATGCCAACTAGAATGGACGATTCGAAATTTTCAAGGAAATTTTTTGCGGACATTTCTAGTGTCAAAACCGAAGATTATGGACTCATACTTGTCGCCAAGAGGGAAGACAAGGGGACGTTTGCCGGGAGAATACCTGTGGCTACGGTCAGCGAATCACAGGGAATGACTATAAGCAAGAGGGTGCTAATATGCTTAGACCAAAATCTGTTTGCCGGGGGAGCCAATGCCGCCATAGTTGCGATCACTAGATCGAAAGTTGGGTTTGACTTTATTCTCAAAGGGAATTCACTAAAAGAGGTGCAGAAAATGGCCCAAAAAACTTTGTGGCAGTTCGTACTTGAAGGAAAGACCATACCGATGGAAAGAATTGTCAATATGAACCCTGGAGCCAGTTTTTATGAGAGCCCCTTAGACGTTGGCAACTCGTCCATTCAGGACAAAGCCTCACATGACTTGTTCATAATGCCTTTTATCAATTTAGCTGAAGAAGAAGTTGACCCTGAGGAAATCGTCGGAGATGTTATTGAACCAGTTGAGTGGTTCAAATGCCATATACCGGTTTTTGATACAGACCCGATGCTTGCAGAGATATTTGACAAAGTGGCAGCAAAGGAAAAGAGAGAATTTCAGTCCATACTTGGGCTTTCGAATCAATTCCTTGACATGGAAAAAAATGGATGCAAAGTCGATATTTTGCCTTTTGCTAGACAGAATGTTTTCCCTCACCATCAAGCTTCAGATGACGTCACCTTTTGGGCAGGTGTTCAGAAGAGGATAAGGAAGTCAAATTGGAGACGGGAAAAGTCGAAATTTGAGGAGTTTGAAGCTCAGGGGAAGGAATTGCTCAGTGAATTCATATCAATGTTGCCCTTTGAATTCAAGGTGAACATCAAGGACATTGAAGAAGGTGAAAAAAGCTTTCTTGAAAAGAGAAAGTTAAAGTCTGAAAAGATGTGGGCCAACCACTCAGAAAGATCTGACATTGACTGGAAACTTGACCATGCCTTTCTATTTATGAAATCTCAATATTGCACAAAGGAAGGCAAAATGTTCACTGAGGCAAAAGCTGGTCAAACCTTAGCATGTTTCCAACACATTGTTCTGTTTAGGTTTGGTCCCATGCTTCGAGCAATTGAAAGGGCTTTTCTTAGAAGTTGTGGAGAGTCATATTACATACATTCAGGAAAAAACTTTTTCTGCTTGGACAGCTTTGTGACAAAAAACGCAGAAGTCTTTGATGGGTTTTCTATTGAATCTGACTACACGGCTTTTGACTCATCTCAGGACCACGTAATTTTGGCCTTTGAAATGGCACTTTTGCAGTACCTAGGGGTGTCCAAAGAGTTTCAGTTGGATTATTTGAGATTGAAATTGACCCTTGGCTGCAGACTTGGATCTCTAGCCATTATGAGGTTCACTGGGGAATTCTGCACTTTTTTGTTCAATACATTTGCAAACATGCTTTTCACCCAGTTGAAATACAAAATTGACCCGAGGAAGCATAGGATTCTATTTGCTGGAGATGACATGTGCTCTCTAAGTTCTCTCAAAAGAAGAAAAGGGGAAAAGGCAACAAGACTGTTGAAGAGCTTTTCTTTAACTGCTGTGGAAGAGGTACGGAAGTTTCCAATGTTCTGTGGGTGGTACTTGAGCCCATATGGGATTATCAAGTCTCCAAAGTTGTTGTGGGCCAGGATCAAAATGATGAGTGAAAGACAACTTTTAAAGGAATGTGTGGATAACTATCTGTTTGAAGCTATATTTGCCTACAGATTAGGTGAGAGGCTTTACACAATTTTGAAAGAAGAGGACTTTGAGTATCATTACCTTGTTATAAGGTTTTTTGTCAAAAATTCTAAGTTGCTGACAGGGTTGAGCAAAAGCCTCATTTTCGAAATTGGTGAAGGGATTGGGTCAGAATGGCAATCGTCAATGTCAACCATTTCCTCAAGGAAGTCGAGTCAACAGATCTCAAAATTGATGCCATCTCTTCCTCGGAATTGTACAAAGATGCCACTTTTTTTAAACCAGATGTGCTCAACTGTATCAAACGATTTGAATCAAATGTCAAGGTGTCTTCAAGGTCAGGTGATGGACTCGTATTGTCTGATTTCAAACTACTTGATGACACAGAGATCGACTCCATTAGAAAGAAGAGCAACAAATACAAGTATCTACATTATGGAGTCATTCTGGTCGGGATTAAGGCTATGCTACCGAACTTTAGGGGAATGGAGGGGAGGGTCATTGTGTATGACGGTGCATGCTTGGACCCAGAAAGAGGTCACATTTGTTCCTACTTATTCAAATTCGAGTCCGACTGTTGTTACTTTGGGCTTAGACCGGAACATTGCCTATCCACAACCGATGCAAATTTGGCTAAAAGGTTCAGATTTAGAGTGGATTTTGATTGTCCACAATACGAGCAAGACACAGAGCTGTTTGCTCTCGACATTGGGGTTGCATACAGGTGTGTTAATTCAGCCAGATTCTTGGAAACTAAGACTGGTGATTCAGGATGGGCTTCACAGGCAATCAGCGGCTGTGAGGCACTTAAATTCAATGAAGAAATCAAGATGGCAATCCTGGATCACAGATCACCGCTTTTTCTGGAAGAAGGTGCACCAAACGTGCATATTGAAAAGAGAATGTTTAGGGGTGACAAAGTCAGAAGGTCACGCTCTATCTCTGCAAAAAGGGGGCCAAACTCAAGGCTACAGGAAAAGAGAGGATTTAGGTCCCTCTCAGCTAGAATCGAAAGATTTGGAGAAAATGAGTTTGGAAGACGTGCTTCAACAAGCGAGACGCCACCGGGTAGGAGTGTATCTGTGGAAGACACACATAGATCCGGCAAAGGAACTTCTGACGGTTCCTCCCCCTGAAGGATTCAAGGAAGGTGAAAGCTTTGAAGGCAGAGAGCTTTACCTTCTTCTCTGCAACCATTATTGTAAATATTTATTTGGTAATATTGCTGTTTTCGGGTCATCTGACAAGACCCAGTTTCCCGCTGTTGGATTTGATACCCCTCCGGTTCATTACAATCTAACAACGACCCCGAAAGAAGGGGAGACTGAAGAGCAAAGGAAGGCCAGAGAAGGTTCGTCTGGCGAAAAAACAAAAATTTGGAGAATCGACCTGTCAAATGTTGTACCCGAATTGAAAACTTTTGCTGCCACTTCCAGGCAGAACTCTTTGAACGAATGTACGTTCAGAAAGCTTTGTGAGCCATTTGCTGATTTGGCTCGGGAATTTCTTCATGAAAGATGGTCAAGGGGACTTGCCACCAACATTTATAAGAAATGGCCCAAAGCTTTTGAAAAGAGCCCTTGGGTGGCTTTTGATTTTGCCACCGGTCTTAAAATGAACCGTCTAACGCCTGATGAAAAACAGGTAATAGACAGAATGACTAAGAGGCTTTTTCGTACTGAAGGACAAAAAGGGGTTTTCGAGGCGGGTTCGGAGAGCAATCTGGAACTGGAGGGTTAGAAGTCGTGTGAAATTCCGCAAACTTGGTCGCGGTCTTGCAGGTTGACATGCCTGCCTTTATACTTAAATAAAGGGTTCCCCCGGTTTTCTGTGCATTTCCGGGTTAGTGTGGTTTTTCTAGAGTCTAGAGTTTGTCCCCT

1. **Apple stem grooving virus isolate Heilongjiang-1, complete genome. Accession number: MK481981.1 (6487 bp).**

AAATTTAACAGGCTTAATTTCCGCGCTTTACGTCAATGGCTTTCACTTACAGAAATCCCCTCGAAATTGCAATCAACAAGCTTCCAAGTAAGCAGTCTGATCAACTGCTTTCTTTAACCACAGACGAGATTGAAAAGACATTAGAAGTGACCAATCGCTTCTTCTCTTTTTCTATCACTCCAGAAGATCAAGAATTGTTGACCAAGCATGGTCTAACACTTGCACCCATTGGATTTAAGTCGCATTCCCATCCAATATCCAAAATGATAGAAAATCATCTTCTGTACATATGCATTCCAAGTCTTTTATCTTCTTTTAGATCCGTAGCCTTTTTCTCGCTTAGAGAAAATAAAGTCAATAATTTCCTTAAGATGCACTCAGTTTTTTCACATGGAAAAATTAAGTCTCTAGGCATGTACAACGCCATCATTGATGGAAAAGATAAATACAGGTATGGCGAAGTTTCTTTTTCTTCCTTTAGAGATAGGGTGATTGGTCTTAGGGATCAGTGCCTCATGCGTAATAAATTTCCAAAAGTTCTCTTTCTCCACGATGAACTTCATTTCCTAAGCCCTTTTGACGTTGCTTTTCTATTTGAGACAATCCCAGAAATTGATAGGGTCATAGCAACTACTGTTTTTCCGATTGAACTGCTTTTTGGGGACAAAGTTTCAAAGGAGCCCAGAGTTTACACCTACAAGGTCCATGGTTCCTCCTTTTCATTCTATCCAGATGGTGTAGCCTCTGAGTGCTATGAGCAAAATCTTGCAAACTCCAAATGGCCGTTCACTTGTAGTGGAATTCAGTGGGCTAACAGGAAAATAAGAGTTACTAAGTTGCAGAGTCTCTTTGCTCATCACGTATTTTCATTTGATAGAGGCAGGGCTTGCAACGAATTTAACCACTTTGACAAGCCGAGTTGTCTCCTTGCAGAAGAAATGCGCCTTTTGACCAAAAGGTTTGATAAGGCAGTCATTAACAGGAGTACGGTTTCTTCTTTGAGCACTTATATGGCTTGTCTCAAGACCGCCAATGCAGCTTCTGCTGTCGCCAAGCTGAGGCAACTAGAGAAAAGGGACCTTTATCCGGATGAACTAAACTTCGTTTATTCCTTTGGGGAGCATTTCAAAAATTTTGGAATGAGGGACGATTTTGATGTGTCAGTCCTGCAATGGGTGAAGGACAAATTCTGTCAAGTTATGCCTCACTTCATTGCCGCTAGTTTCTTTGAGCCAACTGAGTTCCATTTGAATATGCGAAAATTGTTGAATGACCTGGCCACTAAAGGGATAGAGGTTCCACTTTCAGTAGTGGTTTTAGATAAGGTAAATTTTATAGAGACCAGATTTCATGCCAGAATGTTCGAAATTGCCGAAGCTATTGGAGTCAGTTTAAGTCTACTTGGAAAAAGGTTTGACTATGAAGCTGAAAGTGAAGAATATTTTTCAGAAAATGGCTATCTTTTCATGCCTTCAAGGACAAATCCAAGTAGAAACTGGATCTTAAACTCTGGGTCTGTCAAAATTGATTTCCAGAAGTTGCAGAGGGCAAGGAGGTTCAGATTGAGAAGAGATTTCGTTGATTTGATTGCTAGAAGGGAAACCCCTCGAAAGCAACTGTTCCTCGAGTCAGCTCAAACTCTCCAATCAACGTCTAAAAGTGTAAAGGAGAACATGGACAGTGGAGTCAAATCCGACGAAAGCAGTGAAAAAATACAGTCACTTGAGGTGCCCTGTGTCCCGATGTCCACTGAAGATGGGCAAGGCTTTGAAGGTTCAATTCCAATTGACTTGATAAATTCCTTCGAGCCAGAAGTAATCAGACTTCCGAAAAGGAAGAGAAAGAATGATTGTGTTTTTAAAGCCATCTCTGCATACCTCGGCATTGATGTTCAGGACTTCTTGAACTTTTTGGTAAATGAAGACATATCCGATGAGTTGCTTGAATGCATAGATGAAGATAAAGGCCTGTCGCACGAGATGATAGAGGAAGTTCTGGTGACGAAAGGGATTTCTATGGTCTACACTTCAGATTTCAAAGAAATGGCAGTTCTCAACAGGAAGTATGGGGTTAATGGAAAAATGTACTGCACAATCAGGGGGAATCATTGTGAGCTGAGTTCAAAGGAATGCTTCATCAGATTGCTGAAGGAGGGTGGTGAAGCACAGATGTCCAATGAGAATTTGAATGCTGACTCCTTGTTTGATCTTGGAAAGTTCGTGCACAACAGAGAAAGGGCCACAAAATTGGCAAAATCGATGGCTAGGGGCACAACCGGTCTTTTGACTGAATTTGATCCAGAATTTTGTAAAGGTATGGTCACACTTTCGGAAATGTTTCCGGAGAACTTCTCCTCTGTTGTCGGACTGAGGTTGGGTTTCGCTGGTTCCGGAAAAACCCATAAAGTTCTTCAGTGGATCAACTACACACCAAGCGTTAAAAGAATGTTTATTAGTCCGAGGAGAATGCTGGCCGACGAGGTTGAAATTCAATTAAGAGGGACATCTTGTCAAGTTCATACTTGGGAAACAGCTCTTAAAAAAATTGATGGAACCTTTATGGAGGTCTTTGTGGATGAAATTGGCCTTTACCCACCTGGATATCTCACTCTTCTACAAATGTGTGCTTTCCGAAGGATCGTTAAGGGGCAAAGTGAAAAGTTTTTAAGAGGAAAGCTTGCTGAACTTTCGAAGACCTGTTTGAATGTAAGGTGTTTTGGTGATCCATTGCAAATGAGGTATTATTCTGCAGAGGATACAAACCTTCTAGACAAGACACATGAACTTGATCTAATGGTCAAAACCATTAAGCACAAGTACCTTCTTCAAGGGTACAGGTTCGGCCAATGGTTCCAAGAGCTGATAAACATGCCAACTAGAGTGGACGGTTCGAAATTTTCAAGAAAATTTTTTGCGGACATTTCTAGTGTCAAAACCGAAGATTATGGACTTATACTTGTCGCCAAGAGGGAAGACAAGGGGACGTTTGCCGGGAGAATACCTGTGGCTACGGTCAGCGAATCACAGGGAATGACCATAAGCAAAAGGGTGCTAATATGTTTAGACCAGAATCTGTTTGCCGGGGGAGCCAATGCCGCCATAGTTGCGATCACTAGATCGAAAGTTGGGTTTGACTTTATTCTCAAAGGGAATTCACTAAAAGAGGTGCAGAAAATGGCCCAAAAAACTTTGTGGCAATTCGTGCTTGAAGGAAAGACCATACCGATGGAAAGAATTGTTAATATGAACCCCGGAGCCAGCTTTTATGAGAACCCCTTAGACGTCGGCAACTCATCCATTCAGGACAAAGCCTCACATGACTTGTTCATAATGCCTTTCATCAATTTAGCTGAAGAAGAAGTTGACCCTGAGGAAATTGTCGGAGATGTTATTGAACCAGTTGAGTGGTTCAAATGCCATATACCGGTTTTTGATACAGACCCGATGCTTGCAGAGATATTTGACAAAGTGGCAGCAAAGGAAAAGAGAGAATTTCAGTCAATGCTTGGGCTTTCAAATCAATTTCTTGACATGGAAAAAAATGGATGCAAAATCGATATTTTGCCTTTTGCTAGACAAAATGTTTTCCCTCACCATCAAGCTTCAGATGACGTCACCTTCTGGGCAGGTGTTCAGAAGAGGATAAGGAAGTCAAATTGGAGACGAGAAAAGTCGAAATTTGAGGAGTTTGAAGCTCAGGGGAAGGAATTGCTCAGTGAATTCATATCAATGTTGCCCTTTGAATTCAAGGTGAACATCAAGGACATTGAAGAAGGTGAAAAAAGCTTTCTTGAAAAGAGAAAGTTAAAGTCTGAAAAGATGTGGGCCAACCACTCAGAAAGATCTGACATTGACTGGAAACTTGACCATGCCTTTCTATTTATGAAATCTCAATATTGCACAAAGGAAGGCAAAATGTTCACTGAGGCAAAAGCTGGTCAAACCCTAGCATGTTTCCAACACATTGTTCTGTTTAGGTTTGGTCCCATGCTTCGAGCAATTGAAAGGGCTTTTCTTAGAAGTTGTGGAGAGTCATACTACATACATTCAGGAAAAAACTTTTTCTGCTTGGACAGCTTTGTGACAAAAAACGCAGAAGTCTTTGATGGGTTTTCTATTGAATCCGACTACACGGCTTTTGACTCATCTCAGGACCACGTGATTCTGGCCTTTGAAATGGCACTTTTGCAGTACCTAGGGGTGTCCAAAGAGTTTCAGTTGGATTATTTGAGATTGAAATTGACCCTTGGCTGCAGACTTGGATCTCTGGCCATTATGAGGTTCACTGGGGAATTCTGCACTTTTTTGTTCAATACATTTGCAAATATGCTTTTCACCCAGTTGAAATACAAAATTGACCCGAGGAAGCATAGGATTCTATTTGCTGGAGATGACATGTGCTCTCTAAGTTCTCTAAAAAGAAGAAAAGGGGAAAAGGCAACAAGACTCTTGAAGAGCTTTTCTTTAACTGCTGTGGAAGAGGTGCGGAAGTTTCCAATGTTCTGTGGGTGGTACTTGAGCCCATATGGGATTATCAAGTCTCCAAAGTTGTTGTGGGCCAGGATCAAAATGATGAGTGAAAGACAACTTTTAAAAGAATGTGTGGACAACTATCTGTTTGAAGCTATATTTGCCTACAGATTAGGTGAGAGGCTTTACACAATTTTGAAAGAAGAGGACTTTGAGTATCATTACCTTGTCATAAGGTTTTTTGTCAAAAATTCTAAGTTGCTGACAGGGTTGAGTAAAAGCCTCATTTTCGAAATTGGTGAAGGGATTGGGTCAGAATGGCAATCGTCAATGTCAACCATTTCCTCAAGGAAGTCGAGTCAACAGACCTCAAAATTGATGCCATCTCCTCCTCGGAATTGTACAAAGATGCCACTTTTTTTAAACCAGATGTGCTCAACTGTATCAAGCGATTTGAATCAAATGTCAAGGTGTCTTCAAGGTCAGGTGATGGGCTCGTGTTGTCTGATTTCAAACTACTTGATGACACAGAGATCGACTCCATTAGAAAGAAGAGCAACAAATACAAGTATCTGCATTATGGAGTCATCCTGGTCGGGATTAAGGCCATGCTACCGAACTTTAGGGGAATGGAGGGGAGGGTCATTGTGTATGACGGTGCATGCTTGGACCCAGAAAGAGGTCACATTTGTTCCTACTTGTTCAAATTTGAGTCCGACTGTTGTTACTTTGGGCTTAGACCGGAACATTGCCTATCCACAACCGATGCAAATTTGGCTAAAAGGTTCAGATTTAGAGTGGATTTTGATTGTCCACAATACGAGCAGGACACAGAGCTGTTTGCTCTCGACATTGGGGTTGCATACAGGTGTGTCAATTCAGCCAGATTCTTGGAAACTAAGACTGGTGATTCAGGATGGGCTTCACAGGCAATCAGCGGCTGTGAGGCACTTAAATTCAATGAAGAAATCAAGATGGCAATCCTGGATCACAAATCACCGCTGTTTCTGGAAGAAGGTGCACCAAACGTGCATATTGAAAAGAGAATGTTCAGGGGTGACAAAGTCAGAAGGTCACGCTCTATCTCTGCAAAAAGGGGGCCAAACTCAAGGCTACAGGAAAAGAGAGGATTTAGGTCCCTCTCAGCTAGAATCGAAAGATTTGGAGAAAATGAGTTTGGAAGACGTGCTTCAACAAGCGAGACGCCACCGGGTAGGAGTGTATCTGTGGAAGACACACATAGATCCGGCAAAGGAACTTCTGACGGTTCCTCCCCCTGAAGGATTCAAGGAAGGTGAAAGCTTTGAAGGCAGAGAGCTTTACCTTCTTCTCTGCAACCATTATTGTAAATATTTATTTGGTAATATTGCTGTTTTCGGGTCATCTGACAAGACCCAGTTTCCCGCTGTTGGATTTGATACCCCTCCGGTTCATTACAATCTAACAACGACCCCGAAAGAAGGGGAGACTGAAGAGCAAAGGAAGGCCAGAGAAGGTTCGTCTGGCGAAAAAACAAAAATTTGGAGAATCGACCTGTCAAATGTTGTACCCGAATTGAAAACTTTTGCTGCCACTTCCAGGCAGAACTCTTTGAACGAATGTACGTTCAGAAAGCTTTGTGAGCCATTTGCTGATTTGGCTCGGGAATTTCTTCATGAAAGGTGGTCAAGGGGACTTGCCACCAACATTTATAAGAAATGGCCCAAAGCTTTTGAAAAGAGCCCTTGGGTGGCTTTTGATTTTGCCACCGGTCTTAAAATGAACCGTCTAACACCTGATGAAAAACAGGTAATAGACAGAATGACTAAGAGGCTTTTTCGTACTGAAGGACAAAAAGGGGTTTTCGAGGCGGGTTCGGAGAGCAATCTGGAACTGGAGGGTTAGAAGTCGTGTGAAATTCCGCAAACTTGGTCGCGGTCTTGCAGGTTGACATGCCTGCCTTTATACTTAAATAAAGGGTTCCCCCGGTTTTCTGTGCATTTCCGGGTTAGTGTGGTTTTTCTAGAGTCTAGAGTTTG

1. **Apple stem grooving virus isolate Shaanxi-1, complete genome. Accession number: MK481977.1 (6495 bp).**

AAATTTAACAGGCTTAATTTCCGCGCTTTACGTCAATGGCTTTCACTTACAGAAATCCCCTCGAAATTGCAATCAACAAGCTTCCAAGTAAGCAGTCTGATCAACTGCTTTCTTTAACCACAGACGAGATTGAAAAGACATTAGAAGTGACCAATCGCTTCTTCTCTTTTTCTATCACTCCAGAAGATCAAGAATTGTTGACCAAGCATGGTCTAACACTTGCACCCATTGGATTTAAGTCACATTCCCATCCAATATCCAAAATGATAGAAAATCATCTTCTGTACATATGCATTCCAAGTCTTTTATCTTCTTTTAGATCCGTAGCCTTTTTCTCGCTTAGAGAAAATAAAGTCAATAATTTCCTTAAGATGCACTCAGTTTTTTCACATGGAAAAATTAAGTCTCTAGGCATGTACAACGCCATCATTGATGGAAAAGATAAATACAGGTATGGCGAAGTTTCTTTTTCTTCCTTTAGAGATAGGGTGATTGGTCTTAGGGATCAGTGCCTCATGCGTAATAAATTTCCAAAAGTTCTCTTTCTCCACGATGAACTTCATTTCCTAAGCCCTTTTGACGTTGCTTTTCTATTTGAGACAATCCCAGAAATTGATAGGGTCATAGCAACTACTGTTTTTCCGATTGAACTGCTTTTTGGGGACAAAGTTTCAAAGGAGCCCAGAGTTTACACCTACAAGGTCCATGGTTCCTCCTTTTCATTCTATCCAGATGGTGTAGCCTCTGAGTGCTATGAGCAAAATCTTGCAAACTCCAAATGGCCGTTCACTTGTAGTGGAATTCAGTGGGCTAACAGGAAAATAAGAGTTACTAAGTTGCAGAGTCTCTTTGCTCATCACGTATTTTCATTTGATAGAGGCAGGGCTTGCAACGAATTTAACCACTTTGACAAGCCGAGTTGTCTCCTTGCAGAAGAAATGCGCCTTTTGACCAAAAGGTTTGATAAGGCAGTCATTAACAGGAGTACGGTTTCTTCTTTGAGCACTTATATGGCTTGTCTCAAGACCGCCAATGCAGCTTCTGCTGTCGCCAAGCTGAGGCAACTAGAGAAAAGGGACCTTTATCCGGATGAACTAAACTTCGTTTATTCCTTTGGGGAGCATTTCAAAAATTTTGGAATGAGGGACGATTTTGATGTGTCAGTCCTGCAATGGGTGAAGGACAAATTCTGTCAAGTTATGCCTCACTTCATTGCCGCTAGTTTCTTTGAGCCAACTGAGTTCCATTTGAATATGCGAAAATTGTTGAATGACCTGGCCACTAAAGGGATAGAGGTTCCACTTTCAGTAGTGGTTTTAGATAAGGTAAATTTTATAGAGACCAGATTTCATGCCAGAATGTTCGAAATTGCCGAAGCTATTGGAGTCAGTTTAAGTCTACTTGGAAAAAGGTTTGACTATGAAGCTGAAAGTGAAGAATATTTTTCAGAAAATGGCTATCTTTTCATGCCTTCAAGGACAAATCCAAGTAGAAACTGGATCTTAAACTCTGGGTCTGTCAAAATTGATTTCCAGAAGTTGCAGAGGGCAAGGAGGTTCAGATTGAGAAGAGATTTCGTTGATTTGATTGCTAGAAGGGAAACCCCTCGAAAGCAACTGTTCCTCGAGTCAGCTCAAACTCTCCAATCAACGTCTAAAAGTGTAAAGGAGAACATGGACAGTGGAGTCAAATCCGACGAAAGCAGTGAAAAAATACAGTCACTTGAGGTGCCCTGTGTCCCGATGTCCACTGAAGATGGGCAAGGCTTTGAAGGTTCAATTCCAATTGACTTGATAAATTCCTTCGAGCCAGAAGTAATCAGACTTCCGAAAAGGAAGAGAAAGAATGATTGTGTTTTTAAAGCCATCTCTGCATACCTCGGCATTGATGTTCAGGACTTCTTGAACTTTTTGGTAAATGAAGACATATCCGATGAGTTGCTTGAATGCATAGATGAAGATAAAGGCCTGTCGCACGAGATGATAGAGGAAGTTCTGGTGACGAAAGGGATTTCTATGGTCTACACTTCAGATTTCAAAGAAATGGCAGTTCTCAACAGGAAGTATGGAATAAATGGAAAAATGTACTGCACAATCAGGGGGAATCATTGTGAGCTGAGTTCAAAGGAATGCTTCATCAGATTGCTGAAGGAGGGTGGTGAAGCACAGATGTCCAATGAGAATTTGAATGCTGACTCCTTGTTTGATCTTGGAAAGTTCGTGCACAACAGAGAAAGGGCCACAAAATTGGCAAAATCGATGGCTAGGGGCACAACCGGTCTTTTGACTGAATTTGATCCAGAATTTTGTAAAGGTATGGTCACACTTTCGGAAATGTTTCCGGAGAACTTCTCCTCTGTTGTCGGACTGAGGTTGGGTTTCGCTGGTTCCGGAAAAACCCATAAAGTTCTTCAGTGGATCAACTACACACCAAGCGTTAAAAGAATGTTTATTAGTCCGAGGAGAATGCTGGCCGACGAGGTTGAAATTCAATTAAGAGGGACATCTTGTCAAGTTCATACTTGGGAAACAGCTCTTAAAAAAATTGATGGAACCTTTATGGAGGTCTTTGTGGATGAAATTGGCCTTTACCCACCTGGATATCTCACTCTTCTACAAATGTGTGCTTTCCGAAGGATCGTTAAGGGGCAAAGTGAAAAGTTTTTAAGAGGAAGCTTGCTGAACTTGTCGAAGACCTGTTTGAATGTAAGGTGTTTTGGTGATCCATTGCAAATGAGGTATTATTCTGCAGAGGATACAAACCTTCTAGACAAGACACATGAACTTGATCTAATGGTCAAAACCATTAAGCACAAGTACCTTCTTCAAGGGTACAGGTTCGGCCAATGGTTCCAAGAGCTGATAAACATGCCAACTAGAGTGGACGGTTCGAAATTTTCAAGAAAATTTTTTGCGGACATTTCTAGTGTCAAAACCGAAGATTATGGACTTATACTTGTCGCCAAGAGGGAAGACAAGGGGACGTTTGCCGGGAGAATACCTGTGGCTACGGTCAGCGAATCACAGGGAATGACCATAAGCAAAAGGGTGCTAATATGTTTAGACCAGAATCTGTTTGCCGGGGGAGCCAATGCCGCCATAGTTGCGATCACTAGATCGAAAGTTGGGTTTGACTTTATTCTCAAAGGGAATTCACTAAAAGAGGTGCAGAAAATGGCCCAAAAAACTTTGTGGCAATTCGTGCTTGAAGGAAAGACCATACCGATGGAAAGAATTGTTAATATGAACCCCGGAGCCAGCTTTTATGAGAACCCCTTAGACGTCGGCAACTCATCCATTCAGGACAAAGCCTCACATGACTTGTTCATAATGCCTTTCATCAATTTAGCTGAAGAAGAAGTTGACCCTGAGGAAATTGTCGGAGATGTTATTGAACCAGTTGAGTGGTTCAAATGCCATATACCGGTTTTTGATACAGACCCGATGCTTGCAGAGATATTTGACAAAGTGGCAGCAAAGGAAAAGAGAGAATTTCAGTCAATGCTTGGGCTTTCAAATCAATTTCTTGACATGGAAAAAAATGGATGCAAAATCGATATTTTGCCTTTTGCTAGACAAAATGTTTTCCCTCACCATCAAGCTTCAGATGACGTCACCTTCTGGGCAGGTGTTCAGAAGAGGATAAGGAAGTCAAATTGGAGACGAGAAAAGTCGAAATTTGAGGAGTTTGAAGCTCAGGGGAAGGAATTGCTCAGTGAATTCATATCAATGTTGCCCTTTGAATTCAAGGTGAACATCAAGGACATTGAAGAAGGTGAAAAAAGCTTTCTTGAAAAGAGAAAGTTAAAGTCTGAAAAGATGTGGGCCAACCACTCAGAAAGATCTGACATTGACTGGAAACTTGACCATGCCTTTCTATTTATGAAATCTCAATATTGCACAAAGGAAGGCAAAATGTTCACTGAGGCAAAAGCTGGTCAAACCCTAGCATGTTTCCAACACATTGTTCTGTTTAGGTTTGGTCCCATGCTTCGAGCAATTGAAAGGGCTTTTCTTAGAAGTTGTGGAGAGTCATACTACATACATTCAGGAAAAAACTTTTTCTGCTTGGACAGCTTTGTGACAAAAAACGCAGAAGTCTTTGATGGGTTTTCTATTGAATCCGACTACACGGCTTTTGACTCATCTCAGGACCACGTGATTCTGGCCTTTGAAATGGCACTTTTGCAGTACCTAGGGGTGTCCAAAGAGTTTCAGTTGGATTATTTGAGATTGAAATTGACCCTTGGCTGCAGACTTGGATCTCTGGCCATTATGAGGTTCACTGGGGAATTCTGCACTTTTTTGTTCAATACATTTGCAAATATGCTTTTCACCCAGTTGAAATACAAAATTGACCCGAGGAAGCATAGGATTCTATTTGCTGGAGATGACATGTGCTCTCTAAGTTCTCTCAAAAGAAGAAAAGGGGAAAAGGCAACAAGACTCTTGAAGAGCTTTTCTTTAACTGCTGTGGAAGAGGTGCGGAAGTTTCCAATGTTCTGTGGGTGGTACTTGAGCCCATATGGGATTATCAAGTCTCCAAAGTTGTTGTGGGCCAGGATCAAAATGATGAGTGAAAGACAACTTTTAAAAGAATGTGTGGACAACTATCTGTTTGAAGCTATATTTGCCTACAGATTAGGTGAGAGGCTTTACACAATTTTGAAAGAAGAGGACTTTGAGTATCATTACCTTGTCATAAGGTTTTTTGTCAAAAATTCTAAGTTGCTGACAGGGTTGAGTAAAAGCCTCATTTTCGAAATTGGTGAAGGGATTGGGTCAGAATGGCAATCGTCAATGTCAACCATTTCCTCAAGGAAGTCGAGTCAACAGACCTCAAAATTGATGCCATCTCCTCCTCGGAATTGTACAAAGATGCCACTTTTTTTAAACCAGATGTGCTCAACTGTATCAAGCGATTTGAATCAAATGTCAAGGTGTCTTCAAGGTCAGGTGATGGGCTCGTGTTGTCTGATTTCAAACTACTTGATGACACAGAGATCGACTCCATTAGAAAGAAGAGCAACAAATACAAGTATCTGCATTATGGAGTCATCCTGGTCGGGATTAAGGCCATGCTACCGAACTTTAGGGGAATGGAGGGGAGGGTCATTGTGTATGACGGTGCATGCTTGGACCCAGAAAGAGGTCACATTTGTTCCTACTTGTTCAAATTTGAGTCCGACTGTTGTTACTTTGGGCTTAGACCGGAACATTGCCTATCCACAACCGATGCAAATTTGGCTAAAAGGTTCAGATTTAGAGTGGATTTTGATTGTCCACAATACGAGCAGGACACAGAGCTGTTTGCTCTCGACATTGGGGTTGCATACAGGTGTGTCAATTCAGCCAGATTCTTGGAAACTAAGACTGGTGATTCAGGATGGGCTTCACAGGCAATCAGCGGCTGTGAGGCACTTAAATTCAATGAAGAAATCAAGATGGCAATCCTGGATCACAAATCACCGCTGTTTCTGGAAGAAGGTGCACCAAACGTGCATATTGAAAAGAGAATGTTCAGGGGTGACAAAGTCAGAAGGTCACGCTCTATCTCTGCAAAAAGGGGGCCAAACTCAAGGCTACAGGAAAAGAGAGGATTTAGGTCCCTCTCAGCTAGAATCGAAAGATTTGGAGAAAATGAGTTTGGAAGACGTGCTTCAACAAGCGAGACGCCACCGGGTAGGAGTGTATCTGTGGAAGACACACATAGATCCGGCAAAGGAACTTCTGACGGTTCCTCCCCCTGAAGGATTCAAGGAAGGTGAAAGCTTTGAAGGCAGAGAGCTTTACCTTCTTCTCTGCAACCATTATTGTAAATATTTATTTGGTAATATTGCTGTTTTCGGGTCATCTGACAAGACCCAGTTTCCCGCTGTTGGATTTGATACCCCTCCGGTTCATTACAATCTAACAACGACCCCGAAAGAAGGGGAGACTGAAGAGCAAAGGAAGGCCAGAGAAGGTTCGTCTGGCGAAAAAACAAAAATTTGGAGAATCGACCTGTCAAATGTTGTACCCGAATTGAAAACTTTTGCTGCCACTTCCAGGCAGAACTCTTTGAACGAATGTACGTTCAGAAAGCTTTGTGAGCCATTTGCTGATTTGGCTCGGGAATTTCTTCATGAAAGGTGGTCAAGGGGACTTGCCACCAACATTTATAAGAAATGGCCCAAAGCTTTTGAAAAGAGCCCTTGGGTGGCTTTTGATTTTGCCACCGGTCTTAAAATGAACCGTCTAACACCTGATGAAAAACAGGTAATAGACAGAATGACTAAGAGGCTTTTTCGTACTGAAGGACAAAAAGGGGTTTTCGAGGCGGGTTCGGAGAGCAATCTGGAACTGGAGGGTTAGAAGTCGTGTGAAATTCCGCAAACTTGGTCGCGGTCTTGCAGGTTGACATGCCTGCCTTTATACTTAAATAAAGGGTTCCCCCGGTTTTCTGTGCATTTCCGGGTTAGTGTGGTTTTTCTAGAGTCTAGAGTTTGTCCACTCT

1. **Apple stem grooving virus isolate Fuji-BJ, complete genome. Accession number: OP535345.1 (6487 bp).**

CAGCGCTTAATTTCCGCGCTTTACGTCAATGGCTTTCACTTACAGAAATCCCCTCGAAATTGCAATCAATAAGCTTCCAAGCAGGCAGTCTGATCAACTGCTTTCTTTAACCACAGACGAGATTGAAAAGACCTTAGAAGTGACTAATCGCTTCTTCTCTTTTTCTATCACTCCAGAAGATCAAGAATTGTTGACCAAGCATGGTCTAACACTTGCACCTATTGGATTTAAGTCACATTCCCATCCAATATCCAAAATGATAGAAAATCACCTTCTGTACATATGCATTCCAAGTCTTTTATCTTCTTTTAGATCCGTAGCCTTTTTCTCGCTTAGAGAAAATAAAGTCAATAATTTTCTTAAGATGCACTCAGTTTTTTCACATGGAAAAATTAAGTCTCTAGGCATGTACAACGCCATCATTGATGGAAAAGATAAATACAGGTATGGCGAAGTTTCCTTTTCTTCCTTTAGAGACAGGGTGATTGGTCTCAGGGATCAGTGCCTCATGCGTGATAAATTTCCAAAAGTTCTCTTTCTCCACGATGAACTTCATTTCCTAAGCCCTTTCGACGTTGCTTTTCTATTTGAGACAATCCCAGAGATTGATAGAGTCATAGCAACTACTGTTTTTCCGATTGAACTGCTTTTTGGGGACAAGGTTTCAAAGGAGCCCAGAGTTTACACCTACAAGGTCCATGGTTCCTCCTTTTCATTCTATCCAGATGGTGTAGCCTCCGAGTGCTATGAGCAAAATCTTGCAAACTCCAAATGGCCGTTCACTTGTAGTGGAATTCAGTGGGCTAACAGGAAAATAAGGGTTACTAAGCTGCAGAGCCTCTTTGCTCATCACGTATTTTCATTTGATAGAGGCAGGGCTTGCAACGAATTTAACCACTTTGACAAGCCGAGTTGTCTCCTTGCAGAAGAAATGCGCCTTTTGACCAAAAGGTTTGATAAGGCAGTCATTAATAGGAGTACGGTTTCTTCTTTGAGCACTTATATGGCTTGTCTCAAGACCGCCAATGCAGCTTCTGCTGTCGCCAAGCTGAGGCAACTAGAGAAAAGGGACCTCTATCCGGATGAACTAAACTTTGTTTATTCCTTTGGGGAACATTTCAAAAATTTTGGAATGAGGGACGATTTTGATGTGTCAGTCCTGCAATGGGTGAAGGACAAATTCTGTCAAGTTATGCCTCACTTCATTGCTGCCAGTTTCTTTGAGCCAACTGAGTTCCATTTGAATATGCGAAAATTGTTGAATGACCTGGCCACTAAAGGGATAGAGGTTCCACTTTCAGTAGTGGTTTTAGATAAGGTAAATTTTATAGAGACCAGATTTCATGCCAGAATGTTCGAAATTGCCGAAGCTATTGGAGTCAGTTTAAGTCTACTTGGAAAACGGTTTGACTATGAAGCTGAAAGTGAAGAATATTTTTCAGAAAATGGCTATCTCTTCATGCCTTCAAGGACAAATCCAAGTAGAAACTGGATCTTAAACTCTGGGTCTGTCAAAATTGATTTCCAGAAGTTGCAGAGGGCAAGGAGGTTCAGACTGAGAAGAGATTTCGTTGATTTGATTGCTAGAAGAGAAACCCCTCGAAAGCAACTATTTCTCGAGTCAGCTCAAACTCTCCAATCAACGTCTAAAAGTGTGGAGGAGAACATGGACAGTGGAGTCAGATCCGACGAAAGCAGTAAAGAAATACAGTCACTCGAGGTGCCCTGTGTCCCGATGTCCACTGAAGATGGGCAAGGCTTTGAAGGTTCAATTCCAATTGACTTGATAAATTCCTTCGAGCCAGAAGTAATCAGACTTCCGAAAAGGAAGAGAAAGAATGATTGTGTTTTTAAAGCCATCTCTGCATACCTCGGCATTGATGTTCAGGACCTCTTGAACTCTTTGGTGAATGAAGATATATCCGATGAGTTGCTTGAATGCATAGATGAAGATAAAGGCCTGTCGCACGAGATGATAGAGGAAGTTCTGGTAACGAGAGGGATTTCTATGGTCTACACTTCAGATTTCAAAGAAATGGCAGTTCTCAACAGGAAGTATGGAGTTAATGGAAAAATGTACTGCACAATCAGGGGGAATCATTGTGAGCTGAGTTCAAAGGAATGCTTCATCAGATTGCTGAAGGAGGGTGGTGAAGCACAAATGTCCAATGAGAATTTGAATGCTGACTCCTTGTTTGATCTTGGAAAGTTCGTGCACAACAGAGAAAGGGCCACAAAATTGGCAAAATCGATGGCTAGGGGCACAACCGGTCTTTTGACTGAATTTGATCCAGAATTTTGTAAAGGTATGGTCACGCTTTCGGAAATGTTTCCGGAGAACTTCTCCTCTGTTGTCGGACTGAGGTTGGGCTTCGCTGGTTCCGGAAAAACCTATAAAGTTCTTAAATGGATCAACTACACACCAAGCGTCAAAAGAATGTTTATTAGTCCAAGGAGAATGCTGGCCGACGAGGTTGAAATTCAATTAAGAGGGACATCTTGTCAAGTTCATACTTGGGAAACAGCTCTTAAAAAAATTGATGCAACCTTCATGGAGGTCTTTGTGGATGAAATTGGCCTTTGCCCACCTGGATATCTCACTCTTCTACAAATGTGTGCTTTCCGAAGGACCGTTAAGTGGCAAAGTGAAAAGTTTTTAAAAGGAAAGCTTGCTGAACTTTCGAAGACCTGTTTGAATATAAGGTGTTTTGGTGACCCATTGCAAATGAGGTATTATTCTGCAGAGGATACAAACCTTCTAGACAAGACACATGAGATTGATCTAATGGTCAAAACTATCAAACACAAGTACCTTTTTCAAGGGTATAGGTTCGGTCAGTGGTTCCAAGAGCTGATAAACATGCCAACTAGAGTGGACGATTCGAAATTTTCAAGGAAATTTTTTGCGGACATTTCTAGTGTCAAAACCGAAGATTATGGACTCATACTTGTCGCCAGGAGGGAAGACAGGGGGACGTTTGCCGGGAGGATACCTGTGGCCACGGTCAGCGAATCACAGGGAATGACCATAAGCAAGAGGGTGCTAATATGCTTAGACCAAAATCTGTTTGCCGGGGGAGCCAATGCCGCCATGGTTGCGATCACTAGATCGAAAGTTGGGTTTGACTTTATTCTCAAAGGGAATTCACTAAAAGAGGTGCAGAAAATGGCCCAAAAAACTTTGTGGCAATTCGTACTTGAAGGAAAGACCATACCGATGGAAAGAATTGTCAATATGAACCCCGGAGCCAGCTTTTATGAGAGCCCCTTGGACGTTGGCAACTCGTCCATTCAGGACAAAGCCTCACATGACTTGTTCATAATGCCTTTTATCAATTTAGCTGAAGAAGAAGTTGACCCTGAGGAAGTCGTCGGAGATGTTGTTGAACCAGTTGAGTGGTTCAAGTGCCATATACCGGTTTTTGATACAGACCCGATGCTTGCAGAGATATTTGACAAAGTGGCAGCAAAGGAAAAGAGAGAATTCCAGTCCATACTTGGGCTTTCAAATCAATTTCTTGACATGGAAAAGAATGGATGCAAAATTGATATTTTGCCTTTTGCTAGACAGAATGTTTTCCCTCACCATCAAGCTTCAGATGACGTCACCTTTTGGGCAGGTGTTCAGAAGAGAATAAGGAAGTCAAATTGGAGACGGGAAAAGTCAAAATTTGAGGAGTTTGAAGCTCAGGGGAAGGAATTGCTCAGTGAATTCATGTCAATGTTGCCCTTTGAATTCAAGGTGAACATCAAGGACATTGAAGAAGGTGAAAAAAGCTTTCTTGAAAAGAGAAAGTTAAAGTCTGAAAAGATGTGGGCCAACCACTCAGAAAGATCTGACATTGACTGGAAACTTGACCACGCCTTTCTATTTATGAAATCTCAATATTGCACAAAGGAAGGCAAAATGTTCACTGAGGCAAAAGCTGGTCAAACCTTAGCATGTTTCCAACACATTGTTCTGTTCAGATTTGGTCCCATGCTTCGAGCAATTGAAAGGGCTTTTCTTAGAAGTTGTGGAGAGTCATACTACATACATTCAGGAAAAAACTTTTTCTGCTTGGACAGCTTTGTGACAAAAAACGCAGAAGTCTTTGATGGGTTTTCTATTGAATCTGACTACACGGCTTTTGACTCATCTCAGGATCACGTAATTTTGGCCTTTGAAATGGCACTTTTGCAGTACCTAGGGGTGTCCAAAGAGTTTCAGTTGGATTATTTGAGATTGAAATTGACCCTTGGCTGCAGACTTGGATCTCTAGCCATTATGAGGTTCACTGGGGAATTTTGCACTTTTTTGTTCAATACATTTGCAAACATGCTTTTCACCCAGTTGAAATACAAAATTGACCCGAGGAAGCATAGGATTCTATTTGCTGGAGATGACATGTGCTCTCTAAGTTCTCTCAAAAGAAGAAAAGGGGAAAAGGCAACAAGACTGTTGAAGAGCTTTTCTTTAACTGCTGTGGAAGAGGTACGGAAGTTTCCAATGTTCTGTGGGTGGTACTTGAGCCCATATGGGATTATCAAGTCTCCAAAGTTGTTGTGGGCCAGGATCAAAATGATGAGTGAAAGACAACTTTTAAAGGAATGTGTGGACAACTATCTGTTTGAAGCTATATTTGCCTACAGATTAGGTGAGAGGCTTTACACAATTTTGAAAGAAGAGGACTTTGAGTATCATTACCTTGTTATAAGGTTTTTCGTCAAAAATTCTAAGTTGCTGACAGGGTTGAGCAAAAGCCTCATTTTCGAGATTGGTGAAGGGATTGGGTCAGAATGGCAATCGTCAATGTCAACCATTTCCTCAAGGAAGTCGAGTCAACAGATCTCAAAATTGATGCCATCTCTTCCTCGGAATTGTACAAAGATGCCACTTTTTTTAAACCGGATGTGCTCAACTGTATCAAGCGATTTGAATCAAATATCAAGGTGTCTTCAAGGTCAGGTGATGGGCTCGTGTTGTCTGATTTCAAACTACTTGATGACACAGAGATCGACTCCATTAGAAAGAAGAGCAACAAATACAAGTATCTGCATTATGGAGTCATTCTGGTCGGGATCAAGGCTATGCTACCGAACTTTAGGGGAATGGAAGGGAGGGTCATTGTATATGACGGTGCATGCTTGGACCCAGAAAGAGGTCACATTTGTTCCTACTTGTTCAAATTTGAGTCCGACTGTTGTTACTTTGGACTTAGACCGGAACATTGCCTATCCACAACCGATGCAAATTTGGCTAAAAGGTTCAGATTTAGAGTGGATTTTGATTGTCCACAATACGAGCAGGACACAGAGCTGTTTGCTCTCGACATTGGGGTTGCATACAGGTGTGTCAATTCAGCCAGATTCTTGGAAACTAAGACTGGTGATTCAGGATGGGCTTCACAGGCAATCAGCGGCTGTGAGGCACTTAAATTTAATGAAGAAATCAAGATGGCAATCCTGGATCACAGATCACCGCTGTTTCTGGAAGAAGGTGCACCAAACGTGCATATTGAGAAGAGAATGTTTAGAGGTGACAAAGTCAGAAGGTCACGCTCTATCTCTGCAAAAAGGGGGCCAAACTCAAGGTTACAAGAAAAGAGAGGATTTAGGTCCCTCTCAGCTAGAATCGAAAGATTTGGAGAGAATGAGTTTGGAAGACGTGCTTCAACAAGCGAGGCGCCACCGGGTAGGAGTGTATCTGTGGAAGACACACATAGATCCGGCAAAGGAACTTCTGACGGTTCCTCCCCCTGAAGGATTCAAGGAAGGTGAAAGCTTTGAAGGCAGAGAGCTTTACCTTCTTCTCTGCAACCACTATTGTAAATATTTATTTGGTAATATTGCTGTTTTCGGGTCATCTGACAAGACCCAGTTTCCCGCTGTTGGATTTGATACCCCTCCGGTTCATTACAATCTGACAACGACCCCGAAAGAAGGGGAGACTGAAGAGCAAAGGAAAGCCAGAGAAGGTTCGTCTGGCGAAAAAACAAAAATTTGGAGAATCGACCTGTCAAATGTTGTACCCGAATTGAAAACTTTTGCTGCCACTTCCAGGCAGAACTCTTTGAACGAATGTACGTTCAGAAAGCTTTGTGAGCCATTTGCTGATTTGGCTCGGGAATTTCTTCATGAAAGATGGTCAAGGGGACTTGCCACCAACATTTACAAGAAATGGCCCAAAGCTTTTGAAAAGAGCCCTTGGGTGGCTTTTGATTTTGCCACCGGTCTTAAAATGAACCGTCTAACACCTGATGAAAAACAGGTAATAGACAGAATGACTAAGAGGCTTTTTCGTACTGAAGGACAAAAGGGGGTTTTCGAAGCGGGTTCGGAGAGCAATCTGGAACTGGAGGGTTAGAAGTCGTGTGAAATTCCGCAAACTTGGTCGCGGTCTTGCAGGTTGACATGCCTGCCTTTATACTTAAATAAAGGGTTCCCCCGGTTTTCTGTGCATTTCCGGGTTAGTGTGGTTTTTCTAGAGTCTAGAGTTTGTCCCCTC

1. **Apple stem grooving virus isolate AC, complete genome. Accession number: JX080201.1 (6496 bp).**

AAATTTAACAGCGCTTAATTTCCGCGCTTTACGTCAATGGCTTTCACTTACAGAAATCCCCTCGAAATTGCAATCAACAAGCTTCCAAGTAAGCAGTCTGATCAACTGCTTTCTTTAACCACAGACGAGATTGAAAAGACCTTAGAAGTGACTAATCGCTTCTTCTCTTTTTCTATCACTCCAGAAGATCAAGAATTGTTGACCAAGCATGGTCTAACACTTGCACCCATTGGATTTAAGTCACATTCCCATCCAATATCCAAAATGATAGAAAATCACCTTCTGTACATATGCATTCCAAGTCTTTTATCTTCTTTTAGATCCGTAGCCTTTTTCTCGCTTAGAGAAAATAAAGTCAATAATTTTCTTAAGATGCACTCAGTTTTTTCACATGGAAAAATTAAGTCTCTAGGCATGTACAACGCCATCATTGATGGAAAAGATAAATACAGGTATGGCGAAGTTTCTTTTTCTTCCTTTAGAGATAGGGTGATTGGTCTCAGGGATCAATGCCTCATGCGTAATAAATTTCCAAAAGTTCTCTTTCTCCACGATGAACTTCATTTCCTAAGCCCTTTTGACGTTGCTTTTCTATTTGAGACAATCCCAGAAATTGATAGAGTCATAGCAACTACTGTTTTTCCGATTGAACTGCTTTTTGGGGACAAGGTTTCAAAGGAGCCCAGAGTTTACACCTACAAGGTCCATGGTTCCTCCTTTTCATTCTATCCAGATGGTGTAGCCTCTGAGTGCTATGAGCAAAATCTTGCAAACTCTAAATGGCCGTTCACTTGTAGTGGAATCCAGTGGGCTAACAGGAAAATAAGGGTTACTAAGTTGCAGAGCCTCTTTGCTCATCACGTATTTTCATTTGATAGAGGCAGGGCTTGCAACGAATTTAACCACTTTGACAAGCCGAGTTGTCTCCTTGCAGAAGAAATGCGCCTTTTGACCAAAAGGTTTGATAAGGCAGTCATTAATAGGAGTACGGTTTCTTCTTTGAGCACTTATATGGCTTGTCTCAAGACCGCCAATGCAGCTTCTGCTGTCGCCAAGCTGAGGCAACTAGAGAAAAGGGACCTCTATCCGGATGAACTAAACTTCGTTTATTCCTTTGGGGAGCATTTCAAAAATTTTGGAATGAGGGACGATTTTGATGTGTCAGTCCTACAATGGGTGAAAGACAAATTCTGTCAAGTTATGCCTCACTTCATTGCTGCCAGTTTCTTTGAGCCAACTGAGTTCCATTTGAATATGCGAAAATTGTTGAATGACCTGGCCACTAAAGGGATAGAGGTTCCACTTTCAGTAGTGGTTTTGGATAAGGTAAATTTTATAGAGACCAGATTTCATGCCAGGATGTTCGAAATTGCCGAAGCTATTGGAGTCAGTTTAAGTCTACTTGGAAAAAGGTTTGACTATGAAGCTGAAAGTGAAGAATATTTTTCAGAAAATGGCTATCTTTTTATGCCTTCAAGGACAAATCCAAGTAGAAACTGGATCTTAAACTCTGGGTCTGTCAAAGTTGATTTCCAGAAGTTGCAGAGGGCAAGGAGGTTCAGATTGAGAAGAGATTTCGTTGATTTGATCGCTAGAAGGGAAACCCCTCGAAAGCAACTATTCCTCGAGTCAGCTCAAACTCTCCAATCAACGTCTAAAAGTGTGGAGGAGAACATGGACAGTGGAGTCAAATCCGACGAAAGCAGTAAAAAAATACTGTCACTCGAGGTGCCCTGTGTCCCGATGTCCACTGAAGATGGGCAAGGCTTTGAAGGTTCAATTCCAATTGACTTGATAAATTCCTTCGAGCCAGAAGCAATCAGACTTCCGAAAAGGAAGAGAAAGAATGATTGTGTTTTCAAAGCCATCTCTGCACACCTCGGCATTGATGTTCAGGACCTCTTGAACTTTTTGGTAAATGAAGACATATCCGATGAGTTGCTTGAATGCATAGATGAAGATAAAGGCCTGTCGCACGAGATGATAGAGGAAGTTCTGGTGACGAAAGGGATTTCTATGGTCTACACTTCAGATTTCAAAGAGATGGCAGTTCTCAACAGGAAGTATGGAGTTAATGGAAAAATGTACTGCACAATCAGGGGGAATCATTGTGAGCTGAGTTCGAAGGAATGCTTCATCAGATTGCTGAAGGAGGGTGGTGAAGCACAAATGTCCAATGAGAATTTGAATGCTGACTCCTTGTTTGATCTTGGAAAGTTCGTGCACAACAGAGAAAGGGCCACAAAATTGGCAAAATCGATGGCTAGGGGCACAACCGGTCTTTTGACTGAGTTTGATCCAGAATTTTGCAAAGGTATGGTCACACTTTCGGAAATGTTTCCGGAGAACTTCTCCTCTGTTGTAGGACTGAGGTTGGGCTTCGCTGGTTCCGGAAAAACCCATAAAGTTCTTCAATGGATCAACTACACACCAAGCGTCAAAAGAATGTTTATTAGTCCAAGGAGAATGCTGGCCGACGAGGTTGAAATTCAATTAAGAGGGACATCTTGTCAAGTTCATACTTGGGAAACAGCTCTTAAAAAGATTGATGGAACCTTCATGGAGGTCTTTGTGGATGAAATTGGCCTTTACCCACCTGGATATCTCACTCTTCTACAAATGTGTGCTTTCCGAAAGATCGTTAAGGGGCAAAGTGAAAAGTTTTTAAGAGGAAAGCTTGCTGAACTTTCGAAGACCTGTTTGAATATAAGGTGTTTTGGTGACCCATTGCAAATGAGGTATTATTCTGCAGAGGATACAAACCTTCTAGACAAGACACATGAGCTCGATCTAATGGTCAAAACCATCAAACACAAGTACCTTCTTCAAGGGTATAGGTTCGGCCAATGGTTCCAAGAGCTGATAAACATGCCAACTAGAGTGGACGATTCGAAATTTTCAAGGAAATTTTTTGCAGACATTTCTAGTGTCAAAACCGAAGATTATGGACTCATACTTGTCGCCAAGAGGGAAGACAAGGGGACGTTTGCCGGGAGAATACCTGTGGCTACGGTCAGCGAATCACAGGGAATGACCATAAGCAAGAGGGTGCTAATATGCTTAGACCAAAATATATTTGCCGGGGGAGCCAATGCCGCCATAGTTGCGATCACTAGATCGAAAGTTGGGTTTGACTTTATTCTCAAAGGGAATTCACTAAAAGAGGTGCAGAAAATGGCCCAAAAAACTTTGTGGCAATTCGTACTTGAAGGAAAGACCATACCGATGGAAAGAATTGTCAATATGAACCCCGGAGCCAGCTTTTATGAGAGCCCCTTAGACGTTGGCAACTCGTCCATTCAGGACAAAGCCTCACATGACTTGTTCATAATGCCTTTTATCAATTTAGCTGAAGAAGAAGTTGACCCTGAGGAAATCGTCGGAGATGTTATTGAACCAGTTGAGTGGTTCAAATGCCATATACCGGTTTTTGATACAGACCCGATGCTTGCGGAGATATTTGACAAAGTGGCAGCAAAGGAAAAGAGAGAATTTCAGTCCATACTTGGGCTTTCAAATCAATTTCTTGACATGGAAAAAAATGGATGCAGAATCGATATTTTGCCTTTTGCTAGACAGAATGTTTTCCCTCACCATCAAGCTTCAGATGACGTCACCTTTTGGGCAGGTGTTCAGAAGAGGATAAGGAAGTCAAATTGGAGACGGGAAAAGTCAAAATTTGAGGAGTTTGAAGCTCAGGGGAAAGAATTGCTCAGTGAATTCATATCAATGTTGCCCTTTGAATTCAAGGTAAACATCAAGGACGTTGAAGAAGGTGAAAAAAGCTTTCTTGAAAAAAGAAAGTTAAAGTCTGAAAAGATGTGGGCCAACCACTCAGAAAGATCTGACATTGACTGGAAACTTGACCATGCCTTTCTATTTATGAAATCTCAATATTGCACAAAGGAAGGCAAAATGTTCACTGAGGCAAAAGCTGGTCAAACCTTAGCATGTTTCCAACACATTGTTCTGTTTAGGTTTGGTCCCATGCTTCGAGCAATTGAAAGGGCTTTTCTTAGAAGTTGTGGAGAGTCATACTACATACATTCAGGGAAAAACTTTTTCTGCTTGGACAGCTTTGTGACAAAAAACGCAGAAGTCTTTGATGGGTTTTCTATTGAATCTGACTACACGGCTTTTGACTCATCTCAGGACCACGTAATTTTGGCCTTTGAAATGGCACTTTTGCAGTACCTAGGGGTGTCCAAAGAGTTTCAGTTGGATTATTTGAGATTGAAATTGACCCTTGGCTGCAGACTTGGATCTCTAGCCATTATGAGGTTCACTGGGGAATTCTGCACTTTTTTATTCAATACATTTGCAAACATGCTTTTCACTCAGTTGAAATACAAAATTGACCCGAGGAAGCATAGGATTCTATTTGCTGGAGATGACATGTGCTCTCTAAGTTCTCTCAAAAGAAGAAAAGGGGAAAAGGCAACAAGACTGCTGAAGAGCTTTTCTTTAACTGCTGTGGAAGAGGTACGGAAGTTTCCAATGTTCTGTGGGTGGTACTTGAGCCCATATGGGATTATCAAGTCTCCAAAGTTGTTGTGGGCCAGGATCAAGATGATGAGTGAAAGACAACTTTTAAAGGAATGTGTGGATAACTATCTGTTTGAAGCTATATTTGCCTACAGATTAGGTGAGAGGCTTTACACAATTTTGAAAGAAGAGGACTTTGAGTATCATTACCTTGTCATAAGGTTTTTCGTTAAAAATTCTAAGTTGCTGACAGGGTTGAGCAAAAGCCTCATTTTCGAAATTGGTGAAGGGATTGGGTCAGAATGGCAATCGTCAATGTCAACCATTTCCTCAAGGAAGTCGAATCAACGGATCTCAAAATTGATGCCATCTCTTCCTCGGAATTGTACAAAGATGCCACTTTTTTCAAACCAGATGTGCTCAACTGTATCAAGCGATTTGAATCAAATGTCAAGGTGTCTTCAAGGTCAGGTGATGGGCTCGTACTGTCTGATTTCAAACTGCTTGATGACACAGAGATCGACTCCATTAGAAAGAAGAGCAACAAATACAAGTATCTGCATTATGGAGTCATTCTGGTCGGGATTAAGGCTATGCTACCGAACTTTAGGGGAATGGAGGGGAGGGTCATTGTGTATGACGGTGCATGCTTGGACCCAGAAAGAGGTCACATTTGTTCCTACTTATTCAAATTTGAGTCCGACTGTTGTTACTTTGGGCTTAGACCGGAACATTGCCTATCCACAACCGATGCAAATTTGGCTAAAAGGTTCAGATTTAGAGTGGATTTTGATTGTCCACAATACGAGCAGGACACAGAGCTGTTTGCTCTCGACATTGGGGTTGCATACAGGTGTGTCAATTCAGCCAGGTTCTTGGAAACTAAGACTGGTGATTCAGGATGGGCTTCACAGGCGATCAGCGGCTGTGAGGCACTTAAATTCAATGAAGAAATCAAGATGGCAATCCTGGATCACAAATCACCGCTGTTTCTGGAAGAAGGTGCACCAAACGTGCATATTGAAAAGAGAATGTTTAGGGGTGACAAAGTCAGAAGGTCACGCTCTATCTCTGCAAAAAGGGGGCCAAACTCAAGGCTACAGGAAAAGAGAGGATTTAGGTCCCTCTCAGCTAGAATCGAAAGATTTGGAGAAAATGAGTTTGGAAGACGTGCTTCAACAAGCGAGACGCCACCGGGTAGGAGTGTATCTATGGAAGACACACATAGATCCGGCAAAGGAACTTCTGACGGTTCCTCCCCCTGAAGGATTCAAGGAAGGTGAAAGCTTTGAAGGCAGAGAGCTTTACCTTCTTCTCTGCAACCATTATTGTAAATATTTATTTGGTAATATTGCTGTTTTCGGGTCATCTGACAAGACCCAGTTTCCCGCTGTTGGATTTGATACCCCTCCGGTTCATTACAATCTAACAACGACCCCGAAAGAAGGGGAGACTGAAGAGCAAAGGAAGGCCAGAGAAGGTTCGTCTGGCGAAAAAACAAAAATTTGGAGAATCGACCTGTCAAATGTTGTACCCGAATTGAAAACTTTTGCTGCCACTTCCAGGCAGAACTCTTTGAACGAATGTACGTTCAGAAAGCTTTGTGAGCCATTTGCTGATTTGGCTCGGGAATTTCTTCATGAAAGATGGTCAAGGGGACTTGCCACCAACATTTATAAGAAATGGCCCAAAGCTTTTGAAAAAAGCCCTTGGGTGGCTTTTGATTTTGCCACCGGTCTTAAAATGAACCGTCTAACGCCTGATGAAAAACAGGTAATAGACAGAATGACTAAGAGGCTTTTTCGTACTGAAGGACAAAAGGGGGTTTTCGAGGCGGGTTCGGAGAGCAATCTGGAACTGGAGGGTTAGAAGTCGTGTAAAATTCCGCAAACTTGGTCGCGGTCTTGCAGGTTGACATGCCTGCCTTTATACTTAAATAAAGGGTTCCCCCGGTTTTCTGTGCATTTCCGGGTTAGTGTGGTTTTTCTAGAGTCTAGAGTTTGTCCACTCT

1. **Apple stem grooving virus complete genome, isolate HPKu-2. Accession number: LT160740.1 (6458 bp).**

ATTTAACAGGCTTAATTTCCGCGCTTTACGTCAATGGCTTTCACTTACAGAAATCCCCTCGAAATTGCAATCAACAAGCTTCCAAGTAAGCAGTCTGATCAACTGCTTTCTTTAACCACAGACGAGATTGAAAAGACCTTAGAAGTGACTAATCGCTTCTTCTCTTTTTCTATCACTCCAGAAGATCAAGAATTGTTGACCAAGCATGGTCTAACACTTGCACCCATTGGGTTTAAGTCACATTCCCATCCAATATCCAAAATGATAGAAAATCACCTTCTGTACATATGTATTCCAAGTCTTTTATCTTCTTTTAGATCCGTAGCCTTTTTCTCGCTTAGAGAAAATAAAGTCAACAATTTTCTCAAGATGCACTCAGTTTTTTCACATGGAAAAATTAAGTCTCTAGGCATGTACAACGCCATCATTGATGGAAAAGATAAATACAGGTATGGCGAAGTTTCTTTTTCTTCCTTTAGAGATAGGGTGATTGGTCTCAGGGATCAGTGCCTCATGCGTAATAAATTTCCGAAAGTTCTCTTTCTCCACGATGAACTTCATTTCCTAAGCCCTTTCGACGTTGCCTTTCTATTTGAGACAATCCCAGAAATTGATAGAGTCATAGCAACTACTGTTTTTCCGATTGAACTGCTTTTTGGGGACAAAGTTTCAAAAGAGCCCAGGGTTTACACCTACAAGGTCCATGGTTCCTCCTTTTCATTCTATCCAGATGGTGTAGCCTCTGAGTGCTATGAGCAAAATCTTGCAAACTCCAAATGGCCGTTCACTTGTAGTGGAATTCAGTGGGCTAACAGGAAAATAAGGGTCACTAAGTTGCAGAGCCTCTTTGCTCATCACGTATTTTCATTTGATAGGGGCAGGGCTTGCAACGAATTTAACCACTTTGACAAGCCGAGTTGTCTCCTTGCAGAAGAAATGCGCCTTTTGACCAAAAGGTTTGATAAGGCAGTCATTAATAGGAGTACGGTTTCTTCTTTGAGCACTTATATGGCTTGTCTCAAGACCGCCAATGCAGCTTCTGCTGTCGCCAAGCTGAGGCAACTAGAGAAAAGGGACCTCTATCCGGATGAACTAAACTTCGTCTATTCTTTTGGAGCGCATTTCAAAAATTTTGGAATGAGGGACGATTTTGATGTGTCAGTCCTGCAATGGGTGAAGGACAAATTCTGTCAAGTTATGCCTCACTTCATTGCTGCCAGTTTCTTTGAGCCAACTGAGTTCCATTTGAATATGCGAAAATTGTTGAATGACCTGGCCACTAAAGGGATAGAGGTTCCACTTTCAGTAGTGGTTTTGGATAAGGTAAATTTTATAGAGACCAGATTTCATGCCAGGATGTTCGAAATTGCCGAAGCTATTGGAGTCAGTTTAAGTCTACTTGGAAAAAGGTTTGACTATGAAGCTGAAAGTGAAGAATATTTTTCAGAAAATGGCTATCTTTTTATGCCTTCAAGGACAAATCCAAGTAGAAACTGGATCTTAAACTCTGGGTCTGTCAAAGTTGATTTCCAGAAGTTGCAGAGGGCAAGGAGGTTCAGATTGAGAAGAGATTTCGTTGATTTGATCGCTAGAAGGGAAACCCCTCGAAAGCAACTATTCCTCGAGTCAGCTCAAACTCTCCAATCAACGTCTAAAAGTGTGGAGGAGAACATGGACAGTGGAGTCAAATCCGACGAAAGCAGTAAGAAAAAACAGTCACTCGAGGTGCCCTGTGTCCCGATGTCCACTGAAGATGGGCAAGGTTTTGAAGGTTCAATTCCAATTGACTTGATAAATTCCTTCGAGCCAGAAGTAATCAGACTTCCGAAAAGGAAGAGAAAGAATGATTGTGTTTTTAAAGCCATCTCTGCATACCTCGGCATTGATGTTCAGGACCTCTTGAACTTTTTGGTGAATGAAGACATATCCGATGAGTTGCTTGAATGCATAGATGAAGATAAAGGCCTGTCGCACGAGATGATAGAGGAAGTTCTGGTGACGAAAGGGATTTCTATGGTCTACACTTCAGACTTCAAAGAGATGGCAGTTCTCAACAGGAAGTATGGAGTTAATGGAAAAATGTACTGCACAATTAGGGGGAATCATTGTGAGCTGAGTTCAAAAGAATGCTTCATCAGATTGCTGAAGGAGGGTGGTGAAGCACAAACGTCCAATGAGAATTTGAATGCTGACTCCTTGTTTGATCTTGGAAAGTTCGTGCACAACAGAGAAAGGGCCACAAAATTGGCAAAATCAATGGCTAGGGGCACAACCGGTCTTTTGACTGAATTTGATCCAGAATTTTGTAAAGGTATGGTCACACTTTCGGAAATGTTTCCGGAGAACTTCTCCTCTGTTGTCGGACTGAGGTTGGGCTTCGCTGGTTCCGGAAAAACCCATAAAGTTCTTCAATGGATCAACTACACACCAAGCGTCAAAAGAATGTTTATTAGTCCAAGGAGAATGCTGGCCGACGAGGTTGAAATTCAATTAAGAGGGACATCTTGTCAAGTTCATACTTGGGAAACAGCTCTCAAAAAGATTGATGGAACCTTCATGGAGGTCTTTGTGGATGAAATTGGTCTTTACCCACCTGGATATCTCACTCTTCTACAAATGTGTGCTTTCCGAAAGATCGTTAAGGGGCAAAGTGAAAAGTTTTTAAGAGGAAAGCTTGCTGAACTTTCGAAGACCTGTTTGAATATAAGGTGTTTTGGTGACTCATTGCAAATGAGGTATTATTCTGCAGAGGATACAAACCTTCTAGATGGAACACATGAGCCTGATCTAATGGTCCAAACCATCAAACACAAGTACCTTCTTCAAGGGTATAGGTTCGGCCAATGGTTCCAAGAGCTGATAAACATGCCAACTAGAGTGGACGATTCGAAATTTTCAAGGAAATTTTTTGCAGACATTTCTAGTGTCAAAACCGAAGATTATGGACTCATACTTGTCGCCAAGAGGGAAGACAAGGGGACGTTTGCCGGGAGAATACCTGTGGCTACGGTCAGCGAATCACAGGGAATGACTATAAGCAAGAGGGTGCTAATATGCTTAGATCAAAATATATTTGCCGGGGGAGCCAATGCCGCCATAGTTGCGATCACTAGATCAAAAGTCGGGTTTGACTTTATTCTCAAAGGGAATTCACTAAAAGAGGTGCAGAAAATGGCCCAAAAAACTTTGTGGCAATTCGTACTTGAAGGAAAGACCATACCGATGGAAAGAATTGTAAATATGAACCCCGGAGCCAGCTTTTATGAGAGCCCCTTAGACGTTGGCAACTCGTCCATCCAGGACAAAGCCTCACATGACTTGTTCATAATGCCCTTTATCAATTTAGCTGAAGAAGAAGTTGACCCTGAGGAAATCGTCGGAGATGTTATTGAACCAGTTGAGTGGTTCAAATGCCATATACCGGTCTTTGATACAGACCCGATGCTTGCAGAGATATTTGACAGAGTGGCAGCAAAGGAAAAGAGAGAATTTCAGTCCATACTTGGGCTTTCAAATCAATTTCTTGATATGGAAAAAAATGGATGCAAAATCGATATTTTGCCTTTTGCTAGACAGAATGTTTTCCCTCACCATCAAGCTTCAGATGACGTCACCTTTTGGGCAGGTGTTCAGAAGAGGATAAGGAAGTCAAATTGGAGACGGGAAAAGTCGAAATTTGAGGAGTTTGAAGCTCAGGGGAAGGAATTGCTCAGTGAATTCATGTCAATGTTACCCTTTGAATTCAAGGTGAACATCAAGGACATTGAAGAAGGTGAAAAAAGCTTTCTTGAAAAGAGAAAGTTAAAGTCTGAAAAGATGTGGGCCAACCACTCAGAAAGATCTGACATTGACTGGAAACTTGACCATGCCTTTCTATTCATGAAATCTCAATATTGCACAAAGGAAGGCAAAATGTTCACTGAGGCAAAAGCTGGTCAAACCTTGGCGTGTTTCCAGCACATTGTTCTTTTTAGGTTTGGCCCTATGCTGAGGGCAATTGAGAGTGCTTTCCTGAGGAGTTGTGGGGAATCATATTACATACATTCCGGGAAGAACTTTTTCTGCCTGGACAGTTTTGTGACAAAGAATGCAGACGTGTTTGACGGATTTTCCATTGAATCTGACTACACGGCTTTTGACTCGTCCCAAGACCACGTGATTTTGGCATTTGAAATGGCACTGCTTCAGTACTTGGGTGTGTCAAAGGAATTCCAGTTGGATTACTTGAGGTTGAAGCTGACGCTCGGATGCAGACTCGGGTCACTGGCAATCATGAGGTTCACTGGGGAATTCTGCACTTTTTTGTTCAATACATTTGCAAACATGCTTTTCACCCAGTTGAAATACAAAATTGACCCGAGGAAGCATAGGATTCTATTTGCTGGAGATGACATGTGCTCTCTAAGTTCTCTCAAAAGAAGAAAAGGGGAAAAGGCAACAAGACTGCTGAAGAGCTTTTCTTTAACTGCTGTGGAAGAGGTACGGAAGTTTCCAATGTTCTGTGGGTGGTACTTGAGCCCATATGGGATTATCAAGTCTCCAAAGTTGTTGTGGGCCAGGATCAAGATGATGAGTGAAAGACAACTTTTAAAGGAATGTGTGGATAACTATCTGTTTGAAGCTATATTTGCCTACAGATTAGGTGAGAGGCTTTACACAATTTTGAAAGAAGAGGACTTTGAGTATCATTACCTTGTCATAAGGTTTTTCGTTAAAAATTCTAAGTTGCTGACAGGGTTGAGCAAAAGCCTCATTTTCGAAATTGGTGAAGGGATTGGGTCAGAATGGCAATCGTCAATGTCAACCATTTCCTCAAGGAAGTCGAATCAACTGATCTCAGAATTGATGCCATCTCTTCCTCGGAATTGTACAAAGATGCCACTTTTTTCAAGCCGGATGTGCTCAACTGTATCAAGCGATTTGAATCAAATGTCAAGGTGTCTTCAAGGTCAGGTGATGGGCTCGTACTGTCTGATTTCAAACTGTTTGATGACACAGAGATCGACTCCATTAGAAAGAAGAGCAACAAATACAAGTATCTGCATTATGGAGTCATTCTGGTCGGGATTAAGGCTATGCTACCGAACTTTAGGGGAATGGAGGGGAGGGTCATTGTGTATGACGGTGCATGCTTGGACCCAGAAAGAGGTCACATTTGTTCCTACTTATTCAAATTTGAGTCCGACTGTTGTTACTTTGGGCTTAGACCGGAACATTGCCTATCCACAACCGATGCCAAATTGGCTAAAAGGTTCAGATTTAGAGTGGATTTTGATTGTCCACAATACGAGCAGGACACAGAGCTGTTTGCTCTCGACATTGGGGTTGCATACAGGTGTGTCAATTCAGCCAGGTTCTTGGAAACTAAGACTGGTGATTCAGGATGGGCTTCACAGGCGATCAGCGGCTGTGAGGCACTTAAATTCAATGAAGAAATCAAGATGGCAATCCTGGATCACAAATCACCGCTGTTTCTGGAAGAAGGTGCACCAAACGTGCATATTGAAAAGAGAATGTTTAGGGGTGACAAAGTCAGAAGGTCACGCTCTATCTCTGCAAAAAGGGGGCCAAACTCAAGGCTACAGGAAAAGAGAGGATTTAGGTCCCTCTCAGCTAGAATCGAAAGATTTGGAGAAAATGAGTTTGGAAGACGTGCTTCAACAAGCGAGACGCCACCGGGTAGGAGTGTATCCTTGGAAGACTCACATAGACCCGGCAAAGGAACTTCTGACGGTTCCTCCCCCTGAAGGATTTAAAGAAGGTGAAAGCTTTGAAGGTAGGGAGCTCTACCTTCTTCTATGTAATCACTACTGTAAATATTTATTTGGTAATATTGCTGTTTTCGGGTCATCTGACAAGACCCAGTTTCCTGCTGTTGGATTTGATACCCCTCCGGTTCATTTTAATTTGACAACGACCCCGAAAGAAAGGGAGACTGAAGAGCAAAAGAAGGCCAGAGAGGGATCGTCTGGTGAAAAAACAAAAATTTGGAGAATTGACTTGTCAAACGTTGTACCTGAATTGAAAACCTTTGCTGCCACTTCTAGGCAGAACTCTTTGAACGAATGTACGTTCAGGAGGCTTTGTGAACCATTTGCTGATCTAGCACGTGAATTTCTACATGAAAGATGGTCCAAAGGACTGGCCACCAACATTTACAAGAAATGGCCCAAAGCTTTTGAAAAAAGTCCATGGGTGGCATTTGACTTTGCCACTGGTCTAAAAATGAATCGCTTAACACCTGATGAGAAGCAGGTGATTGATAGAATGACAAAGAGGCTTTTTCGTACTGAAGGACAAAAAGGGGTTTTCGAGGCAGGTTCGGAGAGTAACCTCGAACTGGAGGGTTAGGAGTCGTGTGAAATTCCGCAAACTTGGTCGCGGTCTTGCAGGTTGACATGCCTGCCTTTATACTTAATTAAAGGTTTCCCCCGGTTTTCTGTGCATTTCCGGGTTAG

1. **Apple stem grooving virus isolate Shandong-1, complete genome. Accession number: MK481964 .1 (6493 bp).**

ATTTAACAGGCTTAATTTCCGCGCTTTACGTCAATGGCTTTCACTTACAGAAACCCCCTCGAAATTGCAATCAACAAACTTCCTAGTAAGCAGTCTGATCAACTACTTTCCTTGACCACCGACGAGATTGAAAAGACCTTAGAAGTGACCAACCGCTTCTTCTCTTTTTCAATCACACCAGAAGATCAAGAATTGTTGACCAAGCATGGTCTAACACTTGCACCTATAGGGTTTAAGTCACACTCCCATCCAATATCCAAAATGATAGAAAATCATCTCTTGTATATATGTATTCCGAGTCTTTTATCCTCCTTTAAGTCAGTTGCCTTTTTTTCACTTAGGGAGAACAAAGTAGACAGCTTTCTTAAGATGCATTCAGTCTTTTCCCACGGAAAAATTAAATCTTTAGGGATGTACAATGCTATAATTGATGGGAAAGATAAATATAGGTATGGTGATGTAGAGTTTTCATCTTTTAGGGATAGAGTGATTGGTCTTAGAGATCAATGCCTTACACGTAATAAATTTCCAAAAGTTCTGTTTCTTCACGACGAGTTGCACTTTCTAAGTCCATTTGACATGGCTTTCCTTTTTGAGACAATCCCAGAAATTGATAGAGTTGTTGCAACCACAGTTTTTCCAATAGAACTTTTATTTGGGGACAAGGTCTCTAAGGAACCTAGGGTTTATACCTACAAGGTCCATGGTTCCTCCTTTTCATTCTATCCAGATGGTGTAGCCTCTGAGTGCTATGAGCAAAATCTTGCGAACTCTAAATGGCCGTTCACTTGTAGTGGAATTCAGTGGGCTAACAGGAGAATAAGGGTTACTAAGTTGCAGAGCCTCTTTGCTCATCACGTATTTTCATTTGATAGAGGCAGGGCTTGCAACGAATTTAACCACTTTGACAAGCCGAGTTGTCTCCTTGCAGAAGAAATGCGCCTTTTGACCAAAAGGTTTGATAAGGCAGTCATTAATAGGAGTACGGTTTCTTCTTTGAGCACTTATATGGCTTGTCTCAAGACCGCCAATGCAGCTTCTGCTGTCGCCAAGCTGAGGCAGCTAGAGAAAAGGGACCTCTATCCGGATGAACTAAACTTTCGTTTATTCCTTTGGGGAGCATTTCAAAAATTTTGGAATGAGGGACGATTTTCATGTGTCAGTCCTGCAATGAGTGAAGGACAAATTTTGTCAAGTTATGCCTCACTTCATTGCTGCCAGTTTCTAAGAGCCAACTGGGTTCCATTTGAATATGCGAAAATTGTTGAATACCTGGCCACTAAAGGGATAGAGGTTCCACTTTCAGTAGTGGTTTTAGATAAGGTAAATTTTATAGAGACCAGATTTCATGCCAGGATGTTCGAAATTGCCGAAGCTATTGGAGTCAGTTTAAGTTTACTTGGAAAAAGGTTTGACTATGAGGCTGAAAGTGAAGAATATTTTTCAGAAAATGGCTATCTTTTCATGCCCTCAAGGACGAATCCAAGTAGAAACTGGATCTTAAACTCTGGGTCTGTCAAAATTGATTTCCAGAGGTTGCAGAGGGCAAGGAGGTTCAGATTGAGAAGAGATTTCGTTGATTTGATTGCCAGAAGGGAAACCCCTCGAAAGCAACTATTCCTAGAGTCAGCTCAAACTCTCCAATCAACGTCTAAAAGTGTGGAGGAGAACATGAACAGTGGAGTCAAATCCGACGAAAGCAGTAGAGAAATACAGTCACTCGAGGTGCCCTGTGTCCCGATGTCCACTGAAGATGGGCAAGGCTTTGAAGGTTCAATTCCAATCGACTTGATAAATTCCTTCGAGCCAGAAGTAATCAGACTTCCGAAAAGGAAGAGGAAAAATGATTGTGTTTTTAAAGCCATCTCTGCACACCTCGGCATTGATGTTCAGGACCTCTTGAACTTCTTGGTGAATGAAGACATATCCGATGAGTTGCTTGAATGCATAGATGAAGATAAAGGCCTGTCGCACGAGATGATAGAGGAAGTTCTGGTGACAAAAGGGATTTCTATGGTCTACACTTCAGATTTCAAAGAGATGGCAGTTCTCAACAGGAAGTATGGAATAAATGGAAAAATGTACTGCACAATCAGGGGAAATCATTGTGAGCTGAGTTCAAAGGAATGCTTCATCAGACTGCTGAAGGAGGGTGGTGAAGCACAAATGTCCAATGAGAATCTGAATGCTGACTCCTTGTTTGACCTTGGAAAGTTCGTGCACAACAGAGAAAGGGCCACAAAATTGGCAAAATCGATGGCTAGGGGCACAACCGGTCTTTTGACTGAATTTGATCCAGAATTTTGTAAAGGTATGGTCACACTTTCGGAAATGTTTCCGGAGAACTTCTCCTCTGTTGTCGGACTGAGGTTGGGCTTCGCTGGTTCCGGAAAAACCCATAAAGTTCTTCAATGGATCAACTACACACCAAGCGTCAAAAGAATGTTTATTAGTCCAAGGAGAATGCTGGCCGACGAGGTTGAAATTCAATTAAGAGGGACATCTTGTCAAGTTCATACTTGGGAAACAGCTCTTAAAAAGATTGATGGAACCTTCATGGAGGTCTTTGTGGATGAAATTGGCCTTTATCCACCTGGATATCTCACCCTTCTACAAATGTGTGCTTTCCGAAGGATCGTTAAGGGGCAAAGTGAAAGGTTTTTAAGAGGAAAGCTTGCTGAACTTTCGAAGACCTGTTTGAATGTAAGGTGTTTTGGTGACCCATTGCAAATGAGGTATCATTCTGCAGAGGATACAAATCTTCTAGACAAGACACATGAGCTTGATCTAATGGTCAAAACCATCAAACACAAGTACCTTCTTCAAGGGTACAGGTTCGGCCAGTGGTTCCAAGAGCTGATAAACATGCCAACTAGAGTGGACGATTCATACTTTTCAAGGAAATTTTTTGCGGACATTTCTAGTGTCAACACCGAAGATTATGGACTCATACTTGTCGCCAGGAGGGAAGACAAGGGGACGTTTGCCGGGAGAATACCTGTGGCTACGGTCAGCGAATCACAGGGAATGACCATAAGCAAGAGGGTGCTAATATGCTTAGACCAAAATATATTTGCCGGGGGAGCCAATGCCGCCTTTGTTGCGATCACTAGGTCGAAAGTTGGGTTTGACTTTATTCTCAAAGGGAATTCACTAAAAGAGGTGCAGAGAATGGCCGAAAAAACTTTGTGGCAATTCGTGCTTGAAGGAAAGACCATACCGATGGAAAGAATTGTCAATATGAACCCCGGAGCCAGCTTTTATGAGAGCCCCTTAGACGTTGGCAACTCGTCCATTCAGGACAAAGCCTCACATGACTTGTTCATAATGCCTTTTATCAATTTAGCTGAAGAAGAAGTTGACCCTGAGGAAATCGTCGGAGATGTTATTGAACCAGTTGAGTGGTTTAAATGCCATATACCGGTTTTTGATACAGACCCGATGCTTGCAGAGATATTTGACAAAGTGGCAGCAAAGGAAAAGAGAGAATTTCAGTCCATGCTTGGGCTTTCAAATCAATTTCTTGACATGGAAAAAAATGGATGCAAAATCGATATTTTGCCTTTTGCTAGACAGAATGTTTTCCCTCACCATCAAGCTTCAGACGACGTCACCTTTTGGGCAGGTGTTCAGAAGAGGATAAGGAAGTCAAATTGGAGACGGGAAAAGTCGAAATTTGAGGAGTTTGAAGCTCAGGGAAAGGAATTGCTCAGTGAATTCATGTCAATGTTGCCCTTTGAATTCAAGGTGAACATCAAGGACATTGAAGAAGGTGAAAAGAGCTTTCTTGAAAAGAGAAAGTTAAAGTCTGAAAAGATGTGGGCCAACCACTCAGAAAGATCTGACATTGACTGGAAACTTGACCATGCCTTTCTATTTATGAAGTCTCAATATTGCACAAAGGAAGGCAAAATGTTCACTGAGGCAAAAGCTGGTCAAACCTTAGCATGTTTCCAACACATTGTTCTGTTTAGGTTTGGTCCCATGCTTCGAGCAATTGAAAGGGCTTTTCTTAGAAGTTGTGGAGAGTCATACTACATACATTCAGGAAAAAACTTTTTCTGCTTGGACAGCTTTGTGACAAAAAACGCAGAAGTCTTTGATGGGTTTTCTATTGAATCTGACTACACGGCTTTTGACTCATCTCAGGACCACGTGATTTTGGCCTTTGAAATGGCACTTTTGCAGTACCTAGGGGTGTCCAAAGAGTTTCAGTTGGATTATTTGAGATTGAAATTGACCCTTGGCTGCAGACTTGGATCTCTAGCCATTATGAGGTTCACTGGGGAATTCTGCACTTTTTTGTTCAATACATTTGCAAACATGCTTTTCACCCAGTTGAAATACAAAATTGACCCGAGGAAGCATAGGATTCTATTTGCTGGAGATGACATGTGCTCTCTAAGTTCTCTCAAAAGAAGAAAAGGGGAAAAGGCTACAAGGCTGTTGAAGAGCTTTTCTTTAACTGCTGTGGAAGAGGTACGGAAGTTTCCAATGTTCTGTGGATGGTACTTGAGCCCATATGGGATTATCAAGTCTCCAAAGTTGTTGTGGGCCAGGATCAAAATGATGAGTGAAAGACAACTTTTAAAGGAATGTGTGGATAACTATCTGTTTGAAGCTATCTTTGCCTACAGATTAGGTGAGAGGCTCTACACAATCTTGAAAGAAGAGGACTTCGAATATCACTATCTTGTTATTAGATTTTTCGTGAAGAACTCAAAGTTGCTCACTGGTCTTAGCAAAAGTCTAATCTTTGAAATTGGTGAAGGGATCGGGTCTGAATGGCAATCGTCAACGTCAATCACTTCCTCAAGGAGGTCGAGTCAACTGACCTCAAGATTGATGCAATCTCTTCCTCTGAGCTTTACAAAGATGCAACTTTCTTCAAGCCAGATGTGCTCAACTGTATCAAGAGATTTGAGTCAAATGTCAAAGTCTCGTCAAGGTCTGGAGACGGCTTGGTGCTGTCTGACTTCAAACTACTTGATGACACCGAAATTGACTCGATTCGAAAAAAAAGTAACAAGTACAAGTACTTGCACTACGGAGCCATACTGGTTGGAATCAAAGCTATGCTGCCAAATTTTAGGGGAATGGAAGGAAGAGTCATTATATATGATGGGGCCTGTTTGGACCCAGAAAGGGGCCACATCTGCTCATATTTGTTCAAGTTTGAGTCTGATTGCTGTTACTTTGGGCTCAGACCTGAACATTGTCTTTCAACAACGGACGCCAACCTGGCAAAGAGGTTCAGGTTTCGGGTGGATTTTGACTGTCCACAGTATGAGCAAGACACAGAATTGTTTGCCTTGGACATTGGGGTTGCTTATAGATGTGTCAATTCGGCAAGGTTCCTTGAAACCAAGACTGGTGATTCAGGGTGGGCCTCACAGGCGATCAGTGGATGTGAGGCACTTAAATTTAATGAAGAAATCAAAATGGCCATTCTGGATCACAAATCTCCACTGTTTCTGGAGGAAGGTGCACCAAATGTGCACATTGAGAAAAGACTGTTTAGAGGTGACAAAATCAGGCGGTCACGCTCTATTTCAGCAAAGAGGGGGCCAAACTCAGAGCCACAGGAAAGGAGAGGATTTAGGTCCCTCTCAGCAAGAATCGAAAGATTTGGAGAAAATGAGTTTGGAAGACGTGCTTCAACAAGCGAGGCGCCACCGGGTAGGAGTGTATCTTTGGAAGACTCACATAGACCCGGCAAAGGAACTTCTGACGGTTCCTCCCCCTGAAGGATTCAAAGAAGGTGAAAGCTTTGAAGGTAGGGAGCTCTACCTTCTTCTATGTAATCACTACTGTAAATATTTATTTGGTAATATTGCTGTTTTCGGGTCATCTGATAAGACCCAGTTTCCCGCTGTTGGATTTGATACCCCTCCGGTTCATTTTAATTTGACAACGACCCCGAAAGAAGGGGAGACTGAAGAGCAAAAGAAGACCAGAGAGGGATCGTCTGGTGAAAAAACAAAAGTTTGGAGAATCGACTTGTCAAACGTTGTACCTGAACTGAAAACCTTTGCTGCCACTTCTAGGCAGAACTCTTTGAACGAATGTACGTTCAGGAAGCTTTGTGAACCATTTGCTGATCTAGCACGTGAATTTCTACATGAAAGGTGGTCCAAAGGACTGGCCACCAACATTTATAAGAAATGGCCCAAAGCTTTTGAAAAAAGTCCATGGGTGGCATTTGACTTTGCCACTGGTCTAAAAATGAATCGTTTGACACCTGATGAGAAGCAGGTGATCGATAGAATGACAAAGAGGCTTTTTCGTACTGAAGGACAAAAAGGGGTTTTCGAGGCAGGTTCGGAGAGTAACCTCGAACTGGAGGGTTAGGAGTCGTGTGAAATTCCGCAAACTTGGTCGCGGTCTTGCAGGTTGACATGCCTGCCTTTATACTTAAATAAAGGGTTCACCCGGTTTTCTGTGCATTTCCGGGTTAGTGTGGTTTTTCTAGAGTCTAGAGTTTGTCCACTCT

1. **Citrus tatter leaf virus isolate TL101, complete genome. Accession number: MH108976.1 (6494 bp).**

AATTTAACAGCGCTTAATTTCCGCGCATTACGTCAATGGCTTTCACTTACAGAAACCCCCTCGAAATTGCCATCAACAAACTTCCAAGCAAACAATCAGATCAATTGCTTGCTTTAACCACAGACGAGATTGAAAAGACCTTAGAAGTGACTAACCGCTTCTTTTCTTTTTCAATCACCCCAGAGGATCAAGAATTGTTAACCAAACATGGTTTGACACTTGCACCTATTGGATTCAAGTCACATTCCCATCCAATATCCAAAATGGTAGAAAATCACCTTTTGTACATTTGTGTGCCTAGTCTTTTGTCTTCTTTTAAGTCAGTAGCCTTTTTTTCCCTTAGAGAAAGCAAAGTTAATAGCTTCTTGAAAATGCATTCAGTCTTTTCACACGGAAAAATCAAATCTCTAGGTATGTACAATGCCATTATTGATGGAAAAGACAAATATAGATATGGTGATGTCCCCTTCGACTCATTCAGAGATAGAGTGATTGGTCTCAGAGACCAATGCCTTGAACGTAATAAGTTTCCAAAAGTCCTTTTTCTCCACGATGAATTGCACTTCCTTAGCCCCTTTGATGTCGCCTTCTTATTTGAAACGATTCCGGAAATTGATAGGGTTGTTGCGACAACTGTGTTCCCAATTGAACTGCTTTTTGGGGATAAGGTTTCAAAAGAGCCTAGAGTTTATACTTATAAGGTCCATGGCTCTTCTTTCTCTTTTTACCCTGATGGTGTGGCTTCTGAGTGTTATGAACAAAACATTGCAAATTCTAAGTGGCCTTTTACCTGCAGTGGCATTCAATGGGCTAACAGAAAGATAAGGGTTACCAAACTGCAGAGTCTCTTTGCTCACCACGTCTTCTCATTTGATAGAGGGAGGGCCTGCAATGAATTCAATCACTTCGACAAACCAAGCTGTTTGCTTTCTGAAGAAATGCGCCTTTTGACCAAAAGGTTTGACGAAGCAGTGATCAACAGAAGCACGGTTTCTAGTCTTAGCACTTATATGGCTTGTCTTAAGACTGCAAATGCAGCTTCAGCAGTTGCAAAGTTGAGGCAACTAGAGAAAAGGGATCTTTACCCAGATGAGTTAAACTTTGTTTATTCCTTTGGGGAACACTTCAAAAATTTTGGGATGAGGGATGATTTTGACATTTCAGTTTTGCAGTGGGTGAAGGACAAATTTTGTCAAGTCATGCCCCATTTTATCTCCGCCAGTTTTTTTGAACCTACAGAGTTCCACTTAAATATGAGGAAATTATTGAACGATCTAGCAACTAAAGGGATTGAAGTTCCTCTTTCAGTTATCATTTTGGACAAGGTGAATTTCATTGAAACTAGGTTTCACTCCAGAATGTTTGATATTGCCCAGGCTATTGGAGTCAATTTAAGCCACCTTGGACGAAGATTTGACTATGAAACTGAAAGTGAGGAATACTTCTCTGAAGGTGGTTACCTTTTTATTCCCTCAAAGGTCAACCCAGATAGAAACTGGATTCTAAATTCAGACCCACTGAAAGTTGACTATTTTAAATTGAGAAGGGCCAGGAGGTTTAGACTGAAAAGAGATTTCCTCGACCTCATATCTAAAGGGATGGCCCCTAAAAAACAGCTCTTTTTAGAGTTACCCTGCAACTTCGAGTTAAAATCTGAAATCATTAAAGCAAGTTTGACTCAGGGAATGGAGAACAAAGAGGGCAAAGCAGAAGAGGAAACGCCTAGAGTCGCATGTACCCCCATGTCCACTGAAGACGGGCAAGGTTTCGAAGGTTCAATTCCAATCGACTTGATAAATTCCTTTGAACCTGAGAAGATCGAGCTTCCAAGAAGGAAAAGGAAAAATGACTGTGTTTTCAAAGCCATAGCCGCTCATTTGGGGATAGAGTCTCAAGATCTTCTAAACTTTTTGGTCAACGAAGACATATCAGATGAACTGCTAGAATGTATTGAGGAGGATAAAGGTCTGTCACATGAGATGATTGAGGAAGTGCTCATAACCAAAGGTCTTTCAATGGTTTACACTTCTGATTTCAAAGAGATGGCGGTCCTCAATAGGAAGTATGGAGTTAACGGGAAAATGTACTGTACAATAAAAGGTAATCACTGTGAGTTAAGCTCAAAGGAGTGCTTTGTAAGACTGCTAAAGGAGGGTGGTGAGGCTCAAATGTCTAATGAGAATTTGAATGCTGATTCTATGTTTGACCTCGGAAAATTTGTGCACAGCAGGGAACGAGCAGTCAAGCTAGCCAAATCTATGGCTAGGGGGACGACTGGTCTTTTAAATGATTTTGACCCTGGCTTTTGCAAGAGCATGGTAACCCTTTCTGAGTTGTTCCCGGAAAATTTTTCCTCTACCGTTGGCTTAAGGCTGGGTTTTGCTGGTTCTGGAAAAACCCACAAGGTTCTTCAGTGGATTAACTACACTCCAAGCGTGAAGAGAATGTTCATTAGTCCAAGGAGGATGTTGGCTGATGAAGTTGAGGGTCAACTCAAGGGGACATCTTGCCAAGTTCATACTTGGGAGACCGCCCTAAAGAAAATAGATGGGACATTTATGGAAGTCTTTGTTGATGAAATAGGTCTATATCCACCTGGGTATCTCACATTGTTGCAAATGTGTGCCTTTAGGAGAATCGTGAAAGGACAAAGTGAAAGAATTCTGAGAGAGAAACTTGCCGAGCTATCAAAGAGTTGTTTAAACATAAGATGCTTTGGCGACCCACTGCAACTACGATATTACTCCGCTGAAGATGCAAATCTCTTGGACAAAACCCACGAGATTGATTTAATGGTGAGGACAATTAAACACAAGTACCTTTTGCAGGGGTATAGATTTGGCCAGTGGTTTCAAGAACTATTGAGCATGCCCACCAGGATGGACGAATCAAAATTCTCAAGAAGATTTTTTGCTGACATTTCTAGTGTAAAGATTGAGGAGTATGGGCTCATTCTAGTAGCAAAGAGAGAAGATAAAGGAGTTTTCGCCGGAAGGGTGCCAGTGGCCACAGTCAGTGAGTCCCAGGGAATGACAATCAATAAAAGGGTGTTAATATGTTTGGACCAAAATCTTTTTGCTGGGGGGGCCAATGCAGCAATTGTTGCAATAACAAGGTCCAAGACTGGATTTGACTTCATTCTCAAAGGGAATTCACTAAAAGAAGTGCAAAGAATGGCACAAAAAACGATATGGCAATTTATAATCGAGGGTAAAAGCATCCCAATGGAGAGGATAGTGAACATGAATCCCGGAGCAAGTTTCTATGAAAGCCCTTTAGATGTTGGGAATTCATCAATTCAGGACAAGGCTTCACATGACCTGTTCATAATGCCCTTTATTAATCTGGCTGAGGAGGAAGTTGATCCAGAAGAAATCACTGGAGACGTTGTCGAACCTGCCGAGTGGTTTAAATGCCATATACCAGTTTTTGACACGGACCCAATGCTTGCTGAAATCTTTGACAAGGTGGCTGCAAAAGAGAAGCGTGAGTTTCAGTCAATACTTGGTTTTTCCAATCAATTCCTTGACATGGAAAAAAACGGGTGCAAAATAGATATATTGCCTTTTGCAAGGCAAAATGTTTTTCCACACCACCAGGCTTCAGATGATGTCACTTTTTGGGCAGGTGTTCAAAAAAGAATAAGAAAATCTAACTGGAGAAGAGAAAAGTCAAAGTTTGAAGAATTTGAAAGTCAAGGGAAAGAGCTTCTTTCAGAGTTCTTGTCAATGCTGCCATTTGAATTCAAGGTAAACATCAAGGACATTGAAAGCGGAGAAAAAAGCTTTCTGGAGAAAAGGAAGTTGAAGTCCGAGAAAATGTGGGCCAACCATGCAGAAAGGTCTGACATAGATTGGAAGCTTGATCACGTCTTTCTCTTTATGAAATCACAGTACTGCACAAAGGAAGGGAAAATGTTCACTGAAGCAAAAGCTGGCCAAACCTTAGCATGTTTCCAACACATTGTTCTATTTAGGTTTGGCCCTATGCTAAGGGCGATTGAAAGTGCTTTTTTGAGGAGCTGTGGAGATTCATATTATATACATTCTGGAAAGAATTTCTTTTGCCTGGACAGCTTTGTCACAAAAAATGCAGGAGTGTTTGACGGATTCTCCATTGAATCTGACTACACAGCGTTTGATTCATCTCAAGACCATGTCATTCTTGCATTTGAAATGGCATTGCTACAATATCTCGGCGTTTCAAAGGAGTTTCAACTAGACTACTTGAGATTGAAATTGACTCTTGGGTGCAGGCTTGGGTCATTAGCCATAATGAGGTTCACCGGAGAATTCTGTACCTTTTTATTCAACACATTTGCCAACATGCTCTTTACCCAGCTAAAGTACAAAATTGATCCGAGAAGGCATAGAATTTTATTTGCTGGAGACGACATGTGTTCTCTGAGTTCTCTAAGGAGAAGAAGGGGAGAGAAAGCAACAAGGCTCTTGAAAAGCTTTTCCCTCACGGCTGTAGAAGAGGTCAGGAAGTTCCCAATGTTCTGTGGGTGGTATTTGAGCCCATATGGAATAATCAAGTCCCCAAAACTATTATGGGCTAGGATTAAAATGATGAGTGAAAGACAACTTTTAAAAGAGTGTGTAGACAACTACTTGTTTGAAGCAATATTCGCCTACAGATTAGGTGAGAGGCTGTACACAATCTTGAAAGAAGAAGATTTTGAGTATCACTACCTTGTCATTAGGTTTTTTGTCAAAAACTCAAAGCTGTTAACGGGGCTCAGTAAAAGCCTGATTTTTGAGATTGGAGAAGGAATAGGGTCGGAATGGCAATCGTCAATGTCAACCACTTCCTCAAAGAGGTTGAGTCAACAGACCTTAAAATTGATGCGATCTCCTCCTCTGAGTTGTACAAAGATGCAACCTTTTTTAAGCCAGATGTACTCAACTGCATCAAAAGGTTTGAATCAAACGTTAAGGTGTCATCCCGGTCTGGCGATGGATTGGTGCTTTCAGATTTCAAACTCCTTGATGACACTGAAATCGACTCCATTAGGAAAAAGAGTAACAAATACAAGTACCTACACTATGGAGTCATCTTGGTTGGAATCAAAGCAATGCTGCCAAACTTTAGGGGAATGGAGGGGAGAGTTATCGTGTATGACGGAGCTTGCCTGGACCCAGAGAGAGGTCACATCTGTTCGTATTTGTTCAAATTTGAGTCCGACTGTTGTTATTTTGGACTTAGACCTGAACACTGTCTTTCCACAACTGACGCTAATCTGGCAAAAAGATTCAGGTTTAGAGTGGATTTCGACTGCCCACAGTATGAACAGGACACAGAACTGTTTGCTCTTGATATTGGGGTTGCTTACAGATGTGTTAATTCTGCTAGATTCCTTGAAACAAAAACTGGTGATTCAGGGTGGGCTTCGCAAGCTATCAGCGGTTGTGAAGCACTCAAATTCAATGAAGAGATCAAAATGGCCATCTTGGATCACAAATCCCCACTGTTTCTGGAGGAGGGTGCACCAAATGTGCACATTGAAAAAAGACTATTTAGAGGTGACAAAGTTAGAAGGTCACGTTCTATTTCTGCAAAAAGGGGGCCAAACTCAAAACTACAGGAAAAGAGAGGATTTAGGTCCCTCTCAGCAAGAATTGAAAGATTCGGAAAGGATGAGTTTGGAAGACGTGCTTCAACAAGCGAGGCGCCACCGGGTAGGAGTATATCTGTGGAAGACCCATATAGACCCGGGAAAGGAAATTCTGACGGTTCCTCCCCCTGAAGGGTTCAAGGAAGGTGAAAGCTTTGAAGGCAGAGAGCTTTACCTTCTTCTCTGCAATCACTACTGTAAATATTTATTTGGAAATATAGCTGTTTTTGGGTCATCTGATAAGACCCAGTTTCCCGCTGTCGGATTTGATACCCCTCCGGTTCATTACAATTTGACATCGTCCCCAAAAGAAAAGGAAACTGAAGAGCAAAAGAAGGCCAGGGAAGGGACGTCTGGTGAAAAAACTAAAATTTGGAGAATTGACTTGTCAAATATAGTACCTGAATTGAAAACCTTTGCTGCTACTTCTAAGCAAAATTCTTTGAACGAATGTACGTTCAGAAAGCTTTGCGAGCCTTTTGCTGATTTGGCTCGTGAATTTCTTCATGAAAGGTGGTCTAAAGGATTGGCCACAAATATTTATAAGAAATGGCCCAAAGCTTTCGAAAAGAGCCCTTGGGTGGCGTTTGATTTTGCCACCGGTCTTAAAATGAATCGTCTAACACCTGATGAAAAACAGGTGATTGATAGAATGACAAAGAGGCTTTTTCGTACTGAAGGACAAAAAGGGGTTTTCGAGGCAGGTTCGGAGAGTAACCTGGAACTGGAGGGTTAGAAGTCGTGTGAAATTCCGCAAAATTGGTCGCGGTCTTGCAGGTTGACATGCCTGCCTTTATACTTAAATAAAGGGTTCACCCTGTTTTCTGAGCATTTCAGGGTTTATGTGGTTTTTCTAGTACCTGGAGTTTATCCATTT

1. **[Cherry virus A isolate 740M1-C102, complete genome](https://www.ncbi.nlm.nih.gov/nuccore/OR515750.1/). Accession number: OR515750.1 (7410 bp).**

GAACTGAACTACCACAACTTTACAGAATTTCCCAACTGTAAAGAATTCACTTCCACCAATTTCCAAACACTTTCAAAACTCCCAAACACAAGCAAGTGCAATGGCATTTGTGGCTAAATTTGCTGAAGAAAACTACTTCAACTCACTCCCTAGTAATGTCACTGACGCATTTCTTAGGGATGGATTCAATGCTGAGCATAACCGTTTTGAGGTTCTCTCAAGACATTTTGCCTTTGCATTAAAACCAAGTCAGAGAACTTATTTGAATGATTGTGGCATTCAACTAGCACCTATTGCATCCAAGACACACCCACACCCTGTGTCTAAGATCATTGAAAATCATCTCCTCTACTGTGTAGTATCAAATATGATTTCCAACTTCAAGTTTCTGGTCTTCTTAAGCATTAAGGAAGTCAAAGCTGAATACATCTGGAACAAAAATACAGCTGACACAGTTAGAGAGATTTCCAATCGCATTCTTGATATCAAGGATGCTTTCCGTTATGGTCCAACCAATACAGTCAATGGTGGCATAAACAACTTTAGTTTCTTCTGTGCCAACCTCAATAGAAGATTCAACAACAGAGCCATCAAACCTGACTGTTTTTTTATTCATGATGAGGTACATTTTTGGAGCCCAGCTGAACTTTGTGAATTCCTTTTCACTGTTGAGCCCAAAAATGTTTTAGCAACAGTTGTCATTCCACCAGAGTTATTGGAAGGGTTGGATTACAGCTTCAACTCGGTTGCTTATGACTTTAAGAAGGTGGATGGTAACTTGTATTACTTTCCTGACAAATCCAAAGGCAAACCTTACCAACAACCCATGGATCCATGGTTGCTTAAATGCAACAGAATTTCAATGATTAAGAATGGGGAAACTTTTTCCTACTCTATAGGTCTTCTTGAGTCTGTTGGAGCCAATCATCTTTTCTCCTTCCAAAGGAATAAAGTTGTTGAATCTACCCGTTTCTTCAATGACTTTGATTGTCTGGATATGAGAAAATTACTGCCCATAAATGTGGAGAATGGAAAAATCAAGGGGTATAACATCAGGACTTGGGTTTTTAAGAAAATCTTGTCCTATATAGTTTGTCTTAAGAAGGGAGACTCTGAATCATCCTTGGCCAAGCTGAGGCAGCTGAGTGATTCCTCACCAAGCTCTGATGAGCTTCTATTGATTGGTGACTTCTTTGACTTGATGACCAGAGTCAAAATTTTTAATAAACGTAGCCCTTGGAGTTTTCTAAGTGATGCTAAAAATTATGTTGACTCTTGGATCATTCAATCTCCCTTTCTTCGAAGGATTTTTCCAGTGGGCAGTAGGGCCATTACTGAACTCATAAGAGACTGGATTGCTAATGCTGAATCTTTGAAGATTCAAACAACTTGCTCTTCTCTCACTTTTTCAGATTCTTTTGAGATGATAAAAACAGACAGCATATCTGAGCTTGGTGAACACATCTTGGGGAACATCTCTGGTGGATTATCAAGCGCCATTGAAGCCTGTAGAAATTGGTTAACATCAAAGAGATTCTCTGGTGGGAGTTATTCAATGGTTGGTAGAAATGGTCTTCTCGTGGACTCAATGCACCAGAATACTTCTTTCTCTTCTGAAGTATTTGTTGATCTCTTTCCTTCAACCATTAGGCCTGCCTTTTACTCAGACCCTAATTTCAATAAAGTGGAATCCTTTGAGCCTGAATGGGATTTCTTACTTGGGTCTTGGATTTTCAATCATAAGCAAGTTAGATTGTGTTATGAGCCAAGTGATTCACATGCCGACAGTGAGGAGGACATCAATAGAACTTCTGAAACTTCAAGCCCTGAGAAGACTGTTGTAGATTCAAGTTCAGTGTCACCTTTTGTGAGCTCTAATCACGAGGAGGGAACATCATCTCAACAATCTGAAGAAAAAACAATATCTAAGCAGGAGAAAAAGGAGACAAGGAAAAATGATTGTTTCTTTAAAGCAATTGGTGAAACTATTGGGATTCCTGCCAATTCACTAATTGAAAGAATCTTATGTTCAGATTCAGAAGACCTCAAGCCGGTGATTGAGCAGTTGAATCTAGATCACCCAATAAGCTCAAAGTTATTGGAGGTTTGTTGTAAATTCCTGGGATACAGAGTACACATTTATTATGGGGATAGCATAATAAAACTCAATGATGACATCAATATGCATGCAATTCACATTGGAGGGAAGCCAGGTCATCTTTTTTGCATCAACCAGGAAAGATCCAAAATTCCAAAGGATAGCCAGATCAAAGTCCCTGAAGTTGGACCTCAGTCCTTCATTGGATCAATTTTTTCAAAGACTTACGGCTCAGGATCAAGTGCTCCAATTTATTTGGATCAAATTGATATCACAAAGGCTCTGGCCCTTGTTTCAGCCTTTGAGTCTATGAATCTGGGGGTTCGTGTTGACAGGAAAGCCATACTTGAGGGCCAGCTCATATCCAATGGTTTCTTGGCTTTTCTCAAAAGAAAAAATAATGAGGGGCATAAGGTCATTAAAATTAAATCTCTTCCAGTTTACCCATTCATTGGTTTTGCTGGTTCTGGCAAATCCTTTGGATTAACTGAGAAGTTAATCAATGGTGATTGCAGTCAGAATTTTATGTTCACCGCTCCCAGAAAGAAAATCATAGGGCAAATTCATGAGAGAATTGATTCAAGGCAATATGATGACAAGCTAAAAATCAGCAGGAAGAAAAATTTTAGTACTTTTGAGAACACTTTGCTTTCATTAGTCAACAAACCTCTAGTGATCATGGATGAATGTTCCTTGAACCCACCTGGCTTCATTGATTTGGTTCTCATCAAATCTTTGGATTCTATCATCCGGAAGAGCAATAAGGACTTTGATCACTTCTTTTCTTCCAGTGTTTTGTCAGAGGGAATTATTGCGAATGTTGCTTCACCCATTGCTTGCATAGCAGTGACAGGTGATACATTACAGTCCAGTTTCTACTCTGAAAGTTGTGGCAAGCTGATGCAATACAAGAATGACATCAAGACTTTGTGTGCTTTGAGTCATACGAGATTGCCCTATCTTTTTGGATCCAAAAGGTTTGGCTACTTCACTGGCTTCCTCAAGCTTGGATATTACAATCAAATGGAATCAAAGGCTTTCACCATTGACAATATGGAAACTCTACAGAAAGCCATTGGTACCTCCATGGATAAATTCGGGGTTCTAGTTACCTCAAGAGCAGACAAATCAGATTTTGAGCTAGATTTTCCAAATGTCTGCACAATCAATGAAGCACAAGGAAGCACCTTCAATAGTGTCATTTTGATTGTGACAAGGGATTTCTTCTCCAATCCAATTGAGTCAATCATAGTGGCAATAACAAGACATCAGAAAAATCTTTTGATTTATTTTCCTGCTGCTATACAAGGTGAGATGGATTTTCTTAGCAGAAGATTTCCAATTCACTCAAATGTTGTGCTGAAGAACTTTAATGTTTTGGACAACTTGATCAAGGACAAACTGAATCCATTCCAATTGATTCAAGAAGATCCATTTGGTCATGATTTTGAGGTTAAGCTTGAAGGAGATCCTTTCTTGAAGAGTGAATTGAGTTTAGTTAACGAGATTAAATTGCAACAGATTGAAGAAAATTCAATTGAATTTAAGGAAAATCTCAAAACTCATTTACCGATATCATACAGCGGGCTGTGGAATCTGGAGATCAGTGAGATGAGGGCCAGAGAAGACAGAGAATTTAAAAAATTTGGTGTTGGATGGAGTAAACAATTTAAAGATGAGCCAAATCAGAAAGATCAGGTGGAAGATAATTGTGCGATGCTCCCTGAAGCTGTTTTCCCACGACACTTTGCTAATGATGATTTAACTTTCTGGAGTGCAGTCAAAAGAAGATTGGTTTTCAAAAACCCATTGAGTAATGCTCATGATTTTGAAAAGGCCAAACCCTTTGGCAAGGAGTTGTTAAATATATTTCTGAGGAAGGTGCCACTCATGCCTAACTTTGATCAGAGAATGTATGACGAGTCTGTCTCTGAGTTTGAGGAGAAGAAAATAAGTAAAAATGCAGCCATGATAGGAGCTCATCATGACAGGTCAACCACTGACTGGCCAACAAACGAAATCTTCCTCTTCATCAAATCTCAACTATGCACAAAGAAGGAGAAGATGTTTTGCGACGCTAAAGCTGGTCAGACCCTTGCTTGCTTTTCTCATCTCATTCTCTGCAAGTTCGCTCCTCTCAACAGGTACATCGAAAAGAAAGTCACTCAAAGCCTTCCAGGAAATTTCTACATACATCAAAAGAAAAACTTTGATGAGCTTGAAAAATGGGTGAAATCATATGATTTCAGTGGGGTCTGCACAGAGTCAGATTACGAAGCTTATGATGCCTCACAGGATTCTTACACTCTAGCCTTCGAGTATGAGCTTTTGAGGTACCTTGGCGTTTCAAATAGTTTGATTGAGGATTATCTTTACCTGAAAATGCATTTGAATTGCAAACTTGGGAATCTTGCTATAATGCGTTTCACTGGAGAGTTTTGCACCTTCCTATTCAACACATTGACTAACATGTTGTTCACTTTCATGAAGTATGATGTTAGAAAGACTCATGCGATATGCTTCGCAGGTGACGATATGTGTGCTAATGTAAGATTGCCAGAAAATCATGAACACTCAAGCTTACTGAAGAAGTTTTCCTTGAAGGCTAAAGTGGACTTCACTCGCTCTCCAACTTTCTGTGGATGGAACCTCTCAAGGTATGGAATAGTCAAAAAGCCGGAGTTAATTGCTGCAAGATTAGCTGTTGCCAGGCAAAAAGGGGAAGTTAATCTGGTCTTAGATTCATATTTCCTTGAGCATCTCTATGCATACAATAAAGGCGATCACCTTTTTGAAATCTTGAGTGAAAAGGAACTAGAACACCATTACAATCTCACCAGATTCTTTGTGAAGAATGGTAAGCTTTTGAAAGGTGAGTCAAAAAAAAAATTCATGGAAACCAAAGAAATTGAAGGAGGGTTATTTGGGGAGTGTGATTTTGGAAATGATAGTATCTTCAAAGATTACATCAATAAAATGAAAAACAAAGTTGAAATTGATTTATTGAATGAGAGAATTTTGAGGATAAATACTGAGATGAATCAATTTGACCCAAGGGTATACATGATGAATAAGATAGGGTTTGTCACTAGCACATCAATGCTTGAAGCAGGCCAAATTGCATCAAATGAATCCAACCAGTCAAAAAGTCTTCTTCCACAAACAAGCTGGCCATACGATGAGGTGAAACCATACATGCCATTGAGTTTGAGGAATTCTTATGAATCAAGGACAAAAGGCAACAGGCTTCTATTTATCCTGAGAAATCAAAAACATTTATGTGATTTAGGATCAGGCTCTATCTTAGGTTTAAGAGTTTTGGAGGGCTTGAAAATATTCAAGGAGGAATGTCAATCATACCAGTCAAGAAGTTTCTTCAAAGGGTCGCAGGAGACGAATCAAGGATTTTCATTGATGCAATTCGTGCAAAGGACATTTACAGTGATGCAAATGCCTTCAATTCCAAAGTGCTCACAGCTGTTAAAAGATTTCAGTCTTCAATTGCTATACCTGCAAGCTGCACTGGTGAAAGCAATGTTACACAGTTCAATATTTTTGATGAAGTTGAGCTGGAGGCTATCAAAAAAGCCTCTTCTGAGTACTCAATGCTTCATCTTGGGGCAATCATCATATGTGTTACATGCTTTTTCAAGCTTAAAAAGCCGATCAATGGCAGAATTGTGTACTTTGATCCCAGGTTTTTGGACAAAAATGATGCATGCCAAGCAGGCTTTAGTTTCCAGCTGCAAACTGGATCAGCCTATTACCTTTATAGGCCAAATTACCCAATGTCCACACATGATCCAAACATGCACAGGGCTGCTAGAATCAAGTTTGAATTCGATGCAATCAATGTTGTTGACAATTCCCACCTATTCTTCATTGATTTTGGAGTCATGTACCAGCTCAGTAATCAGAGCACTGCAGAGAAGACAACTGCCGCAGATGTTGGGGCACAGTTTCAAGCACTTTTTGGGTCTTCTGGATTACCAAATCCTGAATCCTTTCTTGAGGATGAAGATATTATCAATCCTCCAACTGTGGCACTCATTGACGTCAGTGTTGACCAGAGTTTCAGGAAAGGTGGTTTCTTTAAAGGCCCACCACGTTCAACAAGAGCCAGAAGGTATCATGCCAGAAGCAAGAGACAGGGTTTTGAGTCAATCCCAAAAACTGTTGGTAAAGATCCTAAACAACAAGAAAGAAATTTGTTTAGGTCAAATTCATGTAAGTCTGAAAATTTTCAGTTCAATCCAGAACAGAGGTTTTCAGTTGATCAAGAATTCATCAACAGATTTGATAAGTGTAATTCTCAAAGGAAGGACTCAAATCTCCAGCATGGCTTTGAGCATAGTGGAGCAGAATTACAACGAAATAAGAAGGGGCCTTGGCAATTACATCTGGGAGAACATGATAGATCCGAGGGATCTATTACATCTGACTGCGAAACCAGCGGTGGAGGCATCAGAGGGAGTGGCTGCAACACCAGCGATCACATTATCAGAGAACCAGAGGGCTGTGAAAAATACAATCCGAAATTATTACCTGAGGATAATGTTTGGGAATCTTGCGGTGATGGGTACAAGCGAACAGACAGACTACCCAGGGGAACATCTGGCAATCCCGAGACCAGTGATAGAGAATCAGGAAGCTCTGACTGCACATCTCCCAGCAGGCATGTCATTATTAACTTTTGCCACAAATGTGAAGGCATGGGGTGTGGTTGGTGCAGAAGGTAAATTCGCTGGATTAACTTTCAGGCAGTTGTGTGAACCATTTGCTGAGCAAGCTTATAATTTCTTTAGGGAGAACCATGGAGCTGTCTCATTTATTTACCTCAAGAATCCAGGGGCCTACTTCAATTGCCCAGCTGTCGTCTTTGACTTCAACAAAGGGTTACCTCTGACTATTATCAAAATTGGAAAGAATGCCAATGCAATATCTGCCTGCAATCAGAGACTGTTTAATAGAGAAGGGAAGAAAGCCGTCTTCGCCGCACAAGGCGAAGTAAACTTGAGCTTTGATGCTTAAGTTTTTGTTTGTTGCTTAACTGCCAGAGCAATTAAAAGATGATGGCATAGGTCTTGGATTATGTAGTTAAAACTCAAATCCTTCACCGTCACCAATCAAGGGTGTGTACTTGGTCACTAGAGAAGTAGTGTATGTCTTCTCCAACCTTAAGAGAGGTTAGTTTCTCTTCCCTGGTTTAAGTACTGGGATTTGCATCGTTTGTTTCAAAGGTTCTTTGGATGCATCTTATTTTAGAACCTTTCCGCTTTAAAGTGCTATCTAATTGCACCTTGCTTTAGGAG
